# Deoxygenative Metal Carbenation: A Strategy for Stereocontrolled Olefin Metathesis via an Oxo-Ruthenium Precursor

**DOI:** 10.1021/jacs.6c12046

**Published:** 2026-10-20

**Authors:** Samantha K. Cormier, Harrison C. Max, Eliza-Jayne Y. Boisvert, Michael J. Ferguson, Deryn E. Fogg

**Affiliations:** † Centre for Catalysis Research & Innovation, and Department of Chemistry and Biomolecular Sciences, 6363 University of Ottawa, Ottawa, Ontario K1N 6N5, Canada; ‡ X-ray Crystallography Laboratory, 3158 University of Alberta, Edmonton, Alberta T6G 2G2, Canada; § Department of Chemistry, University of Bergen, Allégaten 41, N-5007 Bergen, Norway

## Abstract

Dithiolene metathesis catalysts have enabled breakthroughs in materials and medicinal contexts in which control of *cis/trans* alkene stereochemistry translates into control over function. Their extreme air-sensitivity, however, impedes widespread uptake. Building on the discovery that air-exposure selectively decomposes the leading catalyst Ru­(S–S)­(H_2_IPr)­(=CHAr) (**Ru-1**a; S–S = 1,2-S_2_C_6_H_2_-3,6-Cl_2_, Ar = C_6_H_4_-2-O^*i*^Pr) into four-coordinate oxo complex **Ru-2** Ru­(O)­(S–S)­(H_2_IPr), we demonstrate that the latter offers convenient access to **Ru-1a** and related catalysts via reaction with phosphorus ylides (Ph_3_P=CHR; R = Ar, Ph, H). This “deoxygenative metal carbenation” (DMC) strategy opens new opportunities in olefin metathesis. Beyond regeneration of **Ru-1a** after decomposition, it enables direct, tunable access to more active on-cycle species (potentially including tagged benzylidenes) from a robust, shelf-stable precursor. In situ generation of these well-defined active species confers metathesis activity and stereocontrol comparable to or better than pristine, meticulously handled **Ru-1a**, and far superior to samples of **Ru-1a** even briefly exposed to air in solution. In eliminating the need for production, shipping, and storage of air-sensitive **Ru-1a**, this advance is anticipated to expand the uptake of olefin metathesis for production of stereodefined alkenes.

## Graphical abstract

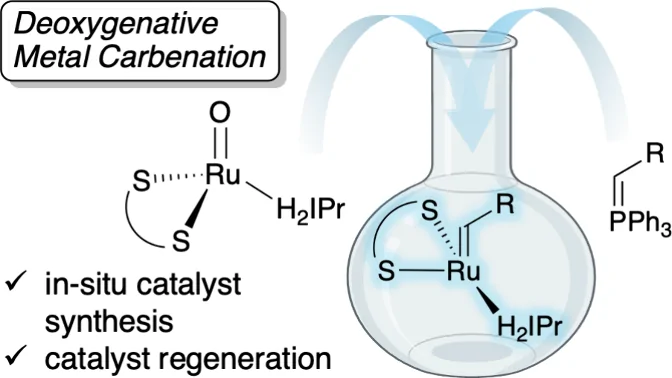

The advent of easily handled catalysts for olefin metathesis has led to the widespread adoption of metathesis methodologies, with transformative impacts on organic synthesis.^1^ Most ruthenium metathesis catalysts, including the second-generation Grubbs and Hoveyda-Grubbs complexes, are remarkably oxygen-tolerant as the precatalysts.^2,3^ The dithiolene (dithiocatecholate, catechothiolate)^4^ catalysts (see, e.g., **Ru-1a**,^5–7^ Scheme 1) are a striking exception.^8,9^ These catalysts can deliver molecules and materials containing stereochemically defined alkenes, for applications relevant to therapeutics, pheromones, agrochemicals, and perfumery,^10–19^ and high-performance “designer” soft materials.^20–23^ They also enable access to conjugated polymers for diverse purposes: wearable electronics, robotics,^24^ and low-bandgap materials for near-IR photodetectors, electrochromic devices, and organic field-effect transistors.^25,26^

**Scheme 1:**
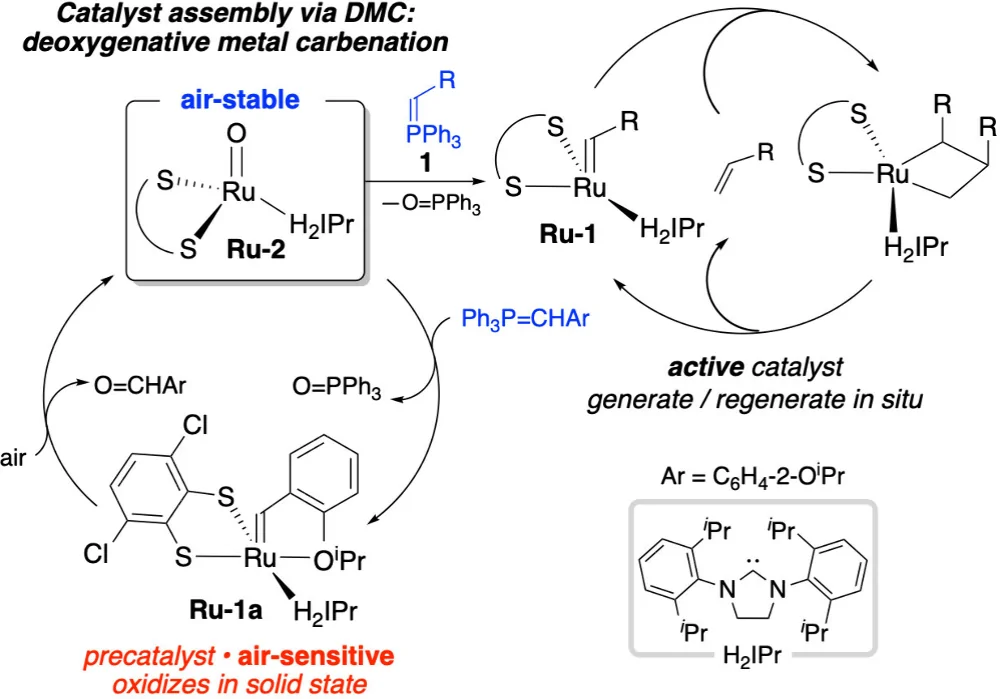
Overcoming the Air-Sensitivity of Precatalyst **Ru-1a** via Deoxygenative Carbenation of **Ru-2**

Extreme air-sensitivity, however—in practical terms, equivalent to that more typical of group 6 metathesis catalysts^7b,27^ —has limited broad uptake. **Ru-1a** and its H_2_IMes analogue degrade into metathesis-inactive oxo complexes (see **Ru-2**, Scheme 1) within minutes in solution in air,^8^ or days in the solid state,^9^ the latter constituting risks for shipping or bench-storage. To mitigate this oxidative sensitivity, Mauduit and coworkers undertook in situ catalyst synthesis by treating the thiol proligand with ZnEt_2_,^28^ for installation on the dichloride catalysts.^29^ Here we describe a complementary approach: we capitalize on the air-stability and ease of handling of oxo complex **Ru-2** to develop a simple, versatile, safer entry to the active catalyst. Reaction of **Ru-2** with phosphonium ylides (via “deoxygenative metal carbenation”, DMC) affords direct access to on-cycle species, repurposing a dead-end decomposition product into a conceptually new, convenient entry point to metathesis catalysis. More broadly, this work points toward opportunities to improve process sustainability in homogeneous catalysis, via efficient regeneration of decomposed catalyst.^30^

Oxidation of molecular olefin metathesis catalysts is widely considered irreversible. The thermodynamic stability of the M=O products, coupled with their rapid engagement in downstream reactions, has been presumed to preclude alkylidene regeneration. From the decreasing strength of the M=O bond across the transition-metal series, however,^31^ and the established O-atom transfer activity of Ru-oxo complexes in (e.g.) water-oxidation catalysis,^32,33^ we questioned whether oxidation is necessarily irreversible in the ruthenium chemistry. We conceived of potential access to metathesis catalysts via a Wittig-like reaction of **Ru-2** with phosphonium ylides (Scheme 1), a pathway inaccessible for more oxophilic transition metals.^34–38^ Deoxygenative carbenation of **Ru-2** is shown to offer access to both known and novel alkylidene complexes: not only the precatalyst **Ru-1a**, but heretofore synthetically inaccessible, on-cycle analogues. The facility with which the alkylidene headgroup can be installed, and the structural diversity of phosphonium ylide reagents, position **Ru-2** as an easily handled, tunable, shelf-stable proxy for oxygen-sensitive **Ru-1a**.

An alkylidene-free route to **Ru-2** is a prerequisite to explore this concept. We thus commenced by developing a 3-step synthesis from commercial **Ru-3** via PPh3 complex **Ru-5** (Scheme 2a). Treatment of the latter with Zn­(S–S)­(en) (S–S = 1,2-S_2_C_6_H_2_-3,6-Cl_2_) affords dithiolene complex **Ru-4**, which on treatment with free H_2_IPr (see Scheme 1) eliminates the *p*-cymene ring to form four-coordinate **Ru-5**. The latter complex was isolated in nearly 70% yield over two steps. The structure depicted is supported by crystallographic analysis (see Scheme 2b, left).^39^ Such four-coordinate ruthenium species remain rare. The low coordination number of **Ru-5** (and indeed **Ru-2**) attests to the protective bulk of the H_2_IPr ligand, illustrated by the steric map in Scheme 2b (right).^40^ The buried volume calculated for the H_2_IPr ligand in **Ru-5** is 45% (cf. 38% for **Ru-2**; Figure S16).

**Scheme 2:**
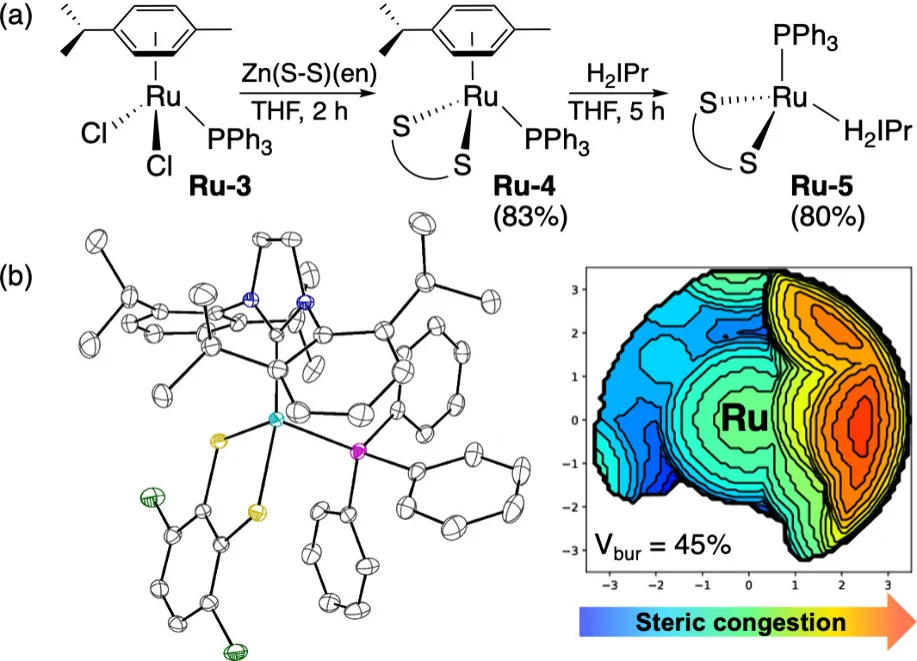
(a) Synthetic Route to **Ru-5**. (b) ORTEP Structure^a^ and Topographic Steric Map of **Ru-5**

a Non-hydrogen atoms shown as Gaussian ellipsoids (30% probability level; C: white; Cl: green; N: blue; P: pink; Ru: aqua; S: yellow; hydrogen atoms omitted).

The final step, oxidation of PPh_3_ complex **Ru-5**, is complete within minutes of exposing to air or dry O_2_ (Scheme 3; for NMR quantitation, see Figures S3–S4). The in situ yield of **Ru-2** is ca. 75%, the balance of material being due to the unexpected urea coproduct **2**. Isolated yields are limited to ca. 40% by degradation on silica or alumina.^41^ Urea **2** is presumed to form via attack of oxygen on ligated H_2_IPr, given that free H_2_IPr is stable to O_2_ ^42^ (like its *N*-mesityl and *N*-^*t*^Bu analogues, H_2_IMes and H_2_I^*t*^Bu).^43,44^ Oxidation of NHC ligands by dioxygen has been reported for complexes of copper^45,46^ and iron,^47^ but to our knowledge, has no precedent in ruthenium chemistry. The observation of NHC oxidation for **Ru-5**, but not prior Ru-NHC complexes—including labile derivatives that would readily afford a binding site for O_2_—implies a role for the dithiolene ligand in promoting NHC oxidation. Of note is the ease with which the oxygen atom in **Ru-2** is abstracted by added PPh_3_ (Scheme 3a, bottom). However, **Ru-2** shows no degradation over 2 months in air in the solid state (Figure S10).

**Scheme 3:**
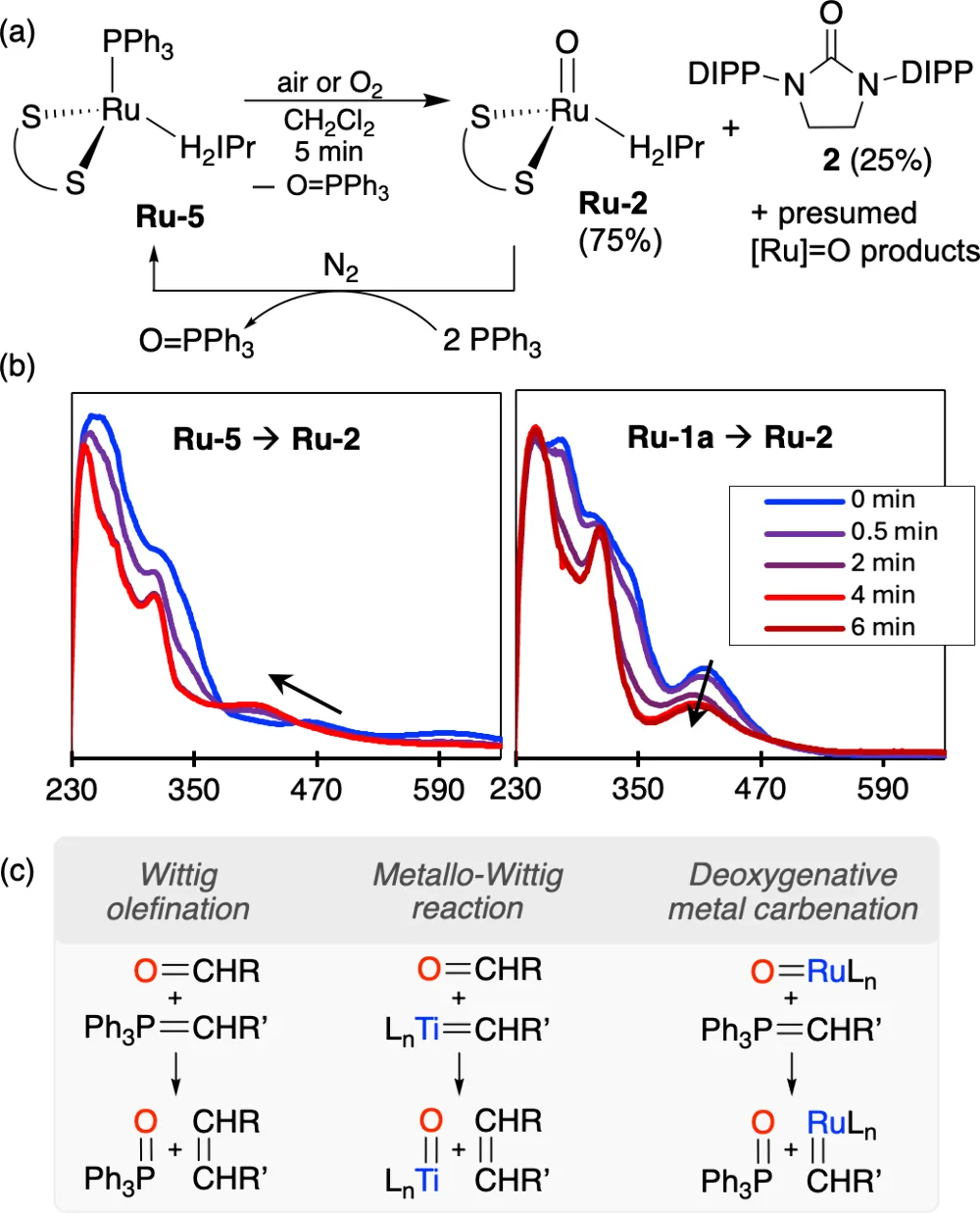
(a) Oxidation and Regeneration of **Ru-5**. (b) UV-vis Spectra (0.1 mM) Revealing Rapid Oxidation of **Ru-5** and **Ru-1a**. (c) Conceptual Framework for DMC

With access to oxo complex **Ru-2** established, we turned to the proposed oxo-to-alkylidene transformation. A long-standing variant on the classic Wittig olefination of carbonyl groups^48^ (Scheme 3c) is the metallo-Wittig reaction,^49^ in which the phosphonium ylide is replaced by a highly polarized, oxophilic metal alkylidene, and exchange is driven by the thermodynamic stability of the M=O bond. In conceiving of deoxygenative carbenation of **Ru-2**, we viewed the Ru=O unit as a potential surrogate for the *carbonyl* group in the Wittig reaction, which could react with the ylide to afford a [Ru]=CHR functionality and phosphine oxide.

We began by establishing that **Ru-1a** can be regenerated from its oxidation product **Ru-2**. Adding a 1.2-fold excess of orange ylide Ph_3_P=CHAr **1a** (Scheme 4a, left-hand plot; Ar = 2-C_6_H_4_–O^*i*^Pr) to a dark green solution of **Ru-2** in C_6_D_6_ caused an immediate color change to yellow-brown. Yields of **Ru-1a** reached 34% within 15 min, and 94% by 3 h. A near-null ^31^P­{^1^H} NMR spectrum was observed, probably due to fortuitous sequestering of the Ph_3_P=O co-product by the LiCl liberated in ylide formation (i.e., reaction of [Ph_3_PCH_2_Ar]Cl with ^*n*^BuLi). A broad ^1^H NMR signal centered at 7.74 ppm (Figure S9), assigned to [Li­(O=PPh_3_)_4_]Cl by analogy to data for the aurate^50^ analogue, indicates low but non-zero solubility in benzene.

**Scheme 4:**
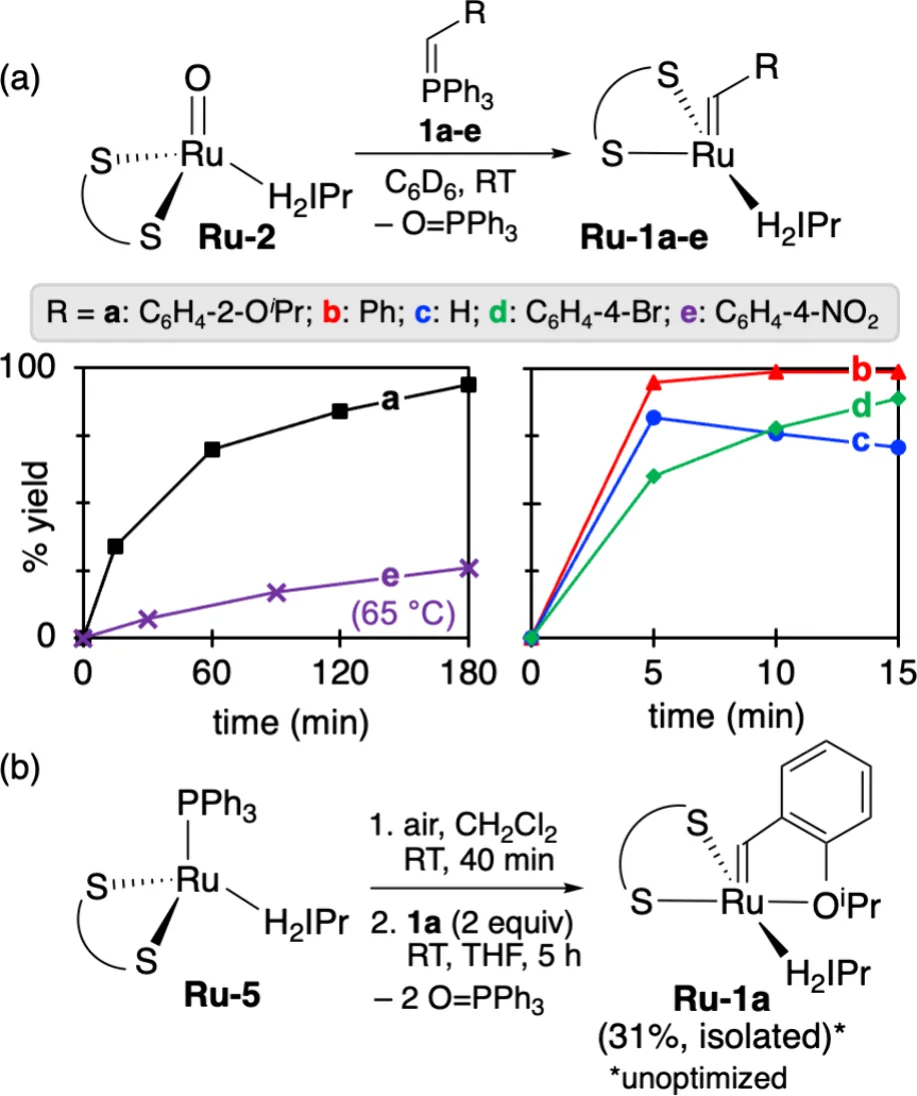
Synthesis of **Ru-1**. (a) Deoxygenative Carbenation of **Ru-2**. (b) One-Pot Oxidation–DMC of **Ru-5**

**Ru-1a** can also be accessed from PPh_3_ adduct **Ru-5** via a one-pot sequence (Scheme 4b) involving oxidation of **Ru-5** to **Ru-2** by stirring in air, then evaporating the solvent and co-dissolving the residue and ylide **1a** in air-free THF. Quantitative conversion to **Ru-1a** was confirmed by ^1^H NMR analysis, but isolated yields were limited to ca. 30% over two steps by the urea-forming side reaction, and degradation of **Ru-1a** on silica.^41^ Importantly, however, the DMC strategy renders precatalyst **Ru-1a** superfluous. Although the chelated benzylidene ligand is invaluable for catalyst synthesis, it is an impediment to catalysis. It necessitates an initial activation sequence of dechelation and rotation of the bulky *o*-O^*i*^Pr benzylidene, followed by a full cycle of metathesis to generate the active species (Scheme S1), with the further risk of ‘boomerang’ recapture.^51^

Rather than optimizing yields of **Ru-1a**, therefore, we explored more reactive, non-chelated complexes. Reaction of ylides **1b/c** with **Ru-2** was quantitative in 15 min for **Ru-1b** or 5 min for **Ru-1c** (Scheme 4a, right-hand plot), as indicated by integration of the [Ru]=C*H*R singlet (16.92 or 14.87 ppm, respectively). Ensuing slow decay of **Ru-1c** is consistent with the reported instability of this methylidene complex.^14,18^ The chemical shift of the [Ru]=C*H*R signals was unaffected by excess O=PPh_3_, suggesting that despite their low coordination number, these complexes bind the phosphine oxide weakly, if at all.

The scope of the alkylidenes accessible via DMC was briefly explored. Electronic constraints (as in the Wittig reaction) arise from the requirement for a nucleophilic ylide, and **Ru-2** did not react with stabilized ylides (RC­(O)­CH=PPh_3_; see the Supporting Information (SI)). The encumbered Horner-Wadsworth-Emmons reagent likewise fails. Benzylidene ylides with electron-withdrawing *p*-substituents are tolerated, however (see **1d**/**1e**, Scheme 4a), although installation of the *p*-nitro derivative **1e** is slow even at 65 °C. “Tagged” benzylidenes are invaluable for many purposes: to accelerate initiation,^52–54^ control solubility,^55,56^ introduce polymer end-groups,^57^ or track catalyst turnon via isotopic^51d^ or fluorophore tags^58–60^ (the latter enabling the remarkable recent detection of metathesis in living cells).^59,60^ Such headgroups are invariably installed via metathesis with functionalized styrenes, and the chelating *o*-O^*i*^Pr group has hence been essential to favor ligation over styrene dimerization. DMC obviates this requirement, as well as the slow initiation imposed by chelation.

Collectively, the rapid, clean reaction with nucleophilic ylides, the low reactivity of the Ph_3_P=O coproduct, and the enhanced metathesis activity of **Ru-1b** open the door to a user-friendly in situ strategy for stereocontrolled metathesis. In situ catalyst generation is ubiquitous in many contexts (cross-coupling offering a prominent example) but has been largely abandoned in olefin metathesis, where it historically delivers low proportions of structurally ill-defined catalysts.^61^ **Ru-1b**, as a rare example of a *well-defined* in situ metathesis catalyst, was tested in stereoretentive self-metathesis of methyl oleate (**3**, Scheme 5a). Accordingly, 2 equiv ylide **1b** was added to **Ru-2** in THF, the solution was stirred for 10 min (guided by the rate curve in Scheme 4a), and substrate **3** was then added. In situ-generated **Ru-1b** conserved the *cis* geometry of the parent alkene to an extent identical to freshly prepared **Ru-1a** handled and stored under rigorously anaerobic conditions, and gave comparable turnover numbers (TONs). Similar results were observed for methylidene catalyst **Ru-1c**, indicating that metathesis out-competes decomposition of the latter. In contrast, a sample of **Ru-1a** exposed to air for ca. 1 min exhibited a nearly 45% drop in TON. (The *E/Z* ratio was unaffected, consistent with our prior finding that **Ru-2** does not promote geometric isomerization of alkenes.)^8^

**Scheme 5:**
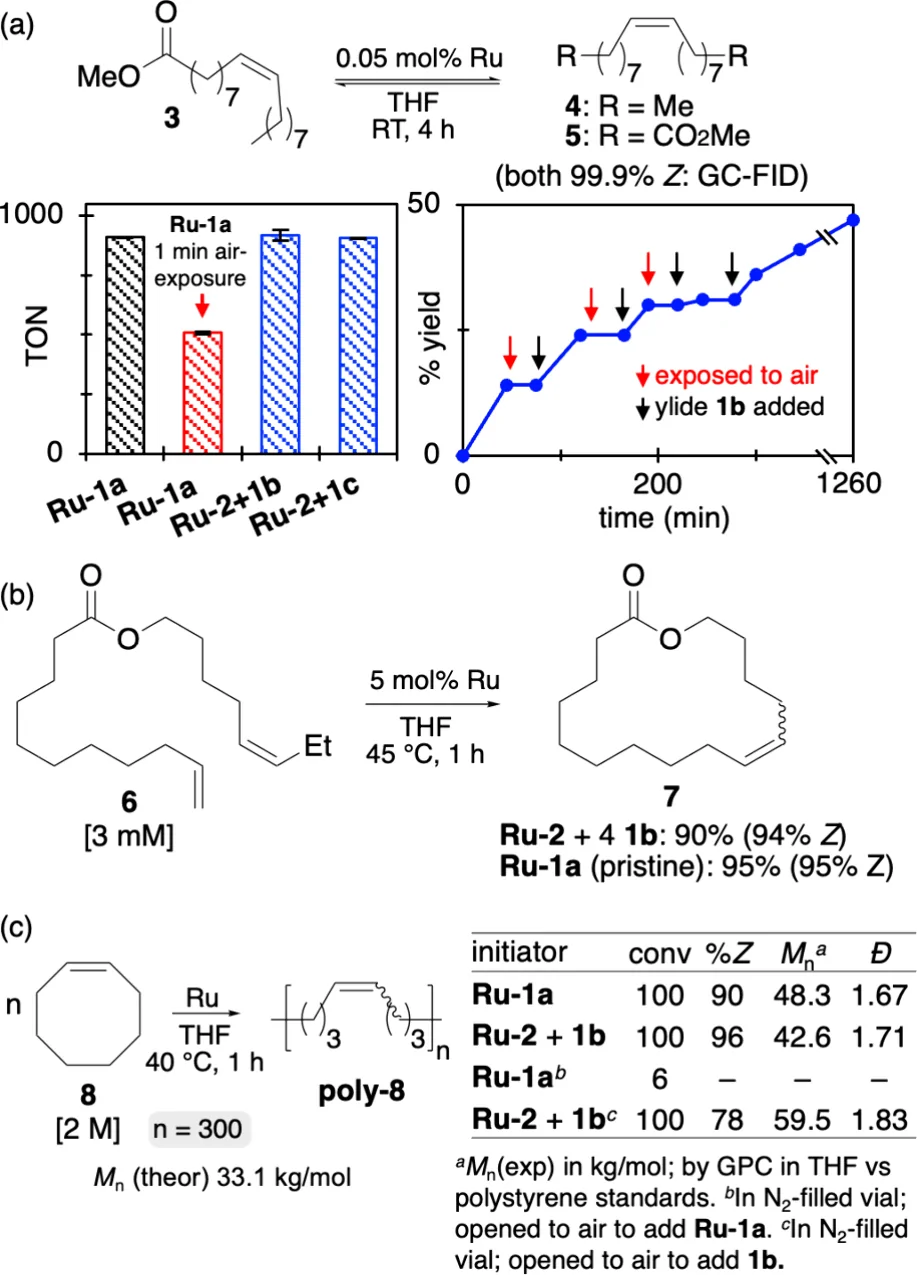
Stereoretentive Metathesis by Isolated **Ru-1a**, vs in Situ-Generated **Ru-1a**–**c**. (a) Self-Metathesis, Showing Oxidation and Rescue of **Ru-1b**. (b) mRCM. (c) ROMP

To probe the capacity of DMC to mitigate the impact of air-exposure, we repeated self-metathesis of **3** with **Ru-1b** under N_2_, terminated reaction at 45 min by opening the flask to air (see first plateau in the time–conversion plot: Scheme 5a, right), then restored inert atmosphere and injected ylide **1b**. Metathesis immediately resumed. Activity was likewise restored over 2 further cycles of air-exposure and ylide introduction. A double bolus of **1b** was added in the final cycle, to compensate for ylide consumption by water accumulated by repeated introduction of humid air. Metathesis proceeded to the equilibrium maximum, confirming that DMC offers a “rescue remedy” to ameliorate the air-sensitivity of **Ru-1**.

We also examined the efficacy of in situ-generated **Ru-1b** in stereoretentive macrocyclization via ring-closing metathesis (mRCM; Scheme 5b). mRCM of the configurationally mobile diene **6** ^11,14,16^ was undertaken as above, and again benchmarked against freshly prepared **Ru-1a**. Comparable *Z*-control was attained with in situ-generated **Ru-1b** and pristine **Ru-1a**.

Finally, stereoretentive ring-opening metathesis polymerization (ROMP) of cyclooctene **8** proceeded to completion within 1 h at 40 °C under N_2_, for both **Ru-1a** and in situ-generated **Ru-1b** (Scheme 5c). Chain lengths were higher than theoretical values, as anticipated,^22,62^ but significantly less so for **Ru-1b**, for which initiation is not impeded by chelation. Of note, **Ru-1b** delivered consistently higher *cis*-content: 96% *Z*, vs 90% Z for **Ru-1a**, or 72% reported for **Ru-1b**·dmso.^62^ Slower metathesis by 5-coordinate initiators may enable competing, non-oxidative decomposition into isomerization-active products. **Ru-2** itself is inactive in ROMP and isomerization (see the SI).

Partial air-exposure revealed more extreme differences. Thus, ROMP failed (ca. 5% yield) when a vial containing cyclooctene **8** in N_2_-saturated THF was rapidly opened to air to add **Ru-1a**, immediately resealed, and heated for 1 h as above. In comparison, adding **1b** in air to a solution of **8** and **Ru-2** led to quantitative ROMP at 1 h. While the resulting polymer was *cis*-rich (78% *Z*), with slightly higher *M*_n_ and dispersity than that under N_2_, DMC evidently protects ROMP activity.

Metal-oxo complexes, although key intermediates in water-oxidation catalysis, have long been viewed as dead-end decomposition products in olefin metathesis. Deoxygenative carbenation of oxo-ruthenium complex **Ru-2** bridges this disciplinary divide. In enabling generation or regeneration of **Ru-2** from a robust, shelf-stable, user-friendly platform, DMC expands access to stereocontrolled metathesis. It also opens new avenues for catalyst diversification via tailoring of the alkylidene headgroup, an otherwise non-trivial undertaking. These advances are anticipated to expand uptake of stereocontrolled olefin metathesis by the broader synthetic and materials community.

Catalyst decomposition, although a long-standing challenge in homogeneous catalysis, is surprisingly little studied. The foregoing demonstrates how mechanistic understanding of decomposition can inform catalyst regeneration, and contribute to more sustainable catalytic processes.

## Supplementary Material



## Notes

Experimental details, NMR and UV–vis spectra, and GC chromatograms (PDF)
