# Supplementary material for: Deoxygenative Metal Carbenation: A Strategy for Stereocontrolled Olefin Metathesis via an Oxo-Ruthenium Precursor
Source: J Am Chem Soc. 2026 Oct 20;148(43):46473–9. doi: 10.1021/jacs.6c12046 (PMC13741830; doi:10.1021/jacs.6c12046)
Supplement: Supplementary file 1 [file ja6c12046_si_001.pdf]

Deoxygenative Metal Carbenation: A Strategy for Stereocontrolled Olefin Metathesis via an Oxo-Ruthenium Precursor

Samantha K. Cormier,[a] Harrison C. Max,[a] Eliza-Jayne Y. Boisvert,[a] Michael J. Ferguson[b] and Deryn E. Fogg*[a,c]

[a]Centre for Catalysis Research & Innovation, and Department of Chemistry and Biomolecular Sciences, University of Ottawa, Ottawa, ON K1N 6N5, Canada. [b]X-Ray Crystallography Laboratory, University of Alberta, Edmonton, AB T6G 2G2, Canada. [c]Department of Chemistry, University of Bergen, Allégaten 41, N-5007, Bergen, Norway

*Corresponding author: dfogg@uottawa.ca, dfo025@uib.no

Table of Contents

S1. Experimental S2
- S1.1 General Procedures. S2
- S1.2 Synthesis of Ruthenium Precursors S2
- S1.3 Oxidation and Deoxygenative Metal Carbenation Reactions S3
- S1.4 *Z*-Stereoretentive Olefin Metathesis S6

S2. NMR Spectra Showing Characterization of New Compounds S10
- S2.1 Ru(S-S)(PPh_3)(*p*-cymene), **Ru-4**. S10
- S2.2 Ru(S-S)(PPh_3)(H_2IPr), **Ru-5**. S14

S3. Representative NMR Spectra and Chromatograms for Reactions S18
- S3.1 Oxidation of Ru(S-S)(H_2IPr)(PPh_3) (**Ru-5**) by Air to Form Ru-2. ..... S18
- S3.2 Oxidation of Ru(S-S)(H_2IPr)(PPh_3) (**Ru-5**) by 8% O_2 in Ar. S19
- S3.3 Reaction of **Ru-2** with PPh_3 under N_2. ..... S20
- S3.4 NMR-Scale Deoxygenative Carbenation of **Ru-2** to form **Ru-1** Complexes. S21
- S3.5 Sequestration of O=PPh_3 as [Li(O=PPh_3)$_4$]Cl S22
- S3.6 NMR Spectrum of Oxo Complex **Ru-2** After 2 Months in Air. S23
- S3.7 NMR Spectra of Polymers. S24
- S3.8 Expanded UV-Vis Spectra for Oxidation to form **Ru-2**. S26
- S3.9 Representative GC Chromatograms for Quantification of Metathesis Reactions. S27
- S3.10 Molecular Weight Distribution for ROMP Polymers S29

S4. Steric Maps S30

S5. X-ray Crystallographic Analysis S31
- S5.1 X-ray Crystallographic Analysis for Ru(S-S)(*p*-cymene)(PPh_3), **Ru-4**. S31
- S5.2 X-ray Crystallographic Analysis of Ru(S-S)(PPh_3)(H_2IPr) **Ru-5**. S41

S6. References S53

S1. Experimental

S1.1 General Procedures. Oxidation reactions were carried out in air, barring one experiment with 8% O_2 in Ar (Linde HIQ Certified 7.96%; contaminants <10 ppm). Other reactions were carried out in an N_2-filled glovebox, unless otherwise noted. HPLC-grade THF, C_6H_6, CH_2Cl_2 and hexanes were dried and degassed using a Glass Contour solvent purification system and stored under N_2 over 4 Å molecular sieves for at least 24 h prior to use. Water content ≤4 ppm was confirmed by regular Karl-Fischer titration. Pentane was distilled over P_2O_5 and stored under N_2 over 4 Å molecular sieves for at least 24 h prior to use. H_2IPr (bis(2,6-diisopropylphenyl)imidazolidin-2-ylidene),[1] $RuCl_2$(*p*-cymene)(PPh_3) (**Ru-3**),[2] [PPh_3CH_2Ar]Cl (Ar = C_6H_4-2-O^{i}Pr),[3] [$PhCH_2PPh_3$]Br,[4] Ru(1,2-$S_2C_6H_2$-3,6-Cl_2)(H_2IPr)(=CHAr) (**Ru-1a**),[5] Zn(1,2-$S_2C_6H_2$-3,6-Cl_2)(en),[6] 3-[(triphenylphosphoranylidene)acetyl]coumarin (**1g**)[7] and (*Z*)-oct-5-en-1-yl undec-10-enoate (**6**)[8] were prepared by the reported methods.

Starting materials were obtained from Sigma-Aldrich, unless otherwise specified. *n*-Butyllithium (*n*-BuLi, 2.5 M in hexanes), 3,6-dichloro-1,2-benzenedithiol (1,2-$S_2H_2C_6H_2$-3,6-Cl_2; abbreviated as S-S in the text below; Sigma-Aldrich or Glentham Life Sciences, 95%), methyl oleate (**3**, 99%), dodecane (≥99%), potassium tris(1-pyrazolyl)borohydride (KTp, 97%; used to quench catalysis),[9] [CH_3PPh_3]Br (98%), [$PhCH_2PPh_3$]Br (Ambeed, 95%), diethyl(2-oxopropyl)phosphonate (Combi-Blocks, 96%), NaH (90%), cis-cyclooctene **8** (Acros Organics, 95%), ethyl vinyl ether (99%), LiHMDS (97%), O=PPh_3 (98%), [$PPh_3CH_2C_6H_4$-4-NO_2]Br (**1e**; Alfa Aesar, 98%), [$PPh_3CH_2C_6H_4$-4-Br]Br (**1d**; Alfa Aesar, 98%), and Ph_3P=$CHCO_2$Et **1f** (95%) were used as received, as were the internal standards used for NMR quantitation. 1,3,5-trimethoxybenzene (TMB; >99%), anthracene (97%), and dimethyl terephthalate (DMT, >99%).

NMR solvents (C_6D_6, CD_2Cl_2) were freeze-pump-thaw degassed 4×, thawed under N_2 on the Schlenk line, transferred to the glovebox, and stored over freshly-activated 4 Å sieves for a minimum of 12 h before use. NMR spectra were recorded on Bruker Avance 300, Avance II 300, Avance III HD 500, or Avance III HD 600 MHz NMR spectrometers at ambient temperature (23 °C). For the purposes of characterization, the highest-field instrument available for each nuclide was employed. (Note: ^{31}P NMR analysis was conducted on the Avance III 500 spectrometer, as the triple-resonance inverse cryoprobe on the Avance III 600 instrument supports only ^{1}H and ^{13}C NMR observation). Chemical shifts (ppm) are referenced to the residual proton or carbon signals of the deuterated solvent (for ^{1}H and ^{13}C NMR, respectively), or external 85% H_3PO_4 (for ^{31}P NMR). Single-crystal X-ray diffraction data were collected on a Bruker Kappa ApexII diffractometer using graphite-monochromated Mo K_α (0.71073) at 100 K.

GPC analysis was conducted by Smith Engineering, Queen's University (Kingston, ON) in THF, using a Waters ARC GPC system equipped with a Waters 2414 refractive index detector, and a set of 4 Waters Styragel HR (0.5, 1, 3, and 4) columns. Polystyrene standards were used as calibrants (M_w range: 266 to 1,100,000 g/mol).

S1.2 Synthesis of Ruthenium Precursors

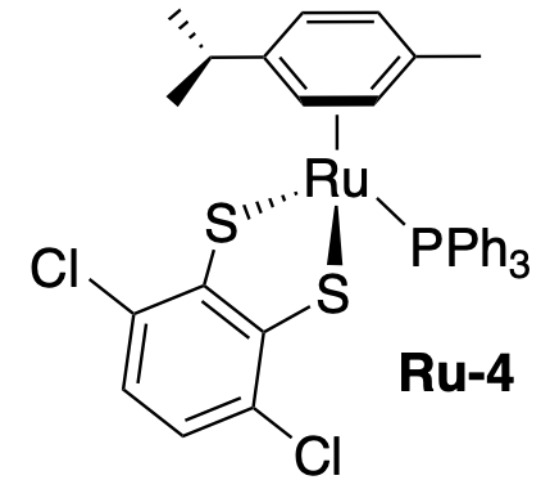

Ru-4

S1.2.1 Ru(S-S)(*p*-cymene)(PPh_3), Ru-4. To a stirred red suspension of $RuCl_2$(*p*-cymene)(PPh_3), **Ru-3** (202 mg, 0.35 mmol) in 10 mL THF was added Zn(S-S)(en) (141 mg, 0.42 mmol, 1.2 equiv). A colour change to dark brown occurred within minutes. Thiolene ligation was complete at 2 h, as judged from disappearance of the ^{31}P{^{1}H} NMR singlet for **Ru-3** and emergence of that for **Ru-4** (24 and 34 ppm, respectively). The solvent was removed under vacuum. The residue was taken up in C_6H_6 (15 mL), filtered

through Celite to remove $ZnCl_2$(en), washed through with further C_6H_6, and stripped to a pink solid. Yield: 200 mg (83%). Ca. 10% free PPh_3 remains, probably because it exists in equilibrium with dimeric [Ru(S-S)(*p*-cymene)]$_2$.[10] For 1D- and 2D-NMR spectra, see Figure S1, Section 3; for X-ray data, see Section 5.

^{1}H NMR (600 MHz, CD_2Cl_2): δ 7.52 (br, 6H, PPh_3), 7.35-7.20 (br, 9H, PPh_3), 6.35 (s, 2H, thiolene C*H*), 5.43 (d, $^3J_{HH}$ = 5.2 Hz, 2H, *p*-cymene sp^2-C*H*), 5.05 (d, $^3J_{HH}$ = 5.2 Hz, 2H, *p*-cymene sp^2-C*H*), 2.73 (sept, $^3J_{HH}$ = 6.7 Hz, 1H, C*H*Me$_2$), 1.72 (s, 3H, C*H*$_3$), 1.12 (d, $^3J_{HH}$ = 6.8 Hz, 6H, iPr C*H*$_3$).

^{13}C{^{1}H} NMR (151 MHz, CD_2Cl_2): δ 148.1, 135.1 (d, J_{CP} = 9 Hz), 133.3 (d, J_{CP} = 47 Hz), 130.5, 130.1, 127.4, 122.3, 113.8 (d, J_{CP} = 10 Hz), 106.2, 94.2 (d, J_{CP} = 5 Hz), 90.9, 30.3, 22.4, 18.4.

^{31}P{^{1}H} NMR (202 MHz, CD_2Cl_2): δ 34.3.

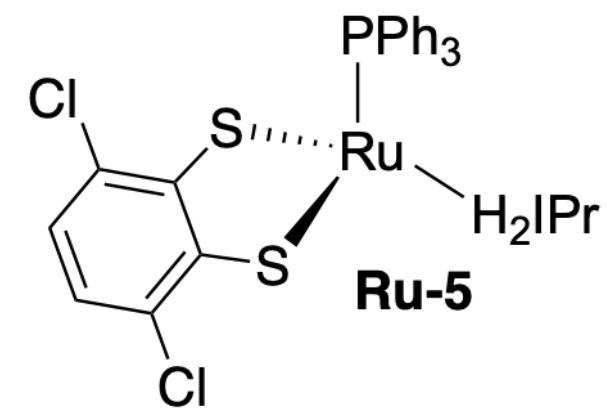

S1.2.2 Synthesis of Ru(S-S)(H$_2$IPr)(PPh$_3$), Ru-5. To a stirred solution of Ru(S-S)(*p*-cymene)(PPh$_3$), **Ru-4** (321 mg, 0.45 mmol) in 20 mL THF was added solid H$_2$IPr (197 mg, 0.50 mmol, 1.1 equiv) as the free carbene. The solution immediately turned from red to brown. Ligand exchange was complete in 5 h, as indicated by the disappearance of the ^{31}P{^{1}H} NMR singlet for **Ru-4** (34 ppm) and emergence of a new singlet for **Ru-5** (73 ppm). The solution was concentrated under vacuum, and pentane was added to precipitate a green solid, which was filtered off and washed with pentane (3 × 1 mL). Yield: 351 mg (80%).

<u>Alternative One-Pot Synthesis of **Ru-5**:</u> To a stirred red suspension of RuCl$_2$(*p*-cymene)(PPh$_3$), **Ru-3** (200 mg, 0.34 mmol) in 10 mL THF was added Zn(S-S)(en) (138 mg, 0.41 mmol, 1.2 equiv). A colour change to dark brown occurred within minutes. ^{31}P{^{1}H} NMR analysis of an aliquot confirmed complete formation of Ru(S-S)(*p*-cymene)(PPh$_3$) **Ru-4** at 2 h. The solution was stripped of solvent. The residue was taken up in 20 mL C_6H_6, filtered through Celite to remove $ZnCl_2$(en), and stripped to dryness. The pink solid was dissolved in 15 mL THF, and H$_2$IPr (196 mg, 0.50 mmol, 1.5 equiv) was added while stirring. Conversion to **Ru-5** was complete by 5 h. The solvent was concentrated and hexanes added to precipitate the product, which was filtered off, washed with hexanes (3 × 2 mL), and dried under vacuum. Yield: 246 mg (75%), reflecting partial solubility of the product in hexanes. For 1D- and 2D-NMR spectra, see Figure S2, Section 3; for X-ray data, see Section 5.

^{1}H NMR (600 MHz, CD_2Cl_2): δ 7.74-7.02 (m, 11H, overlapping sp^2-C*H* of PPh$_3$, H$_2$IPr), 6.96-6.65 (m, 7H, overlapping sp^2-C*H* of PPh$_3$, H$_2$IPr), 6.58-6.44 (m, 5H, overlapping sp^2-C*H* of PPh$_3$, thiolene C*H*), 4.50 (m, 2H, N(C*H*$_2$)$_2$N), 4.18 (m, 2H, N(C*H*$_2$)$_2$N), 3.20 (m, 4H, C*H*Me$_2$), 1.23 (d, $^3J_{HH}$ = 6.6 Hz, 6H, iPr C*H*$_3$), 1.18 (d, $^3J_{HH}$ = 6.6 Hz, 6H, C*H*$_3$), 1.12 (d, $^3J_{HH}$ = 6.6 Hz, 6H, C*H*$_3$), 0.52 (d, $^3J_{HH}$ = 6.6 Hz, 6H, C*H*$_3$).

^{13}C{^{1}H} NMR (151 MHz, CD_2Cl_2): δ 219.9 (d, J_{CP} = 10 Hz), 150.8 (m), 148.2, 142.0 (m), 135.5-134.0 (m), 133.1 (m), 132.2 (d, J_{CP} = 10 Hz), 132.2, 130.1, 127.8, 126.5, 125.6, 121.2, 55.6, 29.5, 29.1, 26.9, 23.0, 22.6. ^{31}P{^{1}H} NMR (202 MHz, CD_2Cl_2): δ 73.1.

S1.3 Oxidation and Deoxygenative Metal Carbenation Reactions

S1.3.1 NMR-Scale Synthesis of Ru(O)(S-S)(H$_2$IPr) (Ru-2) by Oxidation of Ru(S-S)(H$_2$IPr)(PPh$_3$) Ru-5. To a valved NMR tube was added Ru(S-S)(PPh$_3$)(H$_2$IPr) **Ru-5** (150 µL of a 20 mg/mL stock solution in CD_2Cl_2; 0.0031 mmol), ca. 1 mg anthracene as internal integration standard (IS), and ca. 0.5 mL CD_2Cl_2. An ^{1}H NMR spectrum was collected to measure the initial ratio of **Ru-5** to IS. The NMR tube was then opened to air, shaken to displace the N_2 atmosphere

with air, capped, and inverted to mix. Conversion of **Ru-5** to oxo complex **Ru-2** and urea **2** (ratio 3:1) was complete within 5 min (Figure S3; see Figure S4 for the corresponding experiment with 8% O_2 in Ar). Chemical shifts for urea **2** are in agreement with literature values.[11] NMR data for **Ru-2** are provided in Section S1.3.3.

S1.3.2 Tracking Oxidation by UV-Vis Spectroscopy.

Oxidation of Ru(S-S)(H_2IPr)(=CHC_6H_4-2-O^{i}Pr) Ru-1a. A stock solution of **Ru-1a** (10 mg) was made up in CH_2Cl_2 (5 mL; 2.4 mM). A 42 µL aliquot was transferred to a separate vial and diluted to 1.0 mL (final [Ru] = 100 µM). The solution was transferred to a quartz cuvette fitted with a screw-cap lid, which was sealed and wrapped with Parafilm. The cuvette was removed from the glovebox, and a t0 spectrum was acquired immediately after recording a background spectrum for the solvent. The cuvette cap was then removed, a timer started, and air was introduced with a pipette for ca. 15 sec. The cuvette was sealed and shaken to mix. Timepoints were measured at 0.5, 2, 4 and 6 min. No changes were observed after 6 min (Figure S12).

Oxidation of Ru(S-S)(H_2IPr)(PPh_3) Ru-5. Monitored as for **Ru-1a** above, using a 48 µL aliquot of the initial sample of **Ru-5** ([Ru] = 2.1 mM). Timepoints were measured at 0.5, 2, and 4 min. No changes were observed after 4 min (Figure S12).

S1.3.3 Preparative Synthesis of Ru(O)(S-S)(H_2IPr) Ru-2 by Oxidation of Ru-5. Green **Ru-5** (89 mg, 0.092 mmol was added to a 20 mL vial containing a stirbar. The vial was removed to the bench and opened to air. A THF solution of (initially) anhydrous LiCl (2 mL, 0.5 M) was added while stirring. Oxidation of **Ru-5** was complete at 20 min ($^{31}P\{^1H\}$ NMR analysis). The solvent was removed under vacuum, and the residue was purified by silica-gel chromatography in air (9:1 to 7:3 hex:EtOAc), isolating the dark green-brown band. The solvent was removed by vacuum. Green-brown **Ru-2** was precipitated from ca. 0.2 mL EtOAc by adding 1 mL pentane. The precipitate was filtered off, washed with 3 × 0.5 mL pentane and dried under vacuum. Yield: 25 mg (38%). NMR data in C_6D_6 are in general agreement with the reported values[12] in CD_2Cl_2.

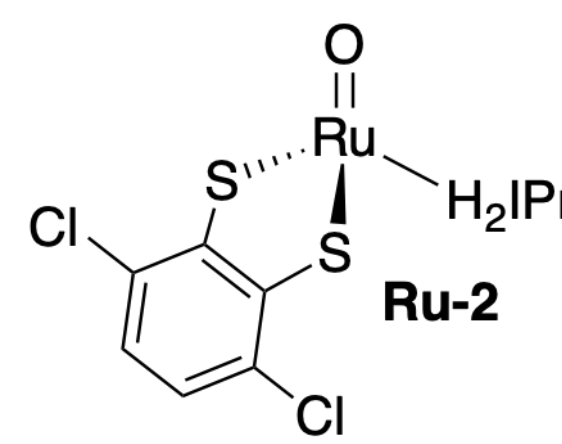

Ru-2

^{1}H NMR (300 MHz, C_6D_6): δ 7.15-7.12 (m, *p*-C*H* of H_2Ipr; obscured by C_6D_5*H*), 6.97 (s, $^3J_{HH}$ = 7.7 Hz, 2H, *m*-C*H* of H_2IPr), 6.79 (s, 2H, thiolene C*H*), 3.64 (s, 4H, N(CH_2)$_2$N), 3.26 (sept, $^3J_{HH}$ = 6.8 Hz, 4H, C*H*Me$_2$), 1.40 (d, $^3J_{HH}$ = 6.8 Hz, 12H, iPr CH_3), 1.08 (d, $^3J_{HH}$ = 6.8 Hz, 12H, iPr CH_3).

S1.3.4 Conversion of Ru(O)(S-S)(H_2IPr)(PPh_3) Ru-2 to Ru(S-S)(H_2IPr)(PPh_3) Ru-5. To a valved NMR tube was added crude **Ru-2**, generated as described above from **Ru-5** (18 mg, 0.019 mmol), ca. 1 mg of anthracene (IS), and ca. 0.5 mL C_6D_6. An ^{1}H NMR spectrum was collected to measure the initial ratio of **Ru-2** to IS. To the tube was added PPh_3 (13 mg, 0.050 mmol, 2.7 equiv). ^{1}H NMR analysis at 15 min revealed quantitative conversion of **Ru-2** to **Ru-5** (Figure S5).

S1.3.5 Procedure for Ylide Generation.

ArCH=PPh_3 1a (Ar = C_6H_4-2-O^{i}Pr): In the glovebox, [ArCH_2PPh_3]Cl (56 mg, 0.13 mmol) was suspended in THF (0.5 mL) in a vial. Addition of ^{n}BuLi (2.5 M, 50 µL, 0.13 mmol, 1.0 equiv) by syringe to the stirred white suspension caused immediate formation of an orange solution. The theoretical 0.25 M concentration is presumed.

PhCH=PPh_3 1b: As for **1a**, using [PhCH_2PPh_3]Br.

CH_2=PPh_3, 1c: As for **1a**, using [CH_3PPh_3]Br.

ArCH=PPh_3 1d (Ar = C_6H_4-4-Br): As above, using [ArCH_2PPh_3]Br (30 mg, 0.059 mmol) and solid LiHMDS (10 mg, 0.060 mmol, 1.02 equiv); 10 min reaction. The theoretical 0.24 M

concentration of **1d** is presumed.

ArCH=PPh$_3$ 1e (Ar = C_6H_4-4-NO_2): As for **1a**, using [$ArCH_2PPh_3$]Br.

S1.3.6 NMR-Scale Synthesis of Ru-1 by Deoxygenative Carbenation of Ru-2. For yields over time, see Table S1. Partial ^{1}H NMR spectra showing the alkylidene signals appear in Figure S7.

R = C_6H_4-2-O^{i}Pr, Ru-1a: In a representative experiment, **Ru-2** (7.5 mg, 0.011 mmol) and ca. 1 mg DMT (IS) were dissolved in ca. 1.5 mL C_6D_6 in a valved NMR tube. After ^{1}H NMR analysis to establish the initial integration ratio of IS to **Ru-2**, ArCH=PPh$_3$, **1a** (50 µL of a 0.25 M solution in THF, 0.013 mmol, 1.2 equiv) was injected. Spectra collected at the times shown in Table S1.

R = Ph, Ru-1b. As for **Ru-1a**, with 6 mg (0.0084 mmol) **Ru-2** in ca. 1 mL C_6D_6, and addition of PhCH=PPh$_3$, **1b** (90 µL of a 0.19 M solution in THF, 0.017 mmol, 2.0 equiv). Quantitative **Ru-1b** at 15 min. The [Ru]=C*H*Ph signal was unaffected by added O=PPh$_3$ (12 mg, 0.043 mmol, 5 equiv).

R = H, Ru-1c. As for **Ru-1b**, with addition of CH_2=PPh$_3$, **1c** (101 µL of a 0.17 M solution in THF, 0.017 mmol, 2.0 equiv). The yield of **Ru-1c** reached a maximum at 5 min, then decreased: see text. The [Ru]=C*H*Ph signal was unaffected by added O=PPh$_3$ (5 equiv).

R = C_6H_4-4-Br, Ru-1d. As for **Ru-1b**, with addition of ArCH=PPh$_3$, **1d** (58 µL of a 0.29 M solution in THF, 0.017 mmol, 2.0 equiv). Formation of **Ru-1d** was quantitative at 60 min.

R = C_6H_4-4-NO_2, Ru-1e. As for **Ru-1b**, with 5 mg (0.007 mmol) **Ru-2** in ca. 1 mL C_6D_6, with addition of ArCH=PPh$_3$, **1e** (84 µL of a 0.17 M solution in THF, 0.014 mmol, 2 equiv). Yield 17% at 48 h (2880 min; Table S1). The corresponding reaction at 65 °C reached ca. 30% yield by 3 h.

Table S1. NMR-Scale Synthesis of **Ru-1** via Deoxygenative Metal Carbenation of **Ru-2**.

Time	**Ru-1a (%)**	**Ru-1b (%)**	**Ru-1c (%)**	**Ru-1d (%)**	**Ru-1e (%)**	**Ru-1e^a (%)**
0	0	0	0	0	0	0
5 min	–	95	82	60	0	–
10 min	–	>99	76	78	0	–
15 min	34	>99	71	89	0	–
30 min	–	–	–	–	–	7
60 min	70	–	–	100	0	–
90 min	–	–	–	–	–	17
2 h	84	–	–	–	–	–
3 h	94	–	–	–	–	26
4 h	–	–	–	–	5	–
16 h	>99	–	–	–	–	–
24 h	–	–	–	–	14	–
48 h	–	–	–	–	17	–

^{a}Reaction at 65 °C.

S1.3.7 Attempted Deoxygenative Carbenation of Ru-2 with Stabilized Ylides.

EtO(O)CCH=PPh$_3$ 1f. As for **Ru-1b** (see S1.3.6), with addition of **1f** (220 µL of a 22 mg/mL solution, 0.014 mmol, 2.0 equiv). No reaction was observed by 24 h.

RCH=PPh$_3$; R = C(O)(coumarin) 1g: As for **Ru-1b**, with addition of **1g** (208 µL of a 30 mg/mL solution, 0.014 mmol, 2.0 equiv). No reaction was observed over 24 h.

[C(O)(OMe)CH=PO(OEt)$_2$]Na 1h: To generate **1h**, C(O)(OMe)CH$_2$PO(OEt)$_2$ (73 μL, 0.42 mmol) was added dropwise to a suspension of NaH in 1.0 mL THF (10 mg, 0.42 mmol, 1.0 equiv) causing homogeneity and effervescence. The solution was stirred for 40 min, after which point 34 μL (0.014 mmol, 2.0 equiv) was removed and injected as above to an NMR tube containing **Ru-2** (5 mg, 0.007 mmol), ca. 1 mg DMT (IS) in ca. 1 mL C_6D_6. No reaction was observed over 24 h.

S1.3.8 Synthesis of Ru-1a from Ru-5 via One-Pot Oxidation–DMC. Solid Ru(S-S)(H$_2$IPr)(PPh$_3$) **Ru-5** (96 mg, 0.099 mmol) was dissolved in non-degassed CH_2Cl_2 (2 mL). The green solution was stirred in air for 40 min, at which point oxidation was complete (null $^{31}P\{^1H\}$ NMR spectrum). The solvent was removed (rotavap) and the flask returned to the glovebox. The residue containing crude **Ru-2** was taken up in THF (2 mL). Red **1a** (0.20 mmol, 2 equiv) in 2 mL THF, generated as described in S1.3.5, was added dropwise over ca. 2 min, with stirring. The reaction was monitored for 5 h, at which point no **Ru-2** remained (NMR). The solvent was removed under vacuum. The residue was taken up in benzene and filtered through silica to remove [Li(O=PPh$_3$)$_4$]Cl$_2$. The silica was washed with 3 mL benzene, and the filtrate was concentrated to ca. 0.5 mL. Addition of hexanes precipitated yellow-brown **Ru-1a**, which was filtered off, washed with hexanes (3 × 1.5 mL) and dried under vacuum. Yield: 26 mg (31% based on **Ru-5**).

S1.4 *Z*-Stereoretentive Olefin Metathesis

Scheme S1 illustrates the steps required for initiation of **Ru-1a** (see main text): dechelation of the ether donor, benzylidene rotation, and a full cycle of metathesis to eliminate the styrenyl ether.

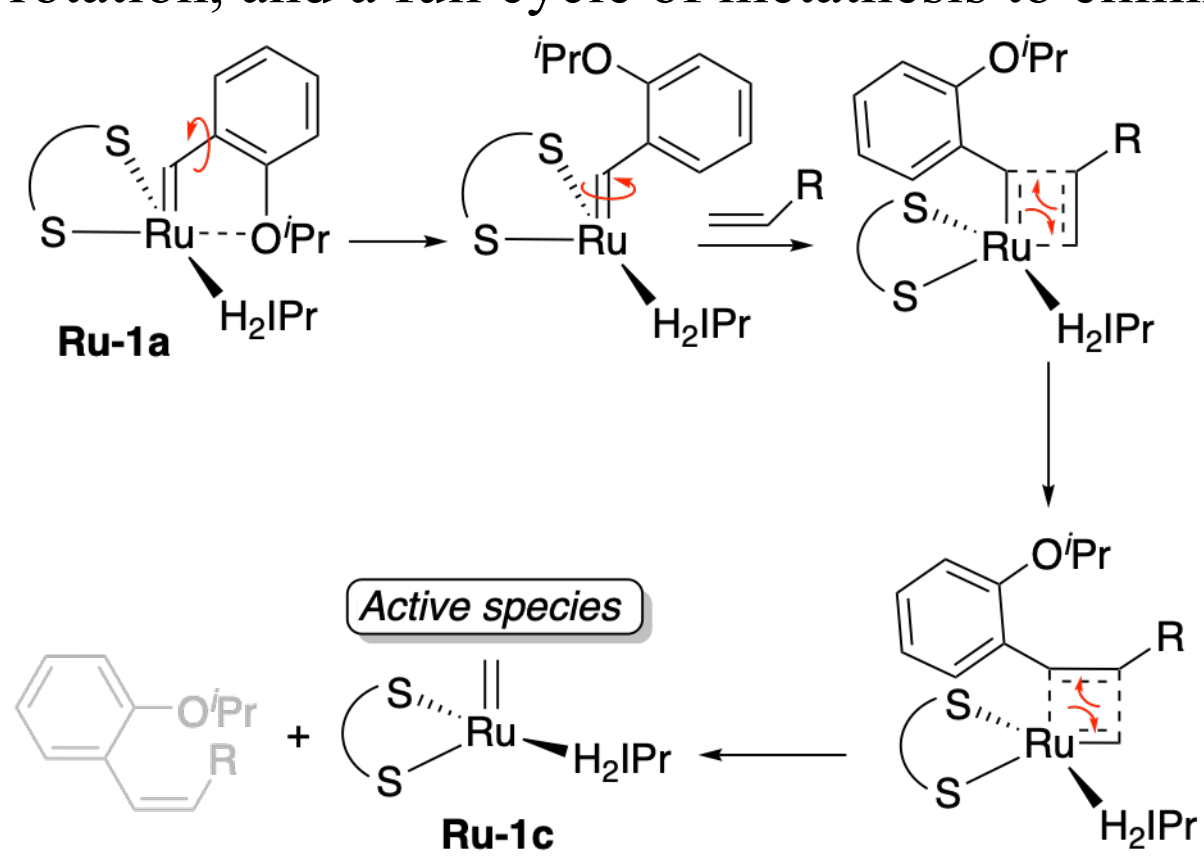

Scheme S1. Initiation of chelated catalyst **Ru-1a** to form **Ru-1c**.

S1.4.1 Self-Metathesis of Methyl Oleate (3). For data, see Table S2.

By Ru-1a. In a representative procedure, a solution of **3** (136 μL, 0.40 mmol) and dodecane (IS, 91 μL, 0.40 mmol, 1.0 equiv) in 720 μL THF in a 20 mL vial was stirred for ca. 2 min. An aliquot was removed, quenched with KTp[9] in THF (2 drops of a 10 mg/mL solution), and analyzed by GC to establish the initial ratio of internal standard vs **3**. Catalyst **Ru-1a** (51 μL of a stock solution of 10 mg in 3 mL THF, 0.0002 mmol, 0.0005 equiv) was injected to the substrate solution. The reaction was permitted to stir for 4 h, then analyzed.

Simulating incomplete air-exclusion: As above, but dissolving solid **Ru-1a** in dry THF on the bench (ca. 1 min air-exposure) before transferring to the glovebox for catalysis.

By in situ-Generated Ru-1b. A solution of **3** was made up and sampled as above. To generate **Ru-1b**, a solution of ylide **1b** (26 μL, 0.0004 mmol, 0.001 equiv; prepared as described in S1.3.5 and stirred for 15 min) was added to a vial containing **Ru-2** (29 μL of a 5 mg/mL stock solution

in THF, 0.0002 mmol, 0.0005 equiv) in 318 µL THF. After stirring for 10 min to form **Ru-1b**, the solution of **3** was added. The reaction was permitted to stir for 4 h, then analyzed.

Table S2. Results for Self-Metathesis of Methyl Oleate **3**.[a]

Trial	Catalyst	Yield **4** (%)	Yield **5** (%)	Total Yield (%)	TON
1	**Ru-1a**	23	22	46	910
2	**Ru-1a**	23	22	46	910
1 + air	[b]**Ru-1a**	13	13	26	512
2 + air	[b]**Ru-1a**	13	12	25	504
1	**Ru-2** + **1b**	24	23	47	932
2	**Ru-2** + **1b**	23	22	44	902
1	**Ru-2** + **1c**	23	22	45	906
2	**Ru-2** + **1c**	23	22	45	904

[a]Conditions: THF, RT, 400 mM **3**, 0.05 mol% Ru. Assessed at 4 h. [b]With incomplete air-exclusion.

Regenerating Ru-1b During Self-Metathesis of 3. Initially as above, but in a flask equipped with a side-arm in an oil bath at 30 °C. After removing an aliquot at 45 min to assess progress (see Table 3), the flask was removed from the glovebox and ca. 2 mL air was introduced into the headspace with a pipette. The flask was resealed, shaken, and the now-green (originally yellow) solution was evacuated and refilled with N_2 (4×). An aliquot was removed at t = 75 min, and **1b** was injected (53 µL of a 0.015 M solution in THF, 0.0008 mmol, 4 equiv vs Ru). Air-exposure, ylide addition, and sampling were repeated after 45 min, then 25 min. As little metathesis occurred in the final cycle, 4 equiv **1b** was added at 280 min, and monitoring continued to 21 h.

Table S3. Regenerating **Ru-1b** with Ylide **1b** in Self-Metathesis of **3**

Time (min)	Yield **4** (%)	Yield **5** (%)	Total Yield (%)
0	0	0	0
45	7	7	14
75	7	7	14
120	12	12	24
165	13	12	24
190	16	14	30
220	15	14	30
245	16	15	31
278	–	–	31*
300	19	17	36
345	21	20	41
1260	24	23	47

*Yield based on plateau from 220 to 245 min.

S1.4.2 Procedure for mRCM of 6.

By Ru-1a. A mixture of diene **6** and the internal standard dodecane (in both cases 22 µL of a 0.34 M stock solution in benzene; 0.0075 mmol) was diluted with 2.45 mL THF in a 20 mL vial. A sample was removed, quenched as in S1.4.1 above, and analyzed as above to establish the initial GC ratio of dodecane to **6**. **Ru-1a** (32 µL of a stock solution of 10 mg in 1 mL THF; 0.00038 mmol, 0.05 equiv) was injected. The reaction was stirred at 45 °C and analyzed after 1 h.

By in situ-Generated Ru-1b. A solution of **6** was made up and sampled as above. In situ-

generated **Ru-1b**, prepared as in **S1.4.1** and allowed to react for 10 min, was added by pipette. The reaction was stirred at 45 °C for 1 h, then quenched as above and analyzed.

S1.4.3 ROMP of cis-Cyclooctene 8. Data are collected in Table S4.

By Ru-1a. Adapted from the reported procedure.[13] A solution of **Ru-1a** (283 µL of a stock solution of 10 mg/mL in THF, 0.0033 mmol) was diluted with 87 µL THF in a 4 mL vial equipped with a stirbar. Cis-cyclooctene **8** (130 µL, 1.0 mmol, 300 equiv) and ca. 10 mg TMB (internal standard) were added to a separate GC vial and shaken to mix. The stirred initiator solution was heated to 40 °C in a degassed oil-bath in the glovebox, **8** was added rapidly, and stirring was continued for 1 h. Ca. 200 µL ethyl vinyl ether was then added to quench ROMP. The polymer was precipitated by adding 1 mL MeOH, washed with further MeOH, dissolved in CH_2Cl_2 and transferred to a pre-weighed vial. The CH_2Cl_2 was evaporated and the vial was dried overnight under vacuum. Yield: 58 mg (53%). ^{1}H and ^{13}C NMR analysis of **poly-8** were consistent with reported values.[14,15] *E/Z* ratios were assessed by $^{13}C\{^1H\}$ NMR analysis using a 90 sec relaxation delay (Fig. S11).

<u>With brief air-exposure:</u> A vial containing **8** (130 µL, 1.0 mmol, 300 equiv) and ca. 6 mg TMB, was prepared in the glovebox as above. A solution of **Ru-1a** (189 µL of a 15 mg/mL stock solution, 0.0033 mmol) was taken up in a gas-tight syringe, the needle tip was protected with a septum, and both vial and syringe were removed from the glovebox. The vial was opened for ca. 30 sec, the initiator solution rapidly injected, and the vial immediately sealed and stirred at 40 °C for 1 h. ^{1}H NMR analysis indicated ca. 6% conversion of cyclooctene.

By in situ-Generated Ru-1b. **Ru-1b** was prepared in situ as in **S1.4.1** in a 4 mL vial (allowed to react for 10 min). A vial containing **8** (130 µL, 1.0 mmol, 300 equiv) and ca. 6 mg TMB, was prepared in the glovebox as above. The stirred initiator solution was heated to 40 °C in a degassed oil-bath in the glovebox, the solution of **8** was added rapidly, and stirring was continued for 1 h, after which ethyl vinyl ether (200 µL) was added to quench ROMP. The polymer was precipitated by adding 1 mL MeOH, washed with further MeOH, dissolved in CH_2Cl_2 and transferred to a pre-weighed vial. The CH_2Cl_2 was evaporated and the vial was dried overnight under vacuum. Yield: 66 mg (60%). ^{1}H and ^{13}C NMR of the polycyclooctene **poly-8** are consistent with the reported literature values.[14,15] The E/*Z* ratio was assessed by $^{13}C\{^1H\}$ NMR spectroscopy using a 90-sec relaxation delay (Figure S11, Table S4).

<u>With brief air-exposure:</u> Neat **8** (130 µL, 1.0 mmol, 300 equiv), ca. 6 mg TMB, and **Ru-2** (184 µL of a 13 mg/mL, 0.0033 mmol) were added to a vial equipped with a stirbar in the glovebox. Ylide **1b** was generated as described above, and the an appropriate volume (181 µL of a 0.092 M stock solution in THF, 0.017 mmol, 5 equiv) was taken up in a gas-tight syringe sealed with a septum. The vial and syringe were rapidly removed from the glovebox to the fumehood where the vial was heated to 40 °C in an oil bath for ca. 30 sec. The syringe was then uncapped, the vial was opened, and the ylide solution was rapidly injected. The vial was sealed immediately. The reaction was allowed to stir for 1 h before being assessed by ^{1}H NMR analysis. The resulting polymer was worked up and analyzed as above (Figure S11, Table S4).

<u>Control experiment with **Ru-2**</u>. A control experiment was carried out as described above, without added **1b**. No reaction was observed at 1 h, confirming that the oxo complex **Ru-2** itself is inactive in ROMP.

Table S4. Initiator performance in ROMP of cyclooctene **8**; average of 2 trials.

initiator	conv.	in situ yield (%; NMR)	isolated yield (%)	%*Z*	M_n[a]	M_W[a]	*Đ*
**Ru-1a**	100	–	53	90	48.3	80.7	1.67
**Ru-2** + **1b**	100	–	60	96	42.6	72.4	1.71
**Ru-1a**[b]	6	5	–	–	–	–	–
**Ru-2** + **1b**[c]	100	–	82	78	59.5	109	1.83
**Ru-2**[d]	0	0	–	–	–	–	–

[a]M_n(exp) and M_w(exp) in kg/mol; by GPC in THF vs polystyrene standards. Theoretical M_n is 33.1 kg/mol. [b]Conducted in N_2-filled vials outside the glovebox; opened to air to add **Ru-1a**. [c]In N_2-filled vials outside the glovebox; opened to air to add ylide **1b.** [d]In air.

S1.4.4 E/Z-Isomerization Inactivity of Ru-2. Outside glovebox. A solution of **4** (20 mg, 0.08 mmol, 100 equiv) in 1.0 mL dry THF was sampled to assess the initial *E/Z* ratio. **Ru-2** (130 µL of a 4.4 mg/mL stock solution in THF; 0.0008 mmol) was added. After stirring for 18 h, analysis as above confirmed no change.

S2. NMR Spectra Showing Characterization of New Compounds

S2.1 Ru(S-S)(PPh_3)(*p*-cymene), Ru-4.

(a) ^1H NMR spectrum (600 MHz, CD_2Cl_2).

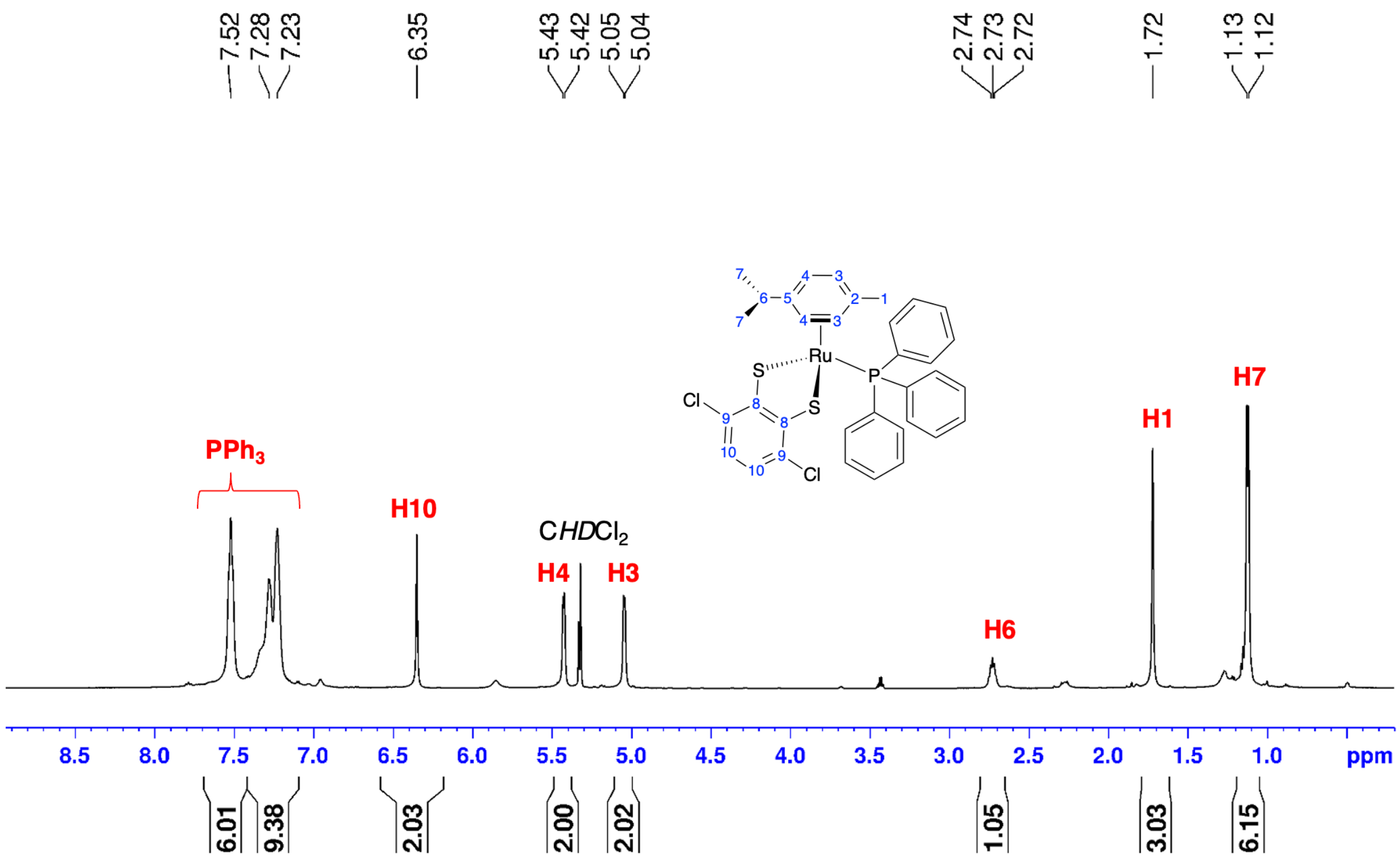

(b) $^{13}C\{^1H\}$ NMR spectrum (151 MHz, CD_2Cl_2).

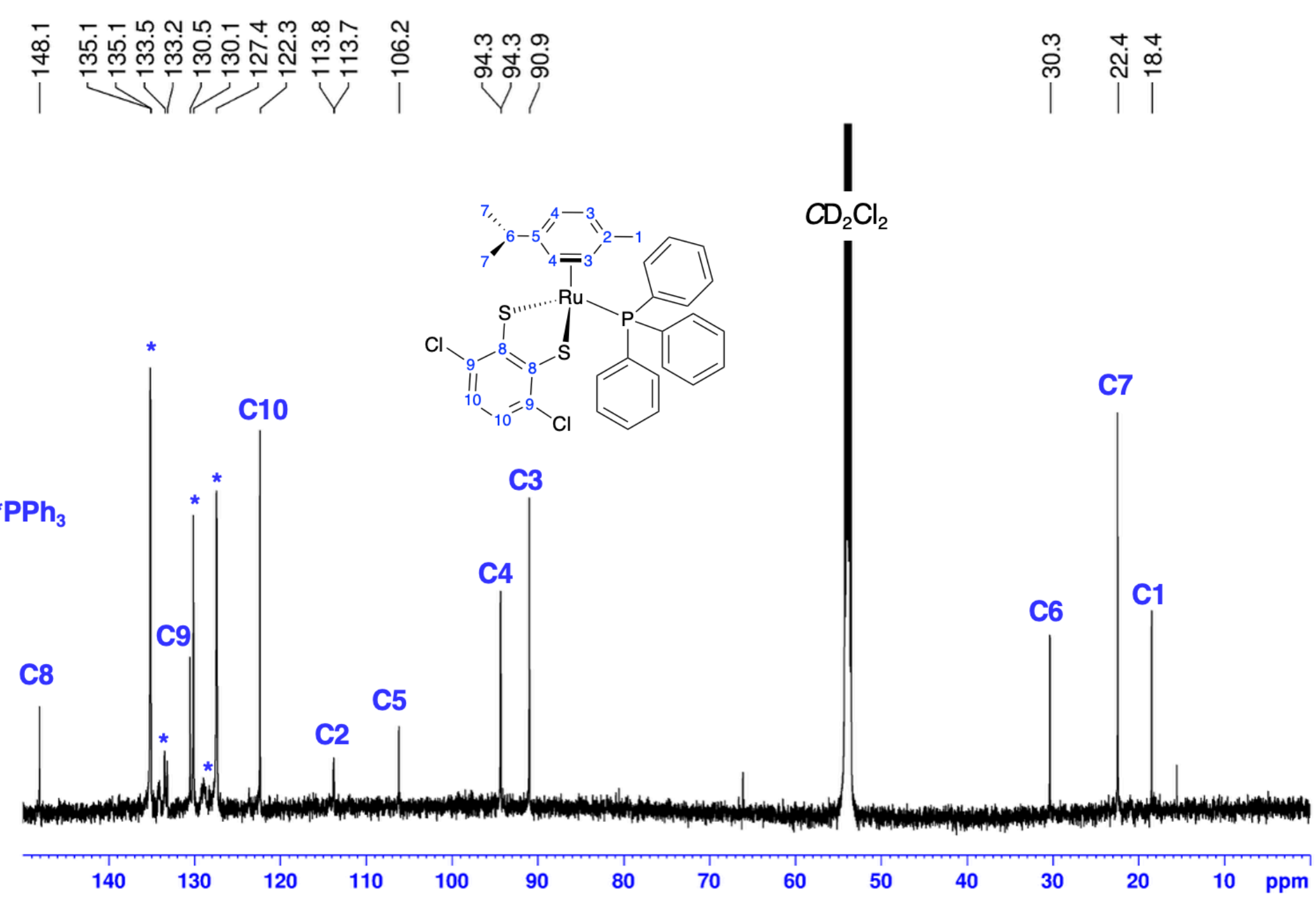

(continued next page)

(c) $^{31}P\{^{1}H\}$ NMR spectrum (202 MHz, CD_2Cl_2).

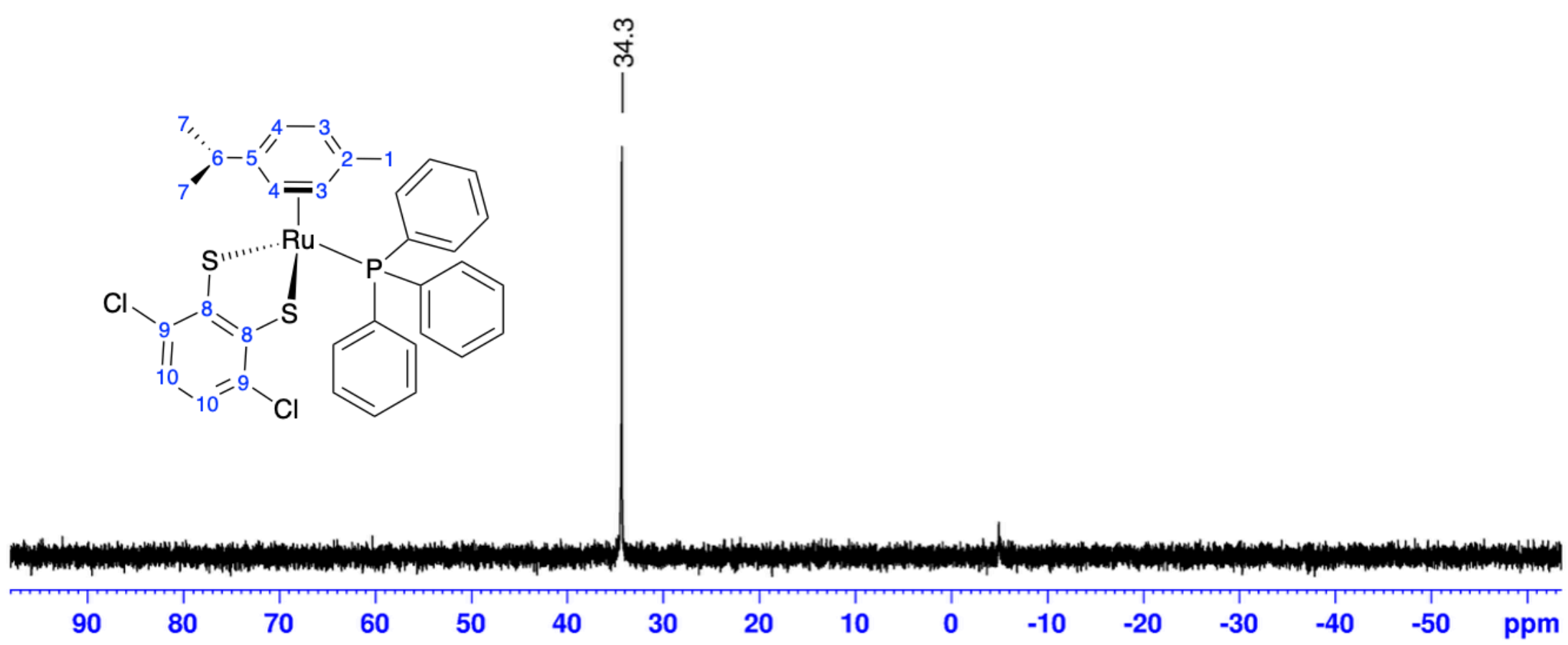

(d) ^{1}H-^{1}H COSY NMR spectrum (600 MHz, CD_2Cl_2).

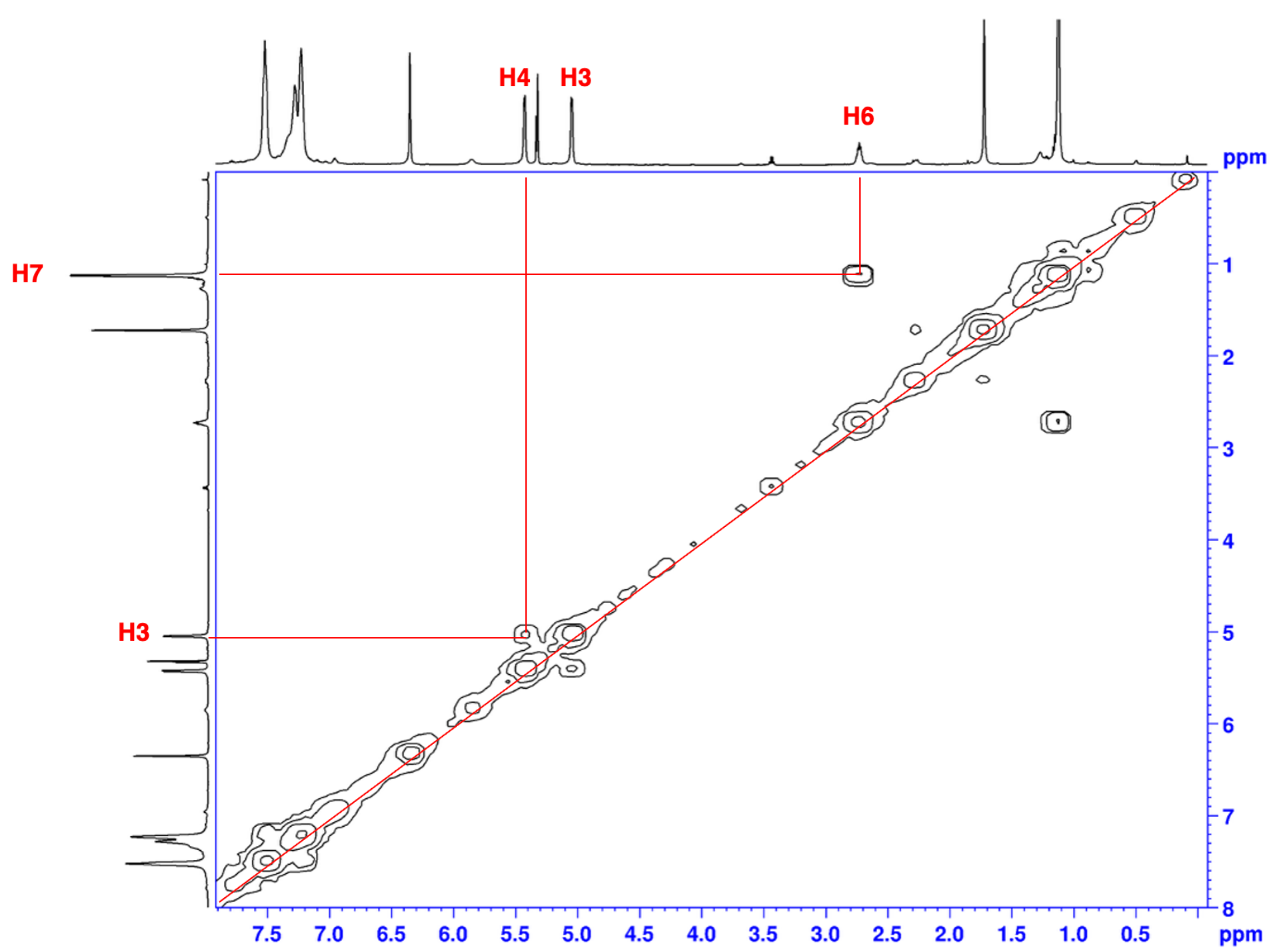

(continued next page)

(e) ^{1}H-^{13}C HSQC NMR spectra (600/150 MHz, CD_2Cl_2): expanded aromatic (top) and aliphatic (bottom) regions.

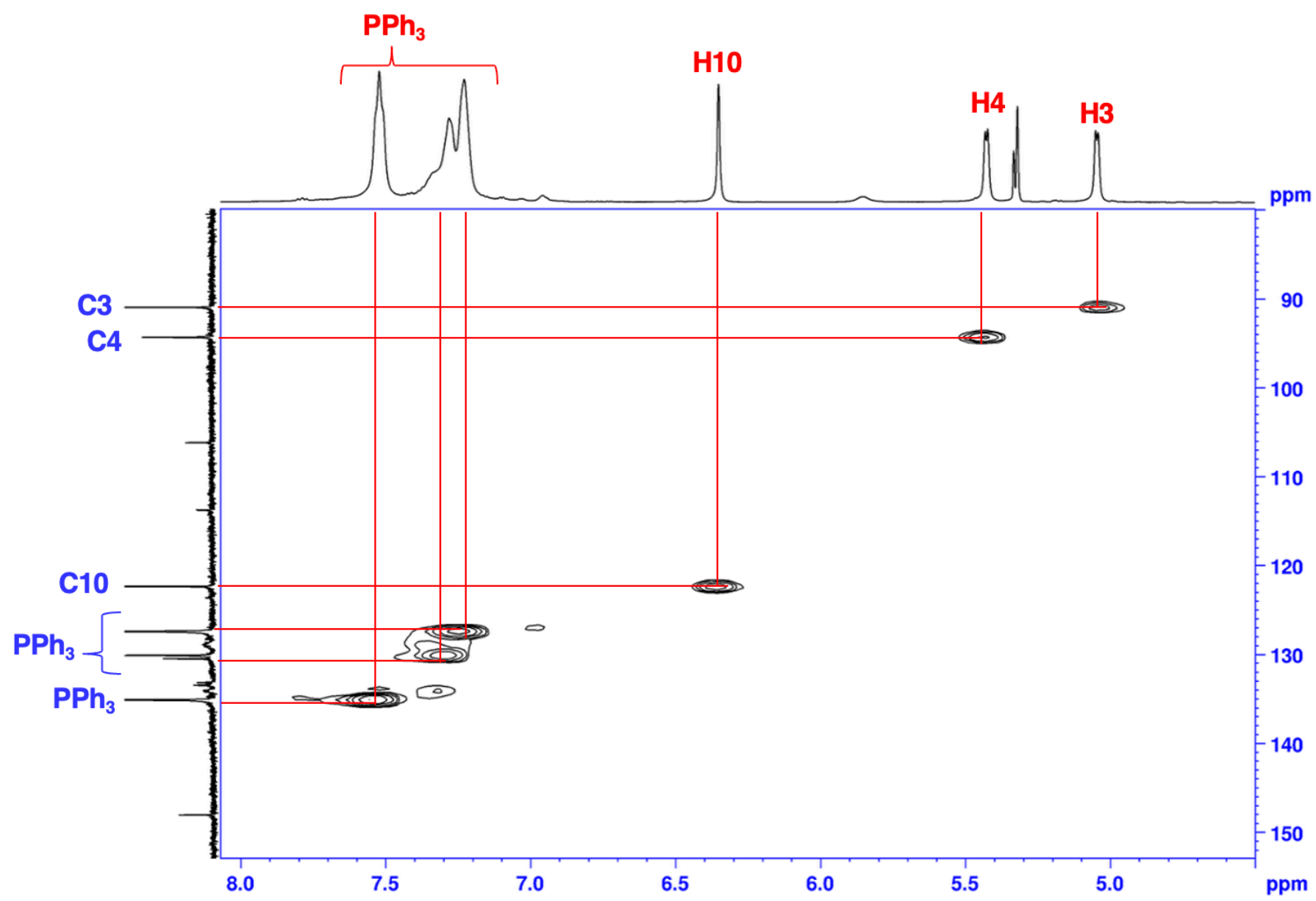

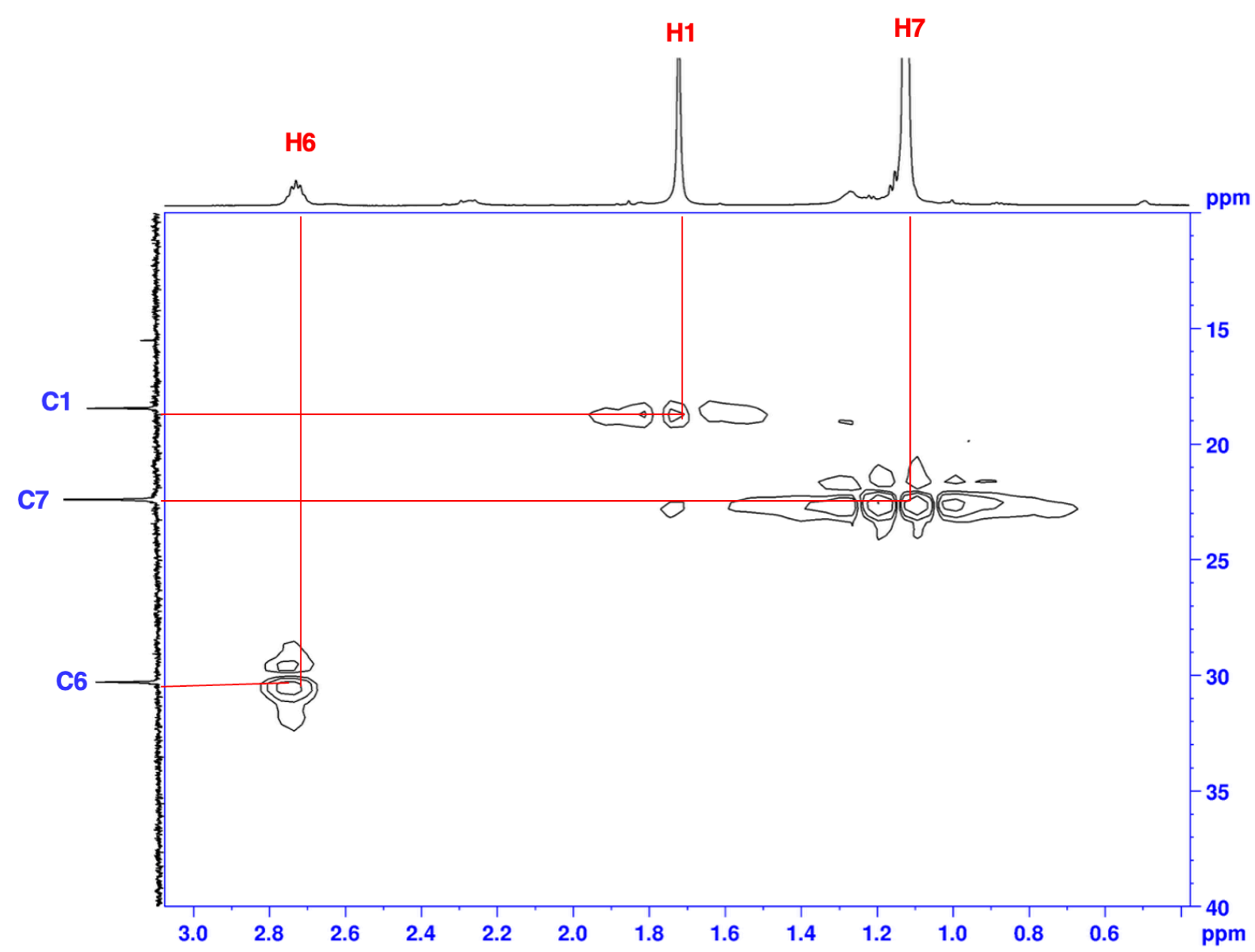

(continued next page)

(f) ^{1}H-^{13}C HMBC NMR spectra (600/150 MHz, CD_2Cl_2): expanded aromatic (top) and aliphatic (bottom) regions.

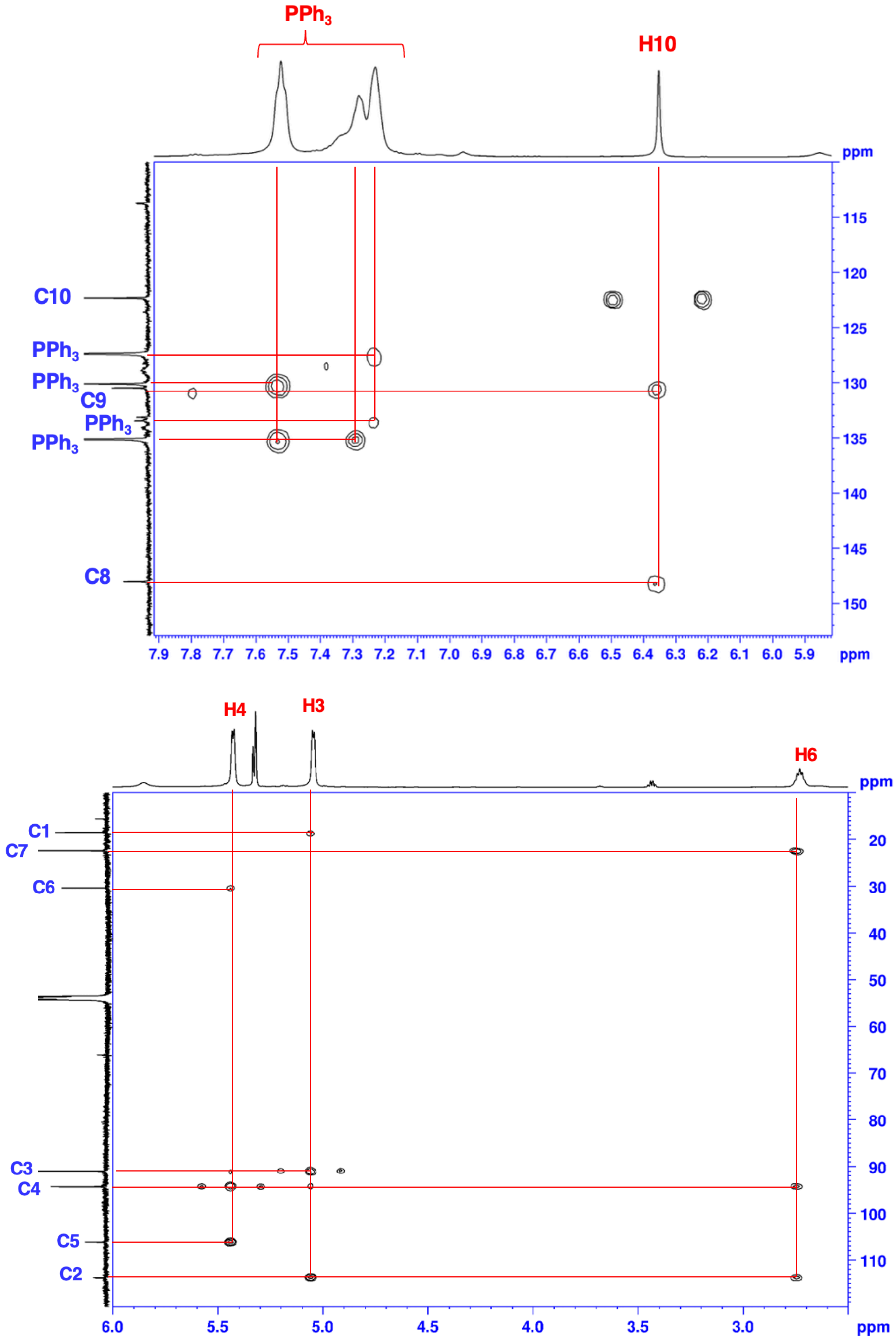

Figure S1. NMR characterization of Ru(S-S)(PPh$_3$)(*p*-cymene) (**Ru-4**) in CD_2Cl_2. (a) ^{1}H NMR (600 MHz). (b) ^{13}C{^{1}H} NMR (151 MHz). (c) ^{31}P{^{1}H} NMR (202 MHz). (d) ^{1}H-^{1}H COSY NMR (600 MHz). (e) ^{1}H-^{13}C HSQC NMR (600/150 MHz). (f) ^{1}H-^{13}C HMBC NMR (600 MHz, 150 MHz).

S2.2 Ru(S-S)(PPh_3)(H_2IPr), Ru-5.

(a) ^{1}H NMR (600 MHz, CD_2Cl_2).

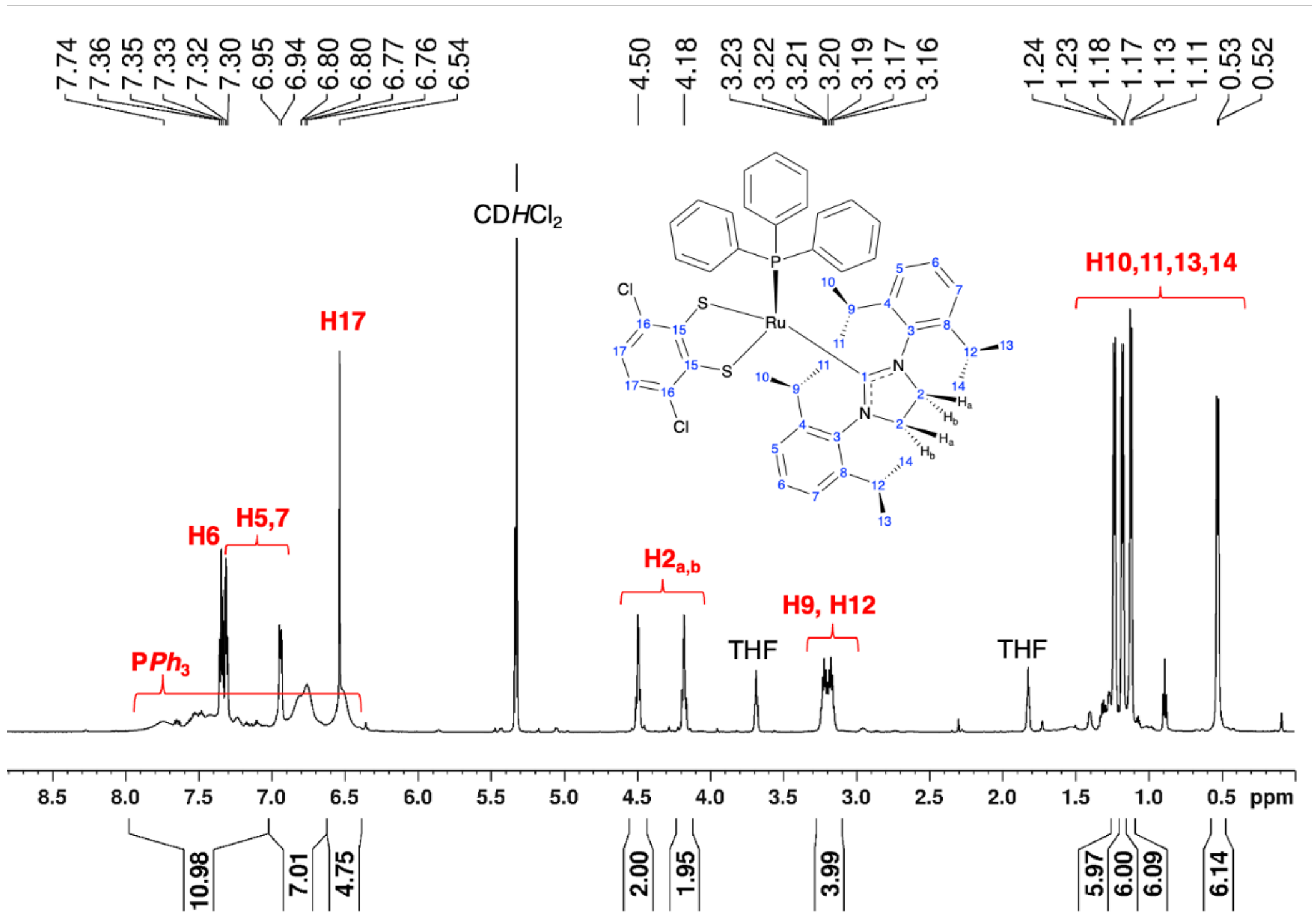

(b) $^{13}C\{^{1}H\}$ NMR (151 MHz, CD_2Cl_2).

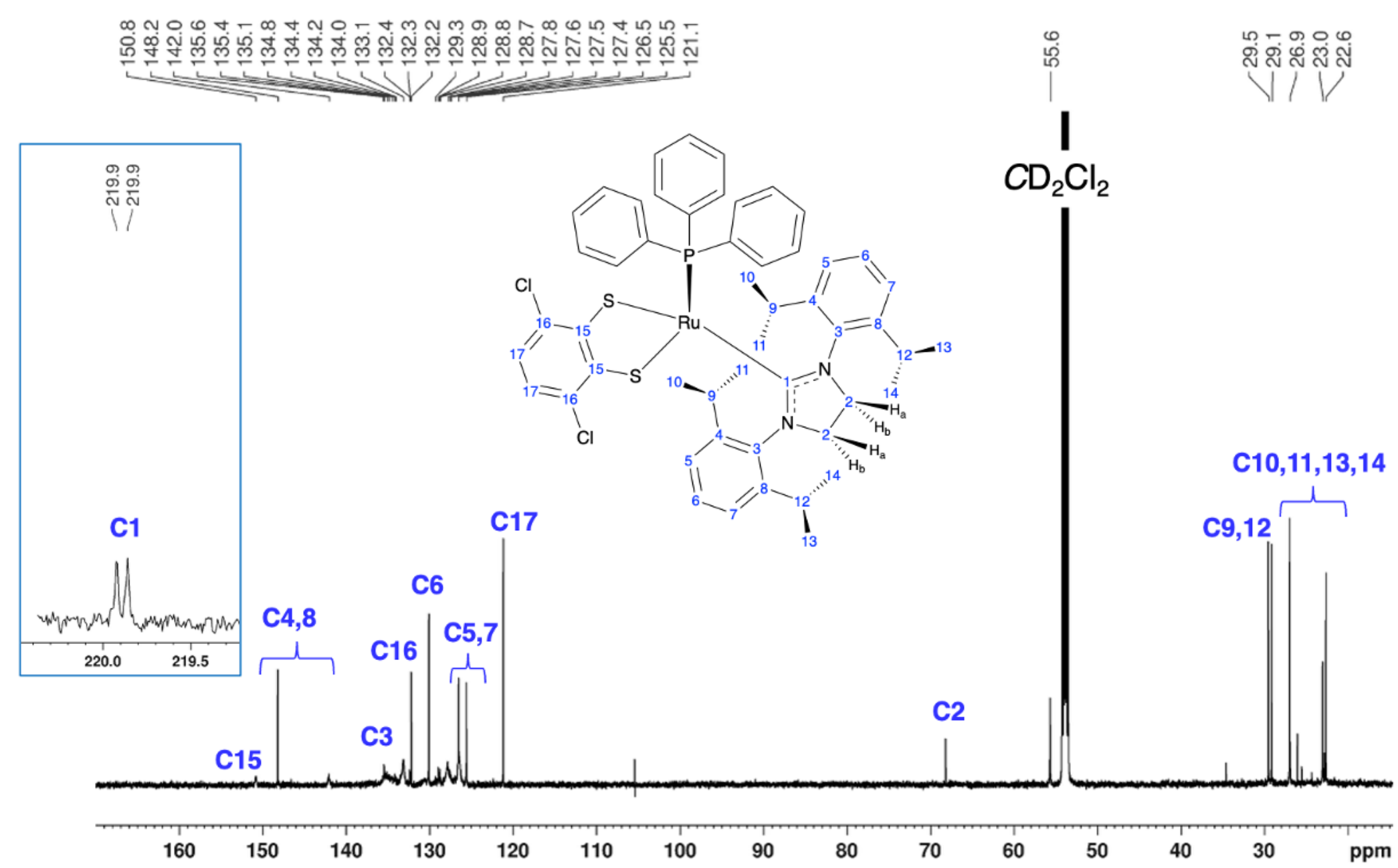

(continued next page)

(c) $^{31}P\{^{1}H\}$ NMR (202 MHz, CD_2Cl_2).

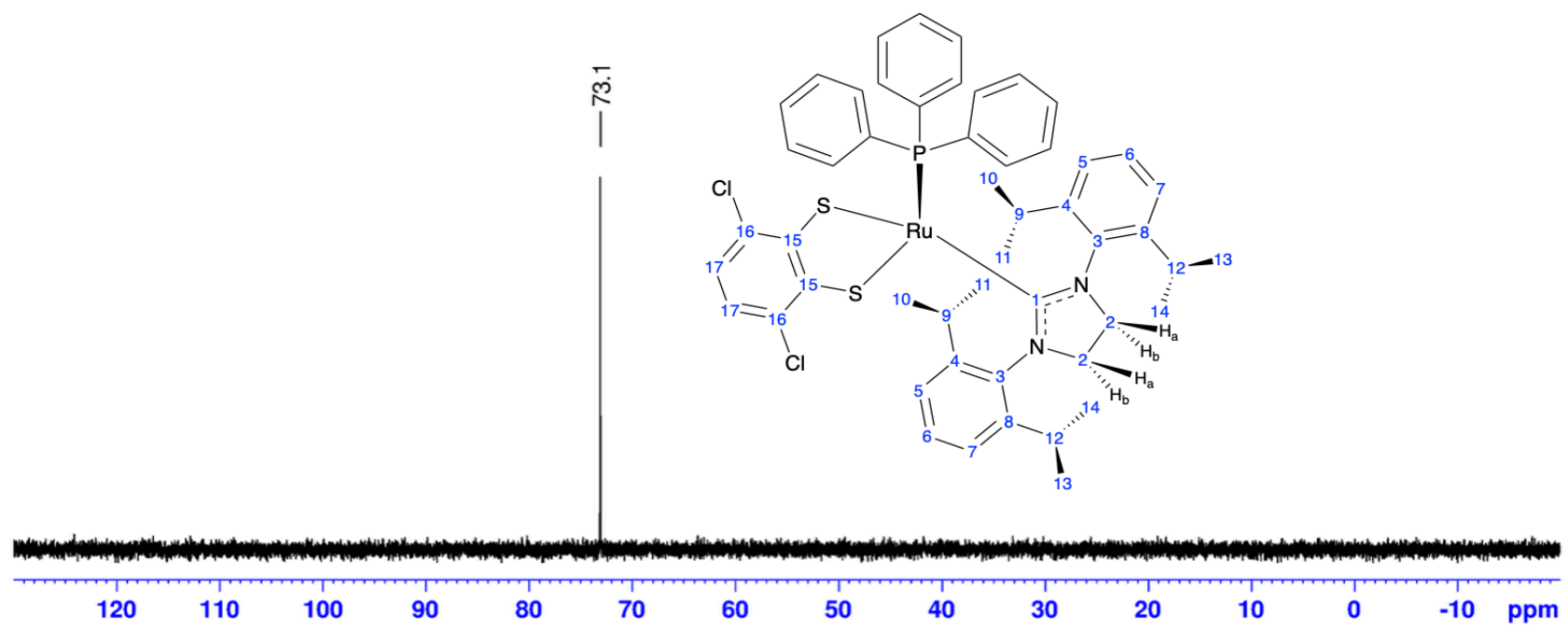

(d) ^{1}H-^{1}H COSY NMR (600 MHz, CD_2Cl_2).

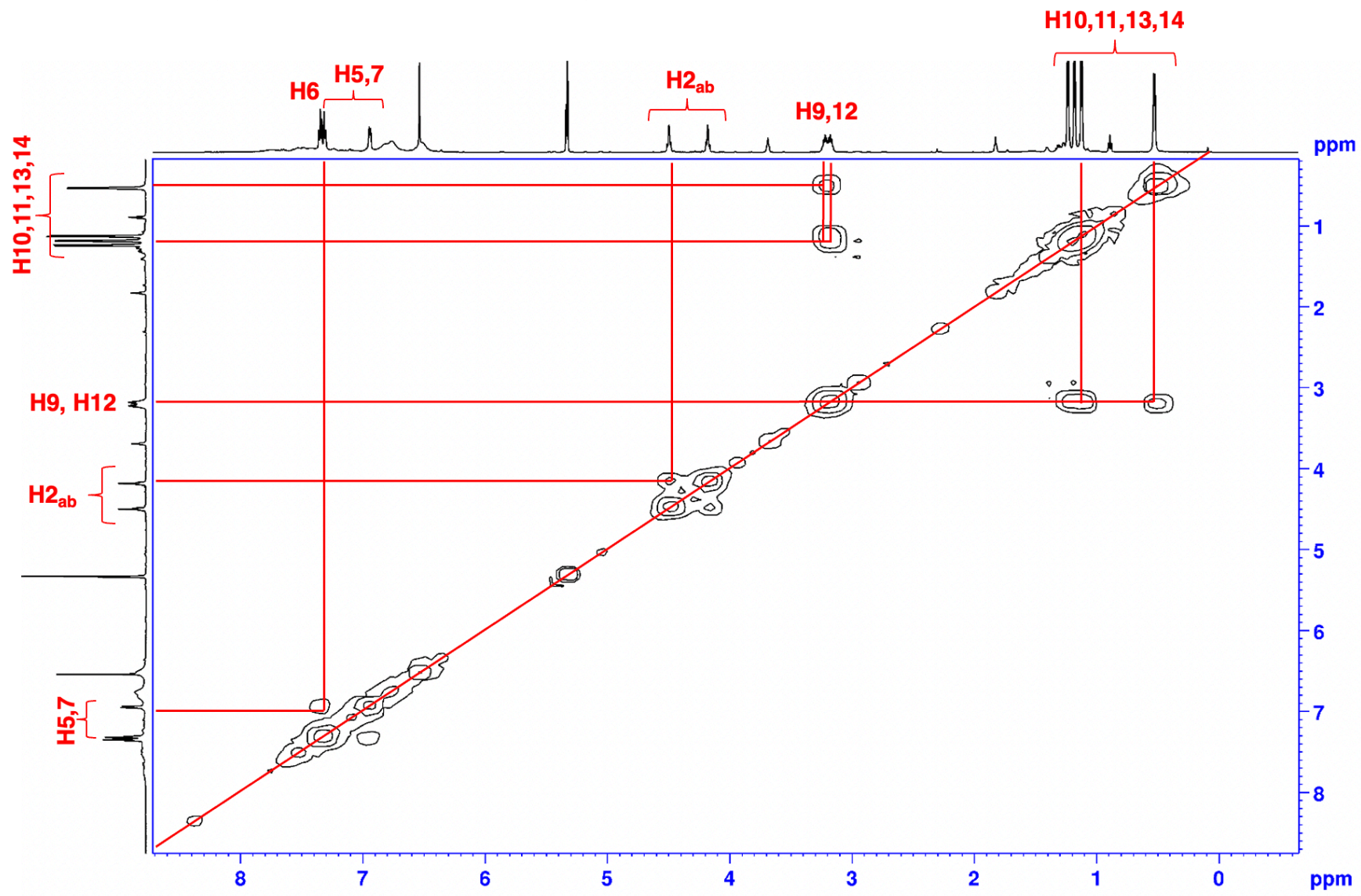

(e) ^{1}H-^{13}C HSQC NMR (600/150 MHz, CD_2Cl_2): expanded aliphatic region.

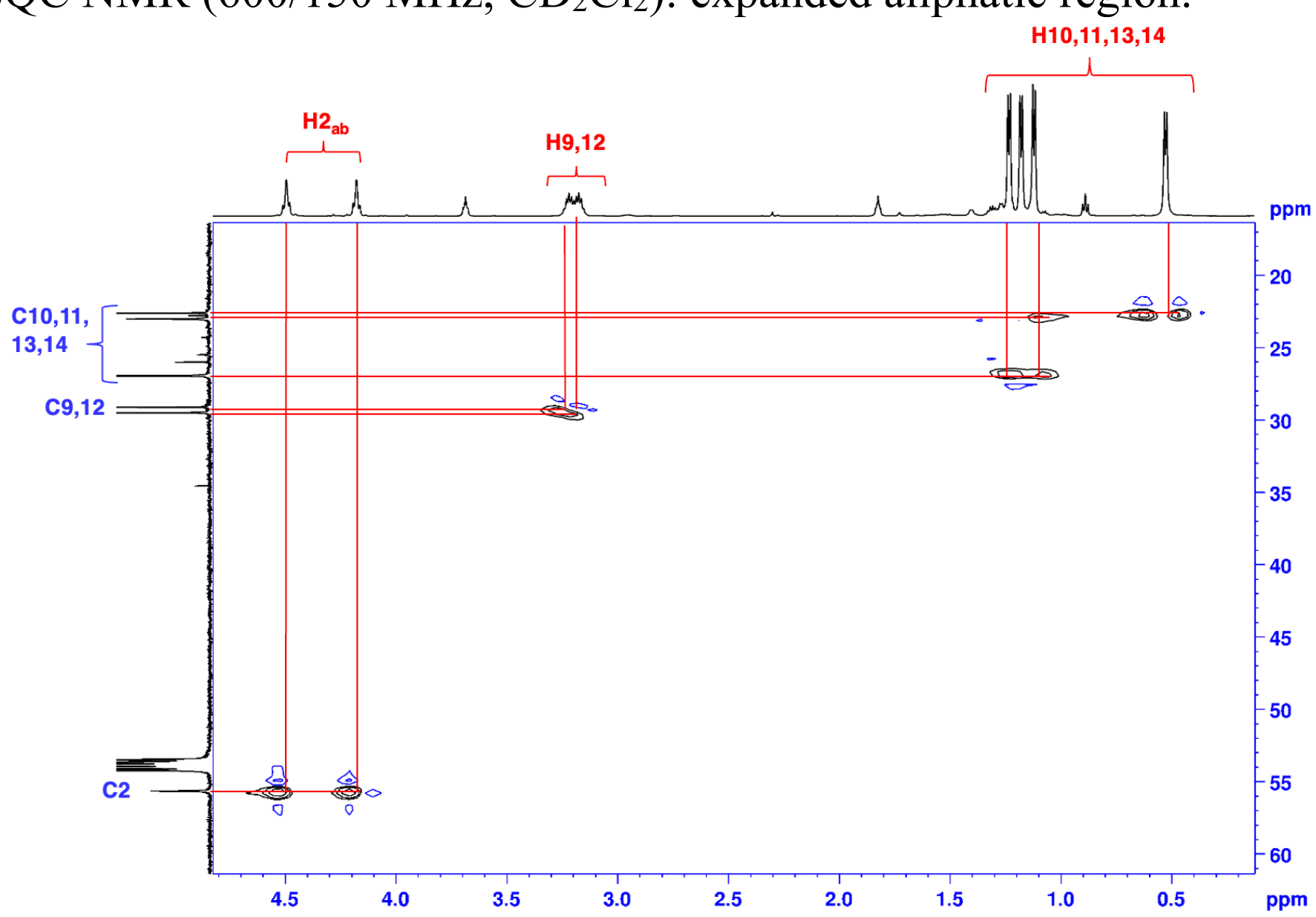

(continued next page)

(e) (cont'd) ^1H-^{13}C HSQC NMR (600/150 MHz, CD_2Cl_2): expanded aromatic region.

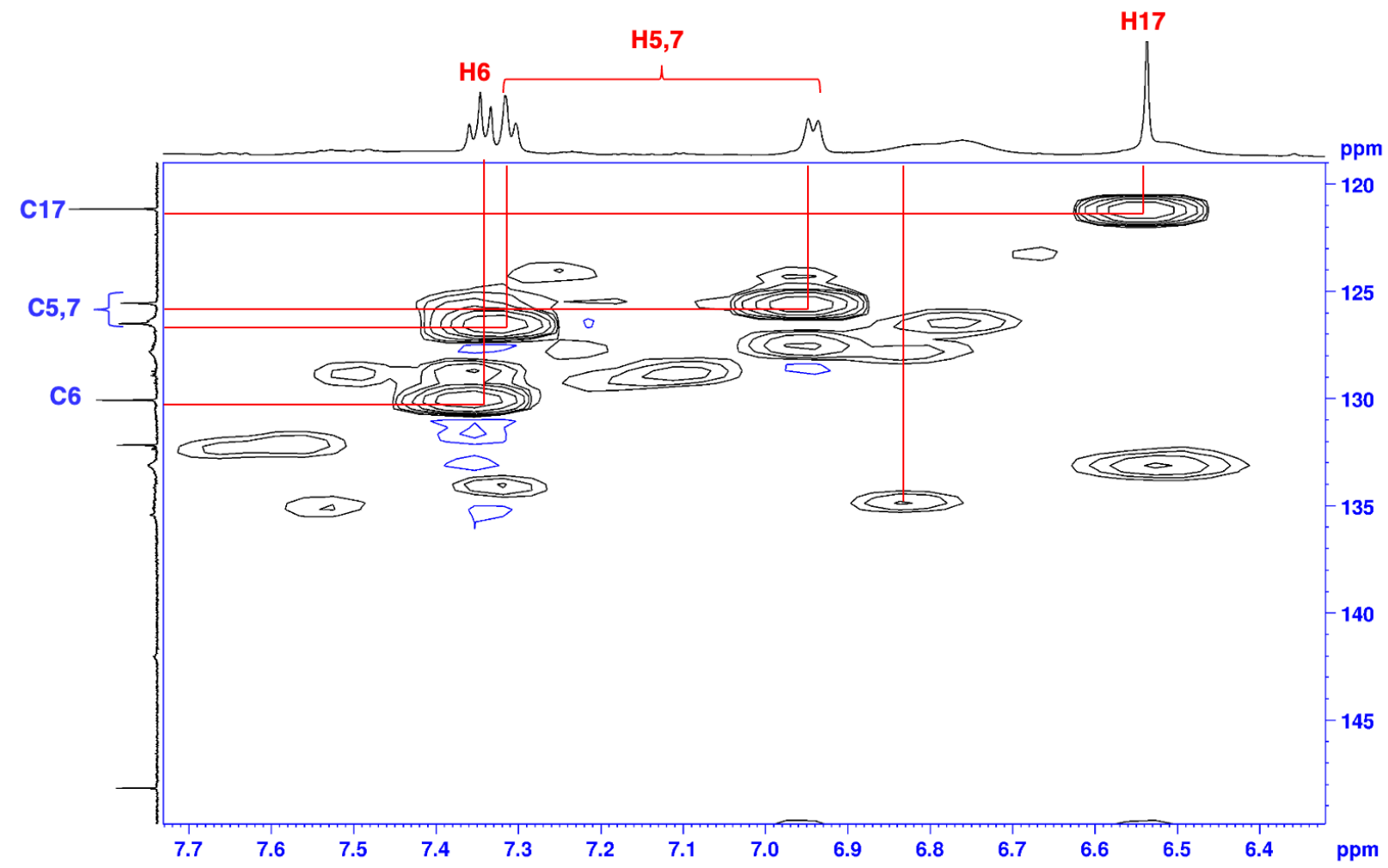

(f) ^1H-^{13}C HMBC NMR (600 MHz, 150 MHz, CD_2Cl_2): expanded aliphatic (top) and aromatic (bottom) regions.

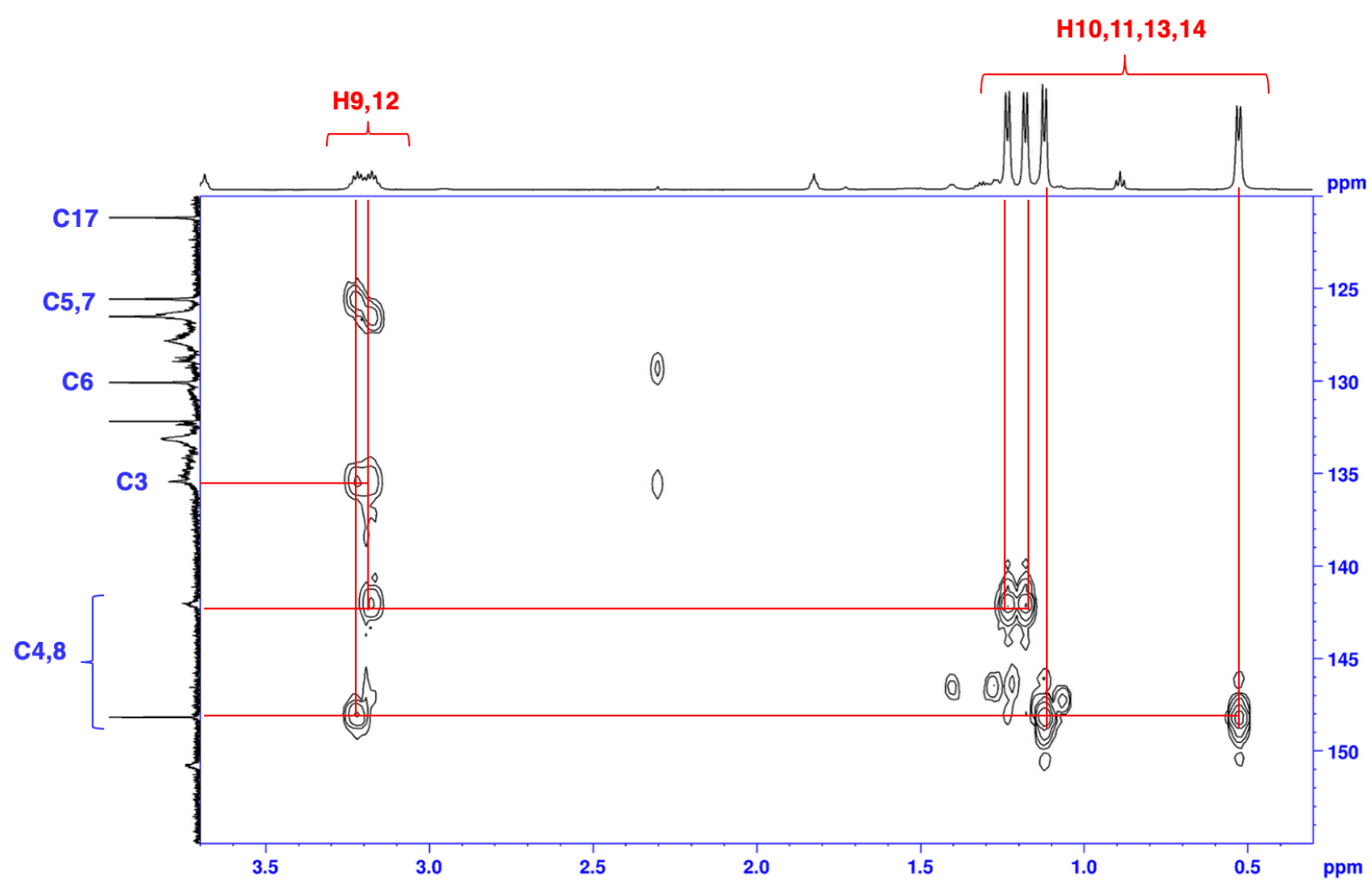

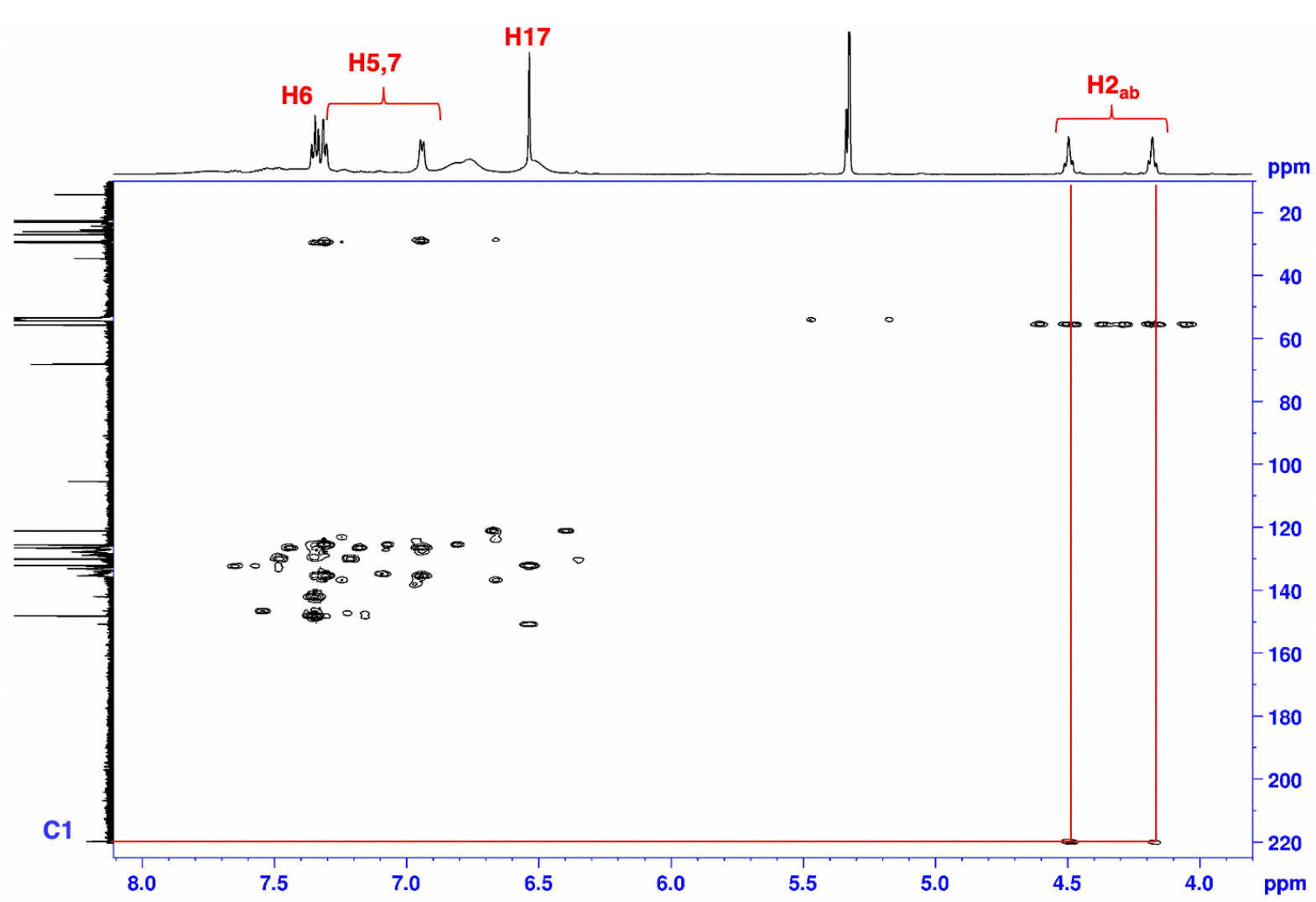

(continued next page)

(f) (cont'd) ^{1}H-^{13}C HMBC NMR (600 MHz, 150 MHz, CD_2Cl_2) showing expanded regions.

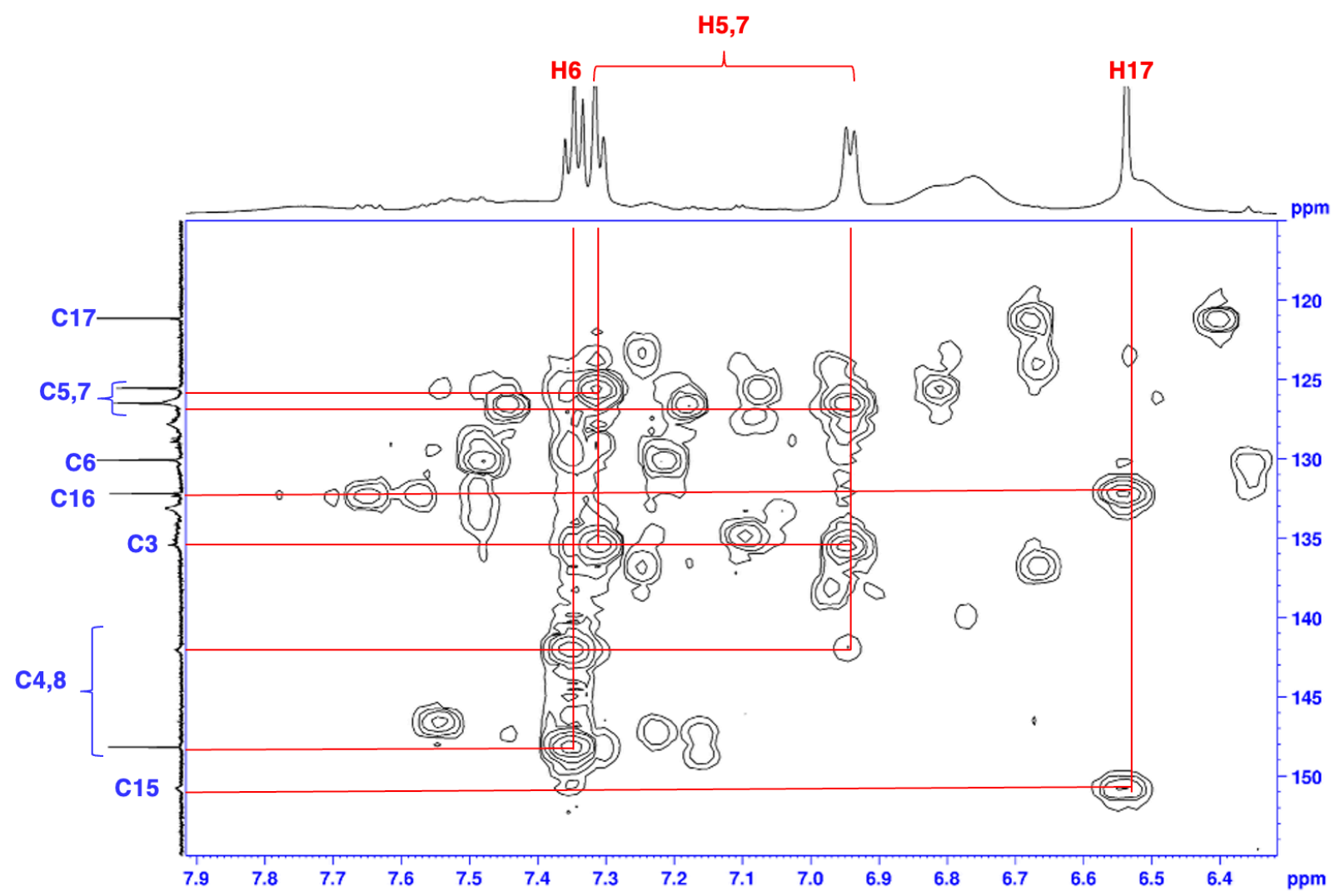

Figure S2. NMR spectra of **Ru-5** in CD_2Cl_2. (a) ^{1}H NMR (600 MHz). (b) ^{13}C{^{1}H} NMR (151 MHz). (c) ^{31}P{^{1}H} NMR (202 MHz). (d) ^{1}H-^{1}H COSY NMR (600 MHz). (e) ^{1}H-^{13}C HSQC NMR (600/150 MHz) showing expanded regions. (f) ^{1}H-^{13}C HMBC NMR (600 MHz, 150 MHz) showing expanded regions.

S3. Representative NMR Spectra and Chromatograms for Reactions

S3.1 Oxidation of Ru(S-S)(H_2IPr)(PPh_3) (Ru-5) by Air to Form Ru-2.

(a) t = 0 min

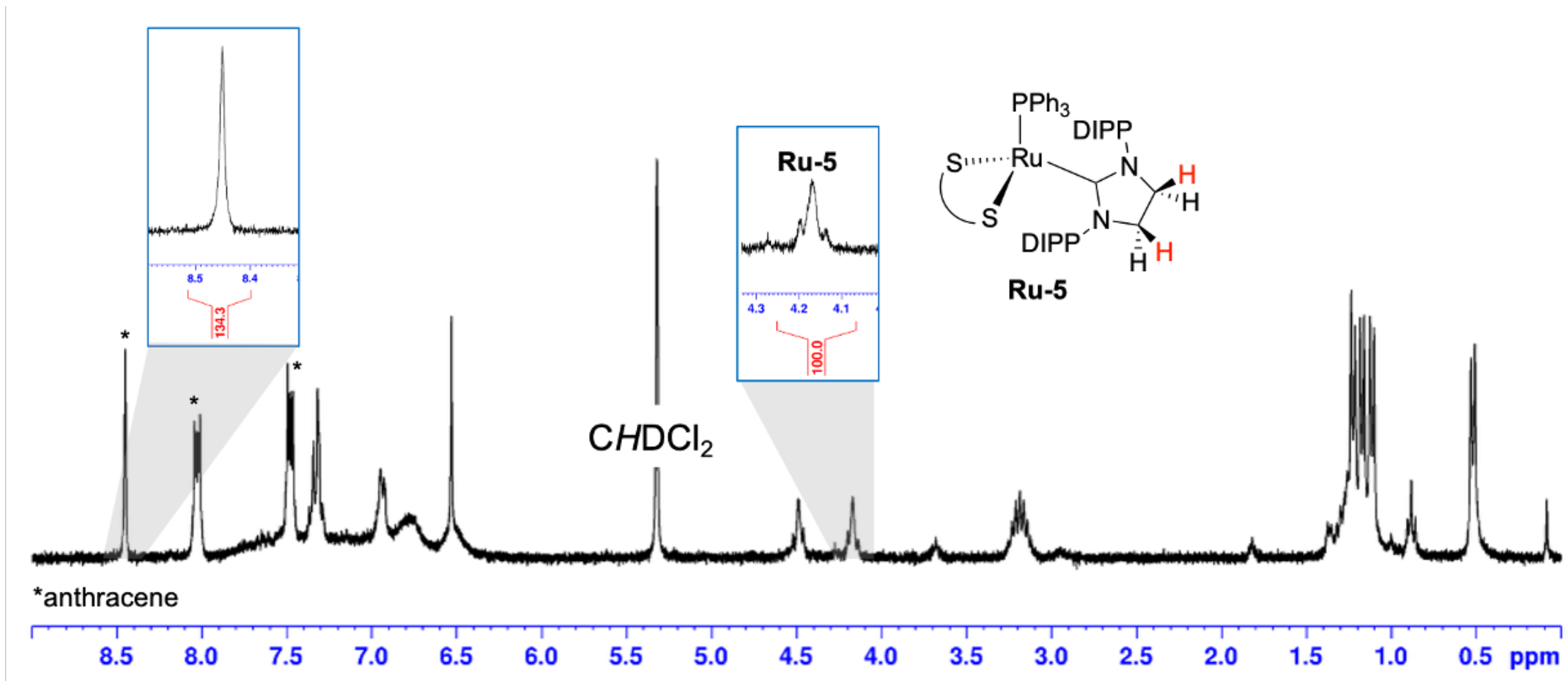

(a) t = 5 min

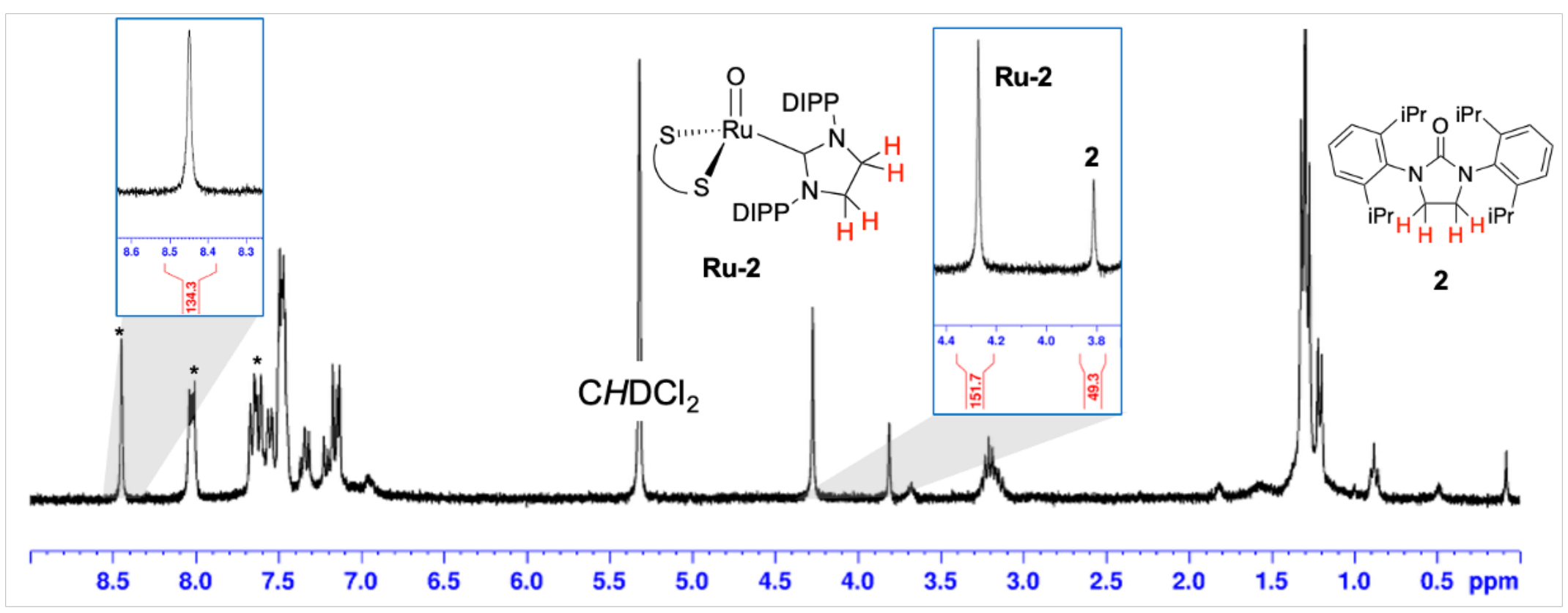

Figure S3. ^{1}H NMR spectra (300 MHz, CD_2Cl_2) showing oxidation of **Ru-5** by air to form **Ru-2** and urea **2**.

S3.2 Oxidation of $Ru(S\text{-}S)(H_2IPr)(PPh_3)$ (Ru-5) by 8% O_2 in Ar.

(a) t = 0 min

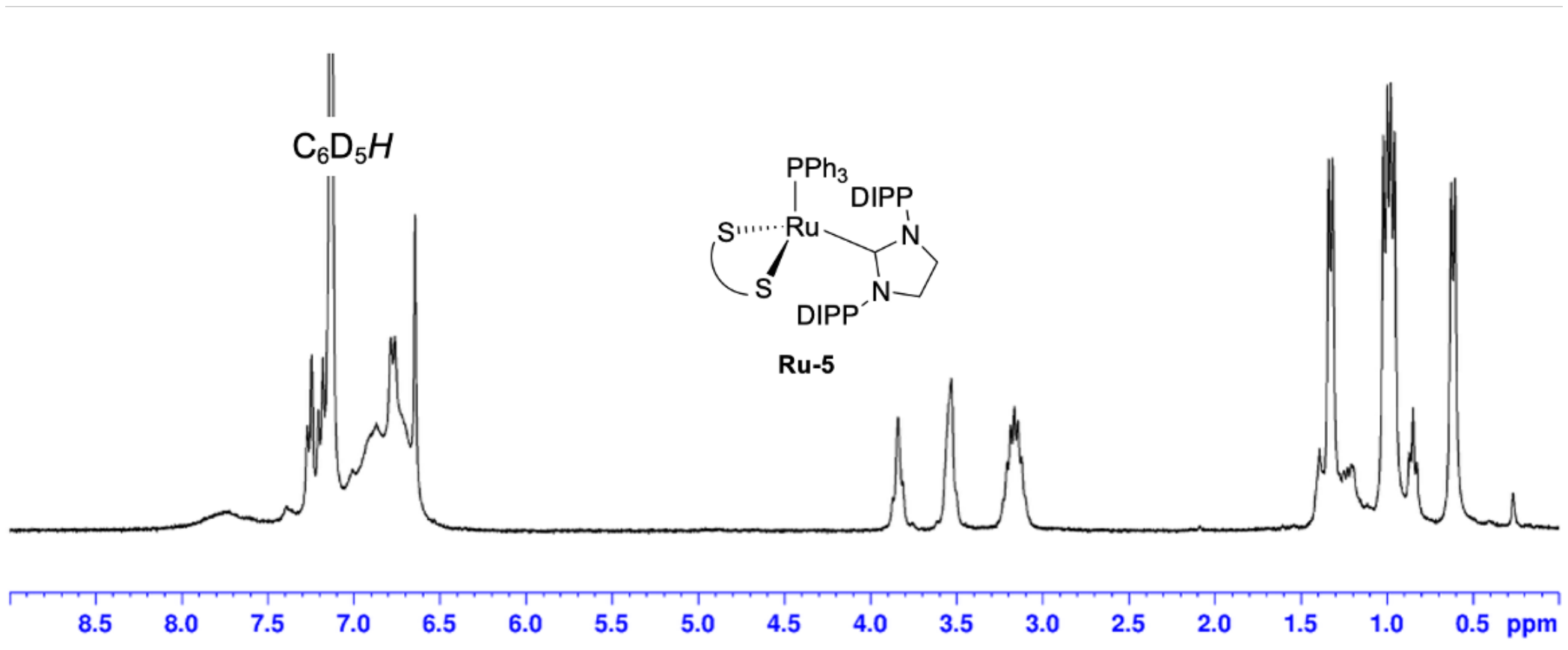

(b) t = 8 min

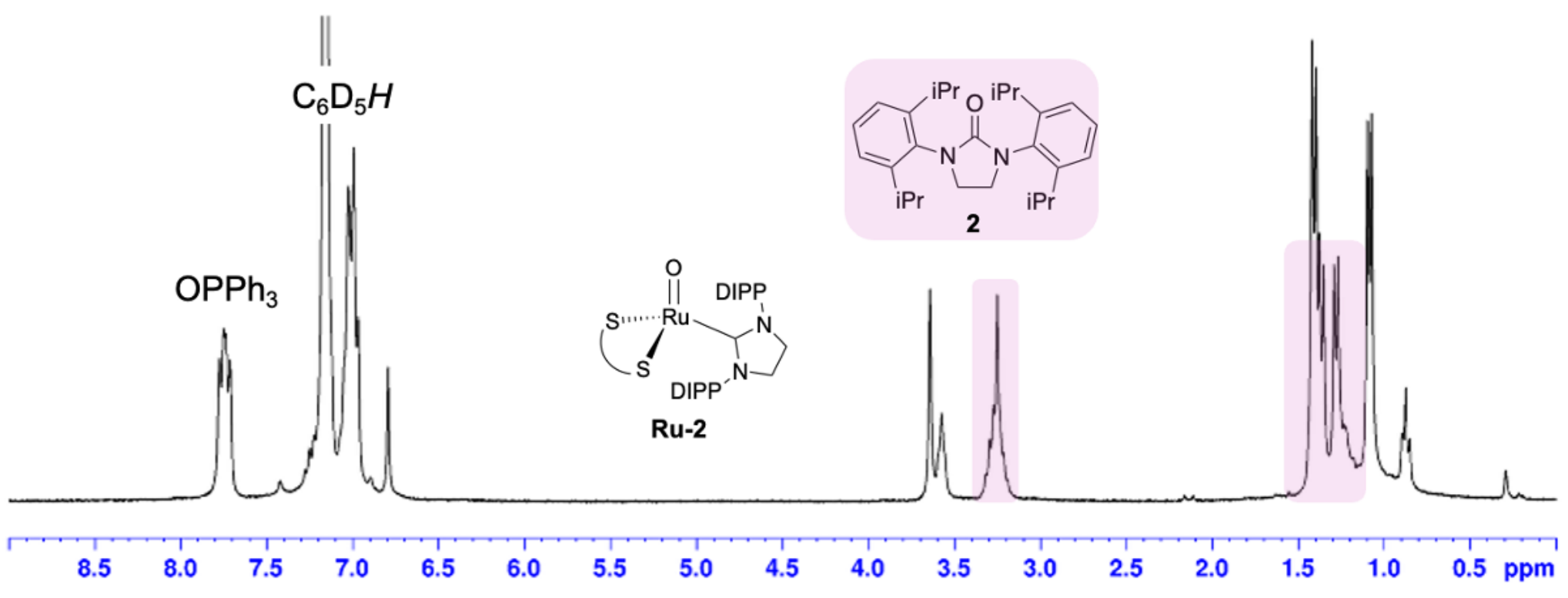

Figure S4. ^1H NMR spectra (300 MHz, C_6D_6) showing reaction of **Ru-5** with 8% O_2 in Ar, with formation of **Ru-2** and urea **2**.

S3.3 Reaction of Ru-2 with PPh_3 under N_2.

(a) ^1H NMR (300 MHz, C_6D_6).

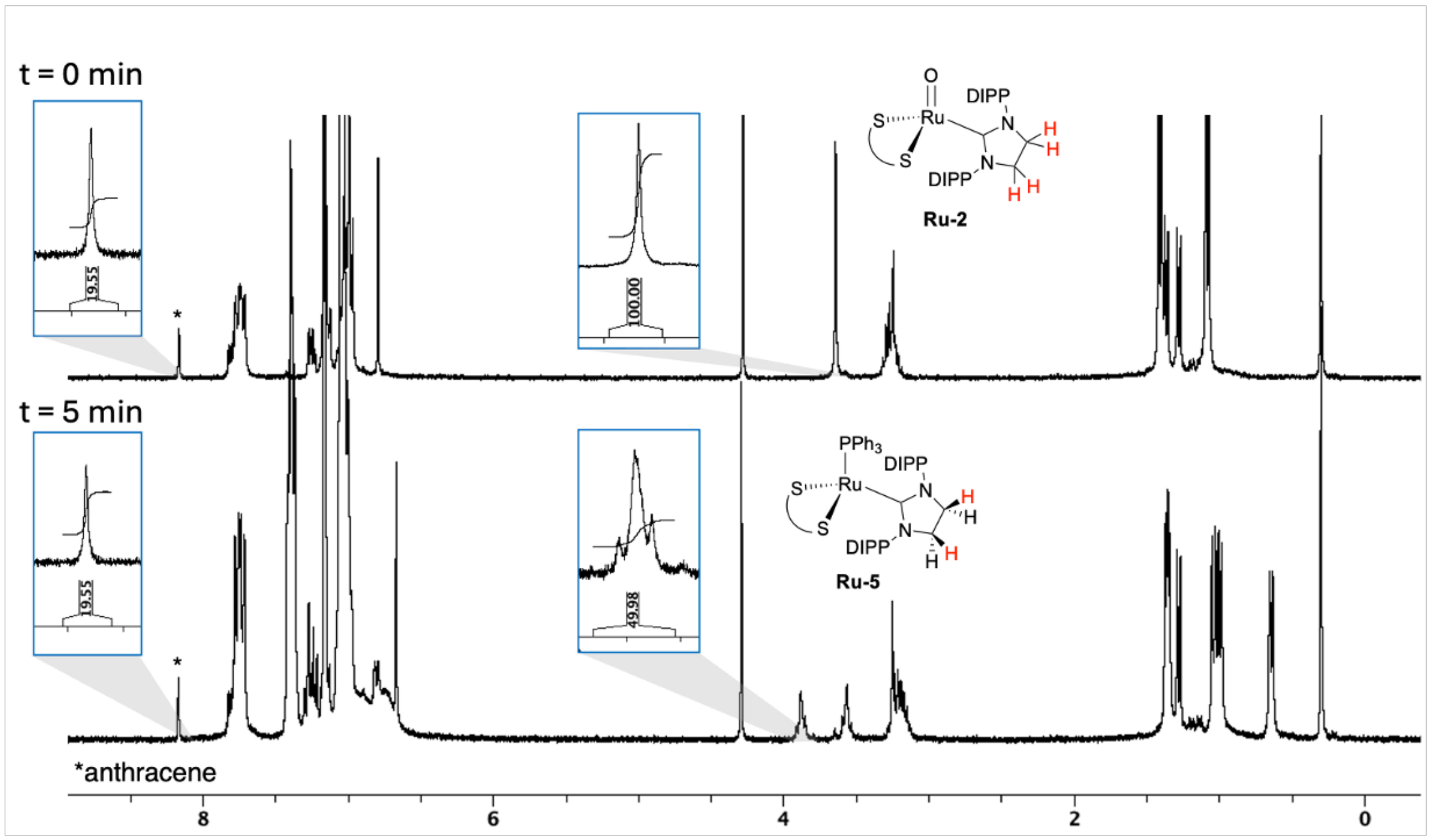

(b) $^{31}P\{^1H\}$ NMR (121 MHz, C_6D_6)

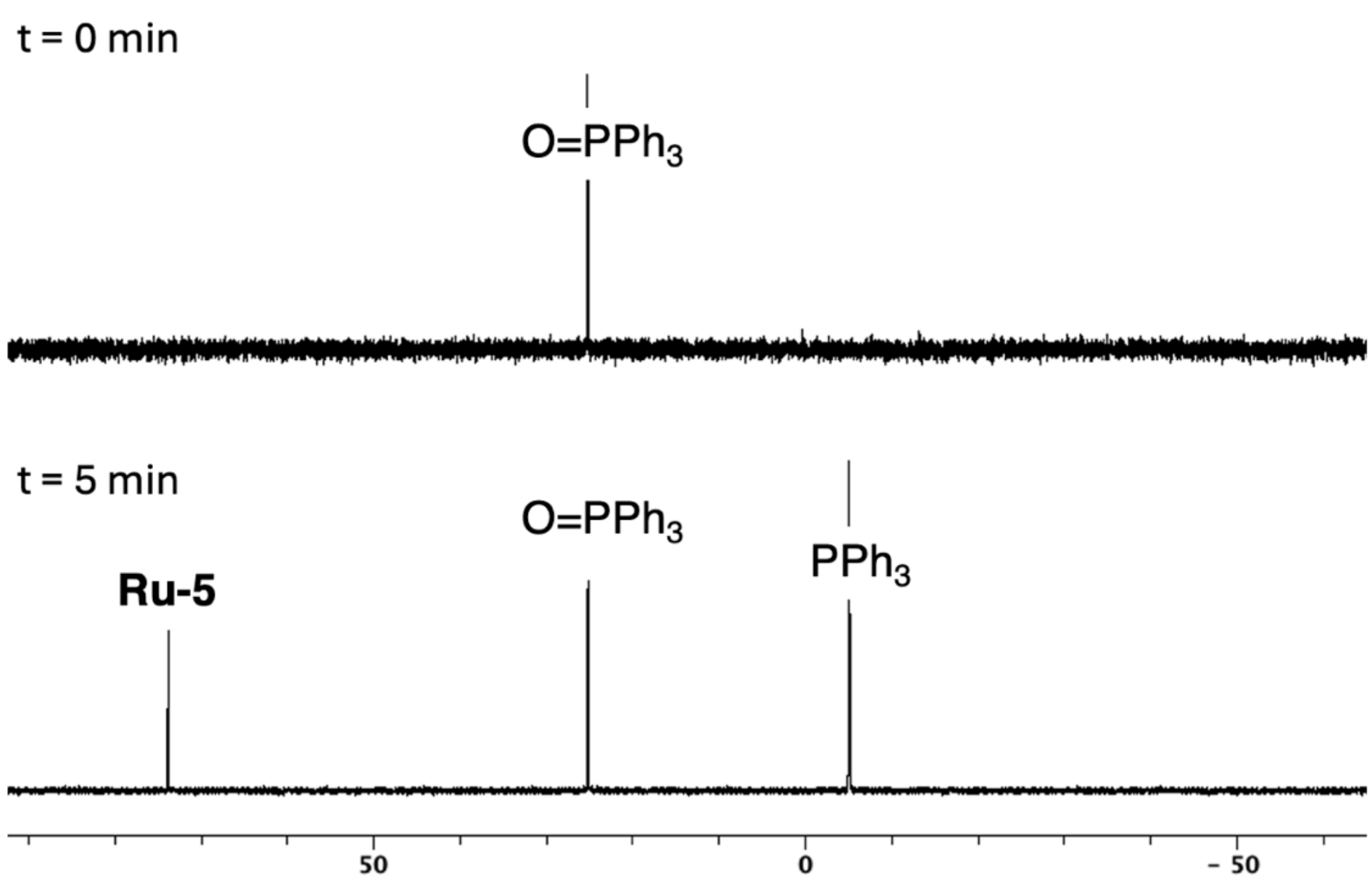

Figure S5. Conversion of oxo complex **Ru-2** to PPh_3 complex **Ru-5** under an atmosphere of N_2.

S3.4 NMR-Scale Deoxygenative Carbenation of Ru-2 to form Ru-1 Complexes.

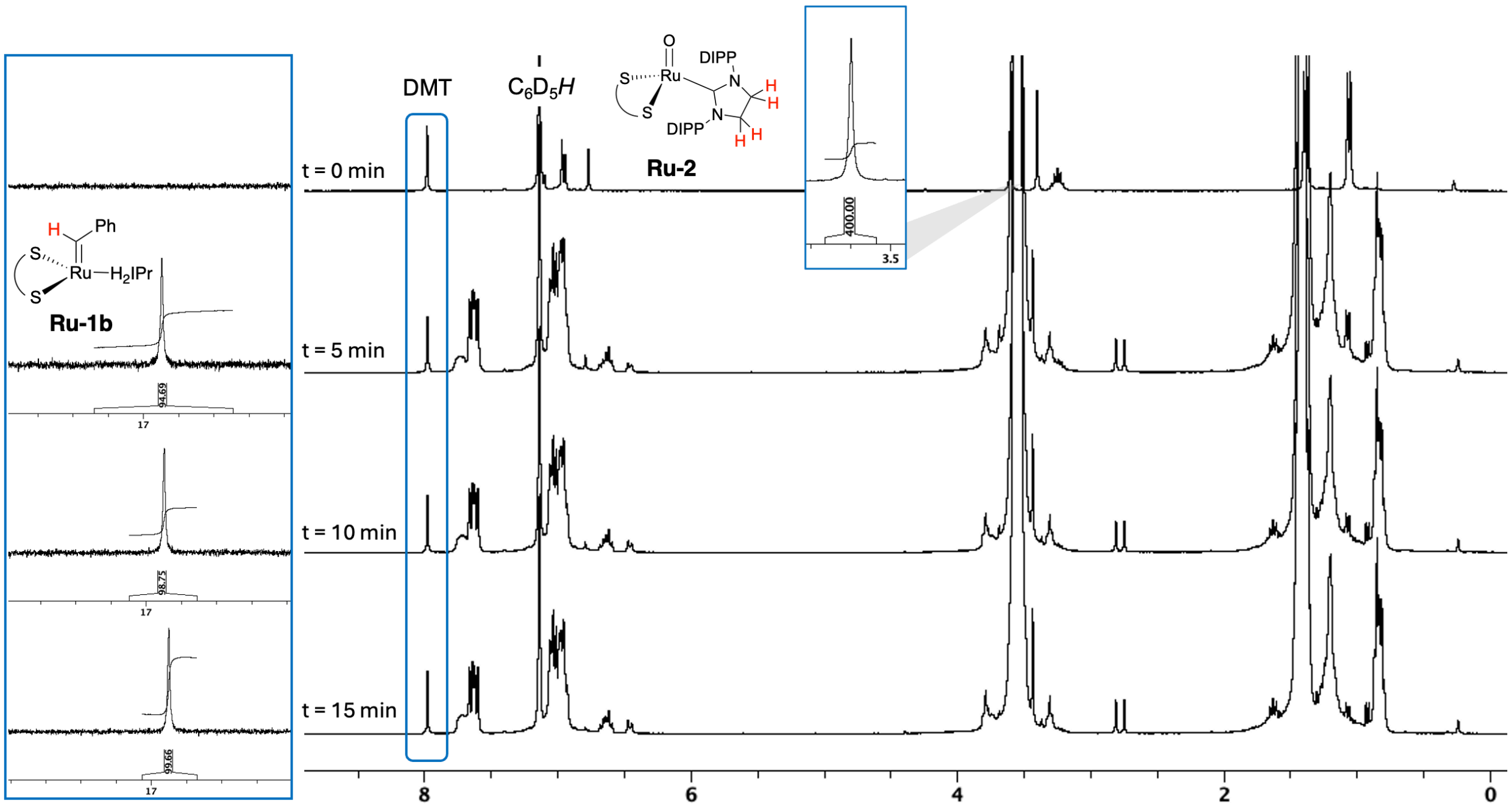

Figure S6. ^1H NMR spectra (300 MHz, C_6D_6) showing progress of the deoxygenative carbenation of **Ru-2** to form **Ru-1b**.

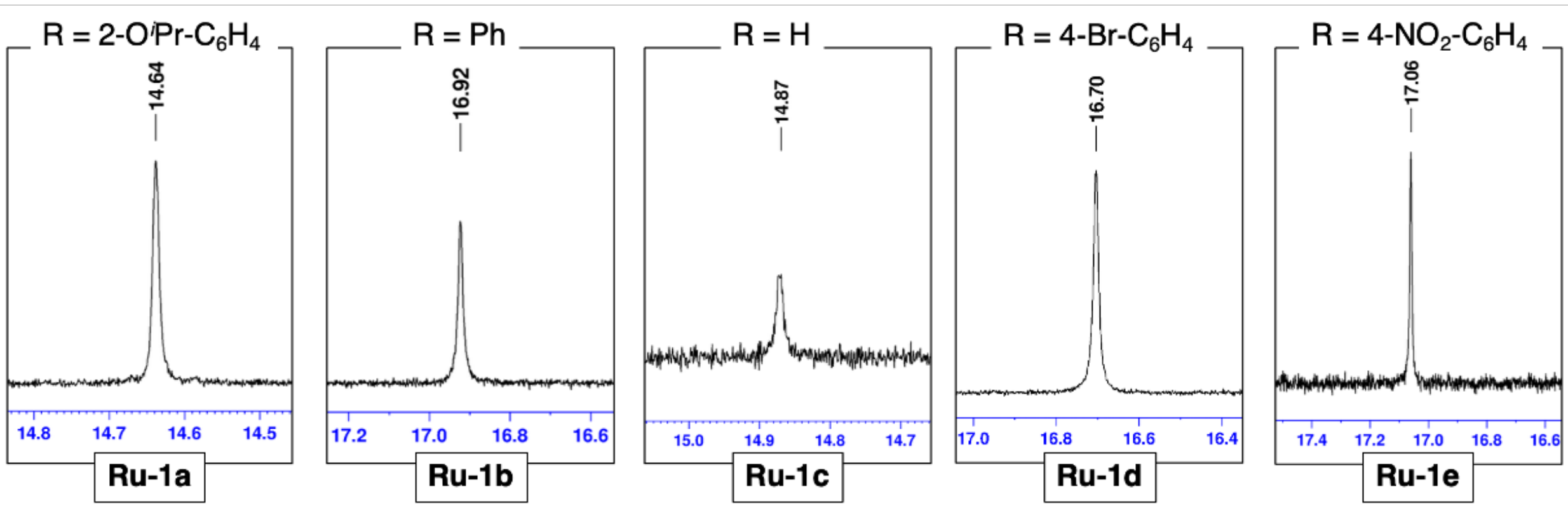

Figure S7. ^1H NMR spectra (300 MHz, C_6D_6) showing the key [Ru]=C*H*R signals for **Ru-1a-e**.

S3.5 Sequestration of $O{=}PPh_3$ as $[Li(O{=}PPh_3)_4]Cl$

Shown in Figures S8/S9 are spectra supporting identification of $[Li(O{=}PPh_3)_4]Cl$ as the co-product from oxidation of **Ru-5** to **Ru-2** in the presence of LiCl.

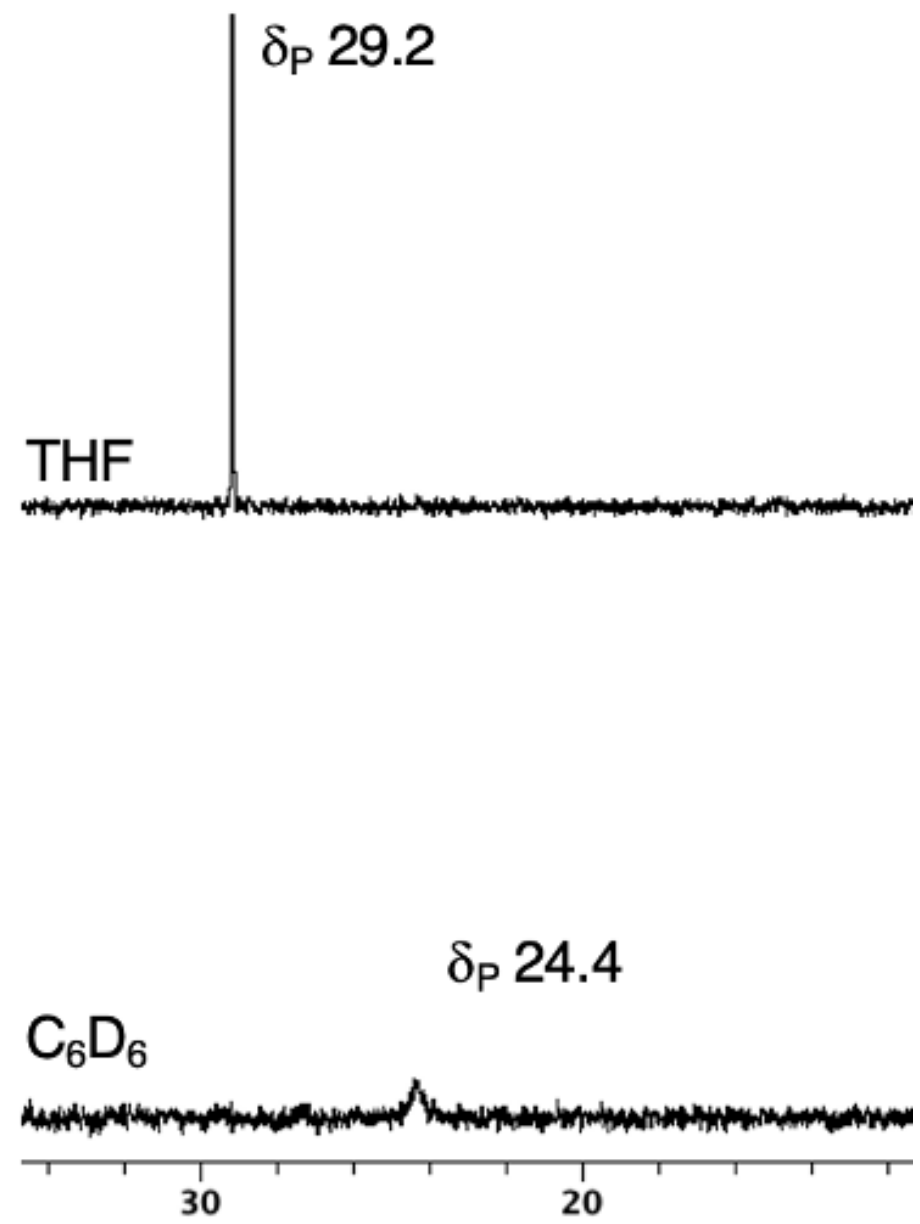

Figure S8. $^{31}P\{^1H\}$ NMR (121 MHz) spectra of the air-oxidation of **Ru-5** to **Ru-2** in the presence of LiCl, showing solubility of the phosphorus-containing product in THF, but not C_6D_6. Cf. a reported chemical shift of 26.9 ppm for $[Li(O{=}PPh_3)_4][AuI_2]$ in CD_2Cl_2.[16]

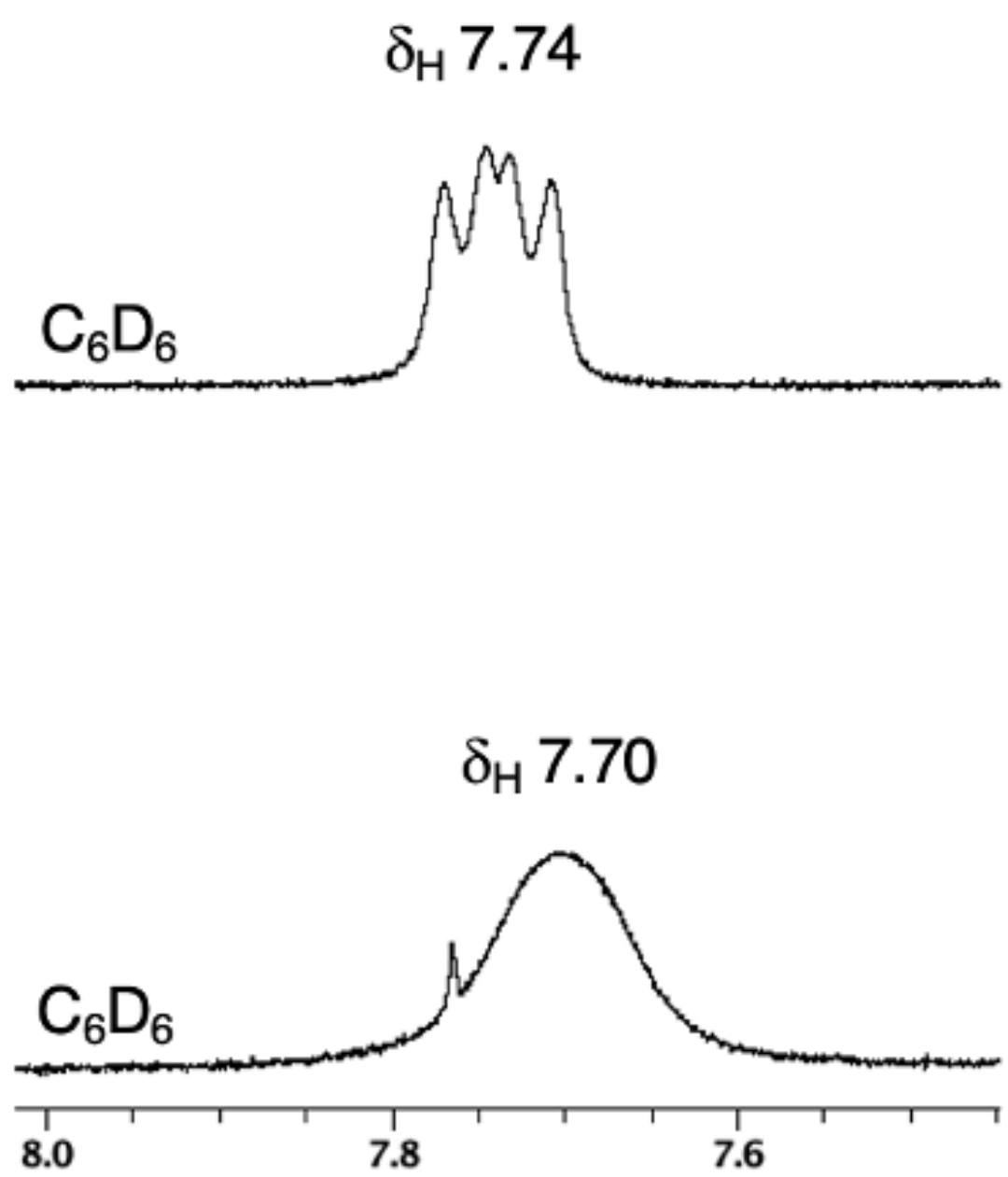

Figure S9. Comparison of the ^1H NMR signals (300 MHz, C_6D_6) for free $O{=}PPh_3$ (top) and putative $[Li(O{=}PPh_3)_4]Cl$ (bottom). A similar range (7.3–7.7 ppm), but no multiplicity, is reported for $[Li(O{=}PPh_3)_4][AuI_2]$ in CD_2Cl_2.[16]

S3.6 NMR Spectrum of Oxo Complex Ru-2 After 2 Months in Air.

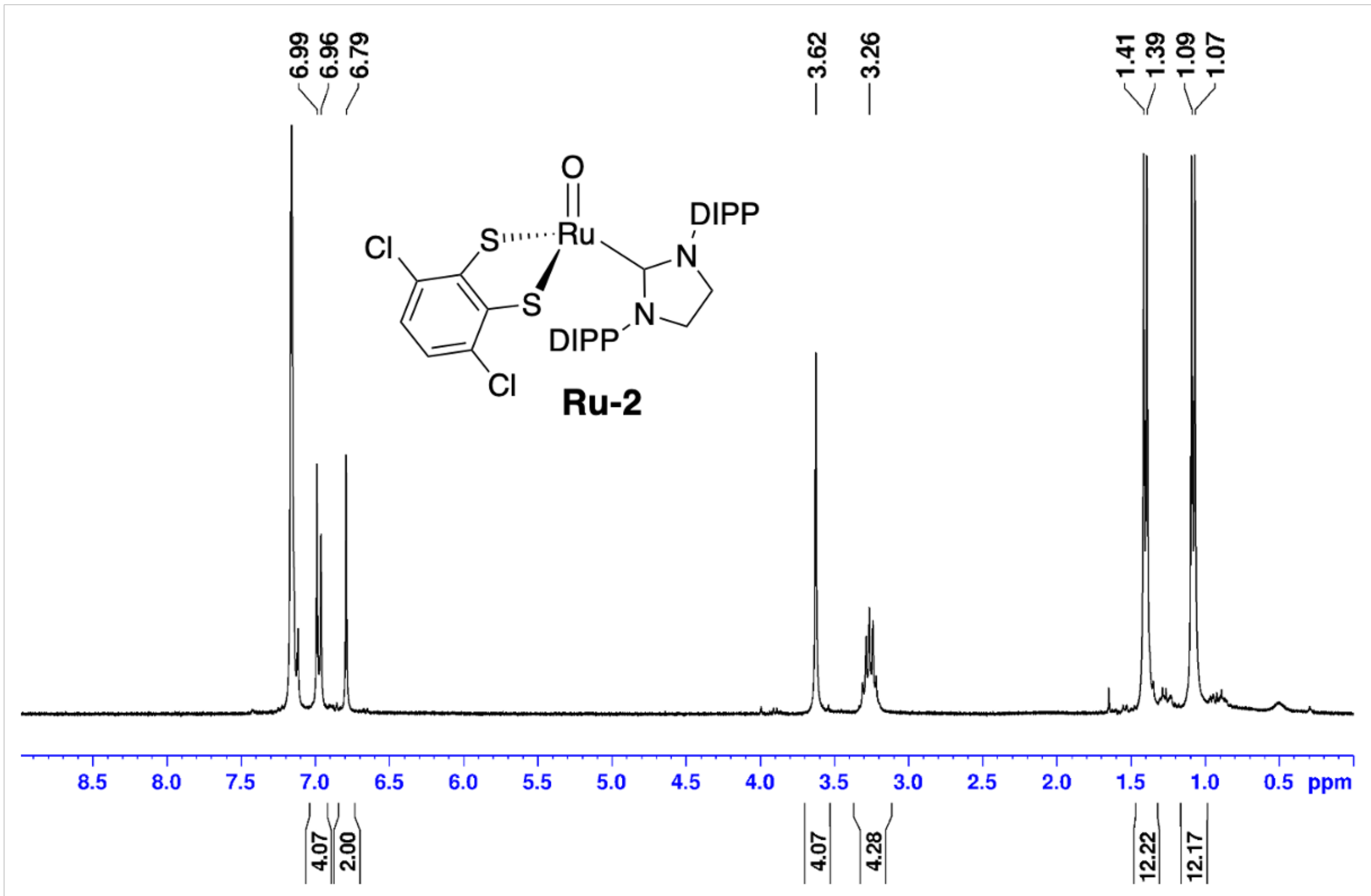

Figure S10. ^{1}H NMR spectrum (300 MHz, C_6D_6) of **Ru-2** after prolonged exposure to air (2 months). Cf. Figure S5a.

S3.7 NMR Spectra of Polymers.

(a) **Poly-8** generated in the glovebox using **Ru-1a** as initiator.

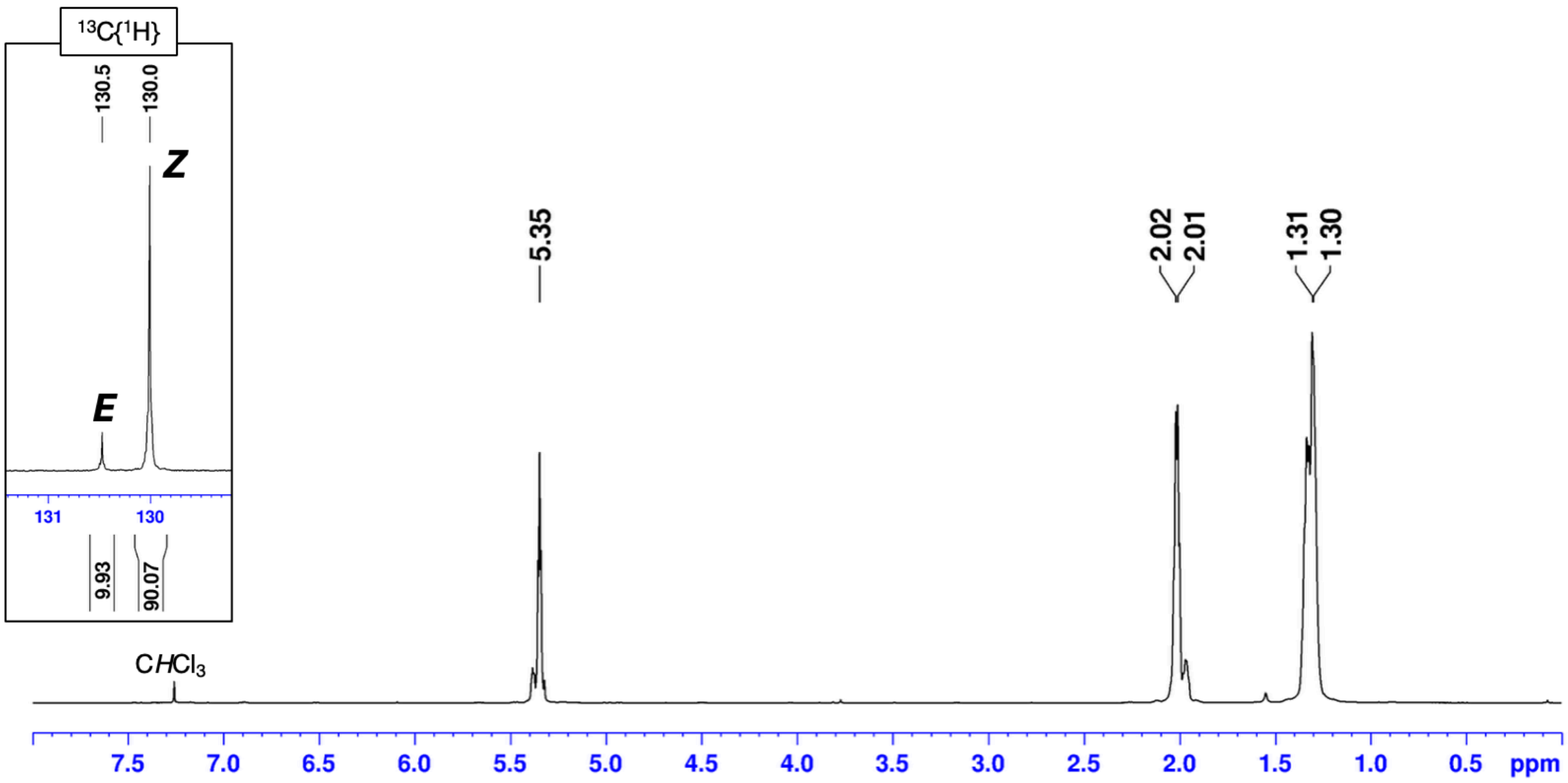

(b) **Poly-8** generated in the glovebox using oxo complex **Ru-2** + ylide **1b** as the initiator.

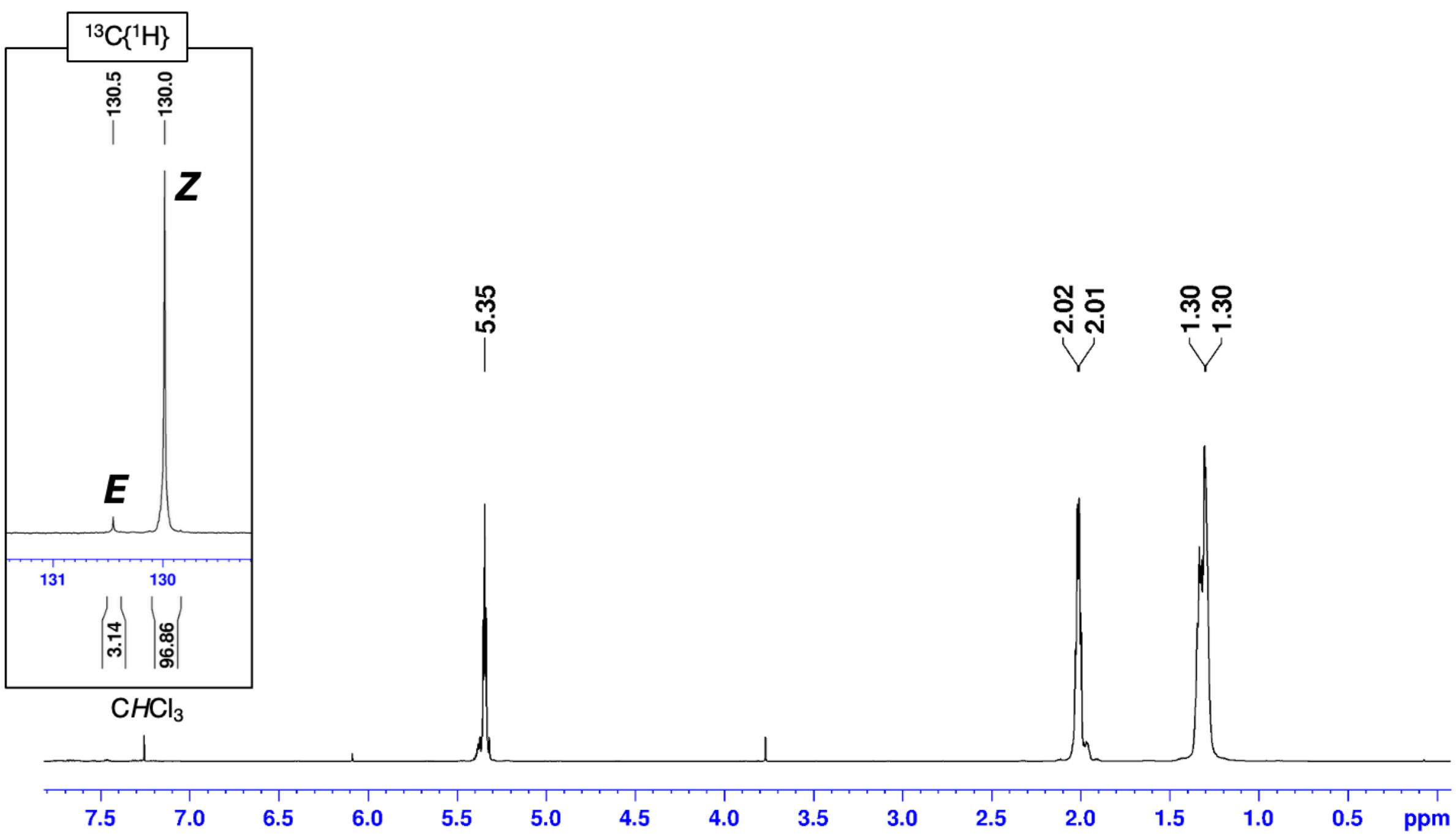

(c) **Poly-8**, generated in an N_2-filled vial in the fumehood using oxo complex **Ru-2** + ylide **1b** as the initiator.

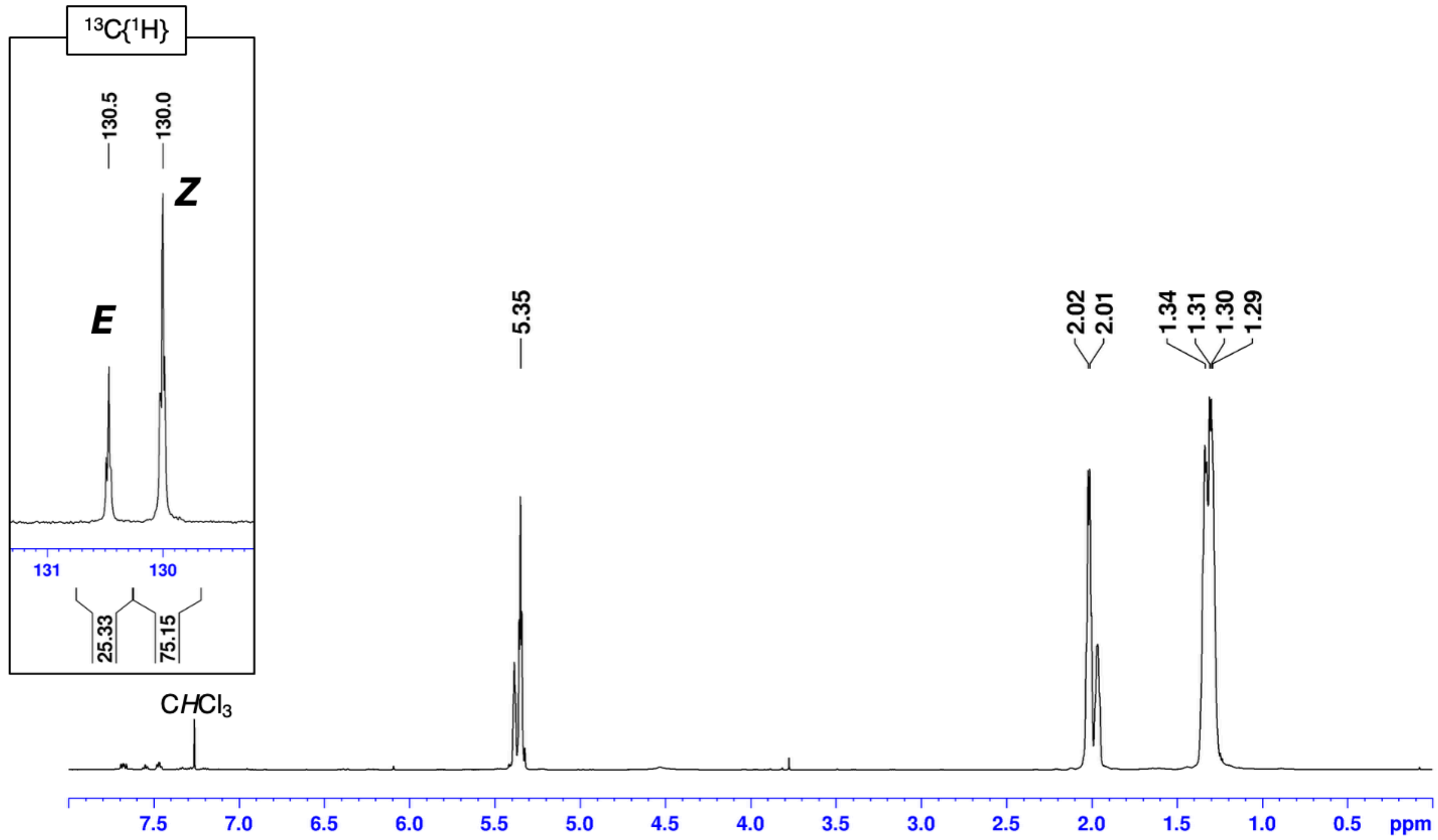

Figure S11. ^1H NMR spectrum (600 MHz, $CDCl_3$) of **poly-8** prepared via: (a) **Ru-1a** (glovebox). (b) **Ru-2** + **1b** (glovebox). (c) **Ru-2** + **1b** (fumehood). Inset shows $^{13}C\{^1H\}$ NMR signals (151 MHz) used to assess *E/Z* ratios.

S3.8 Expanded UV-Vis Spectra for Oxidation to form Ru-2.

(a) Oxidation of **Ru-1a** in air.

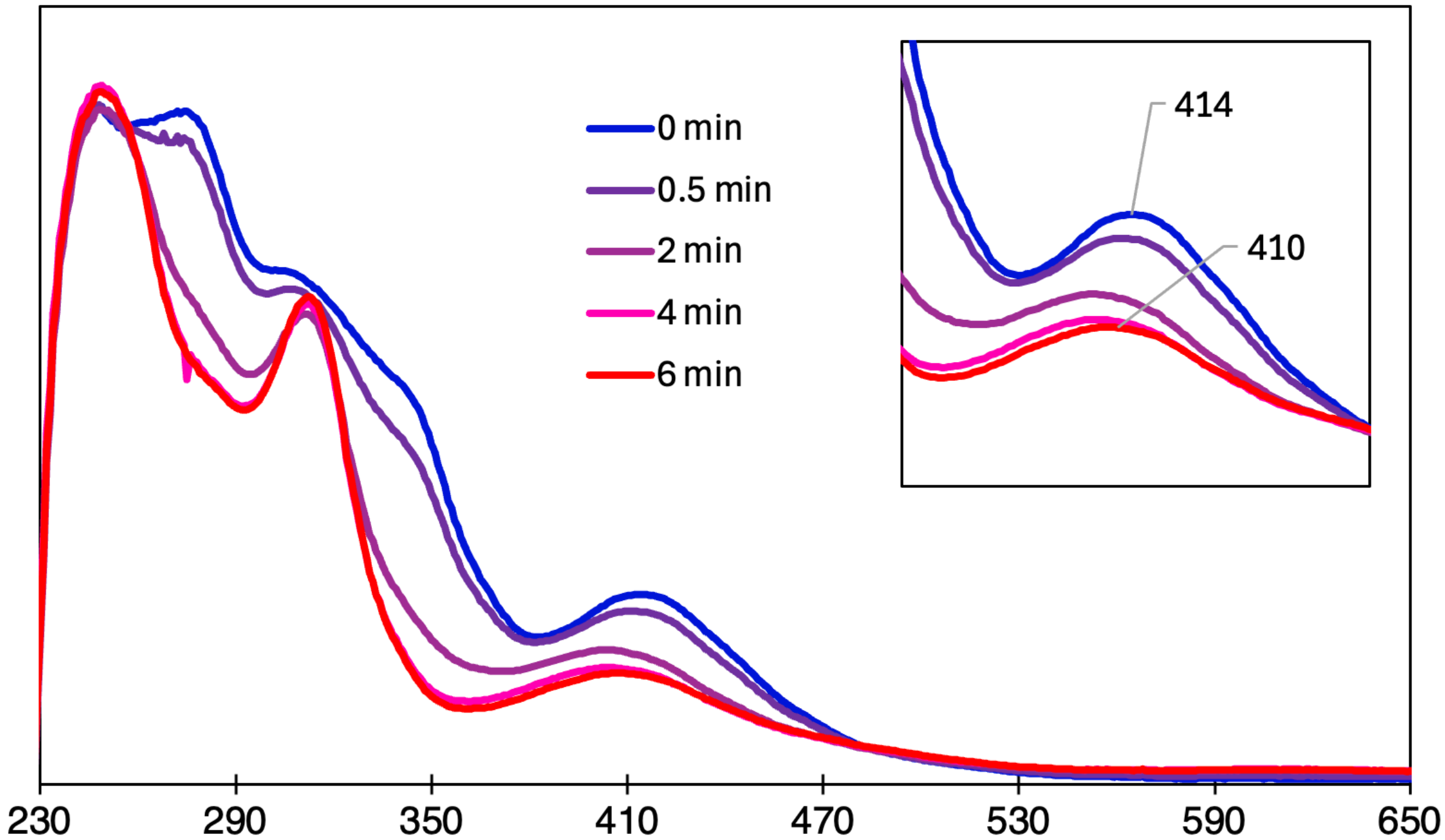

(b) Oxidation of **Ru-5** in air.

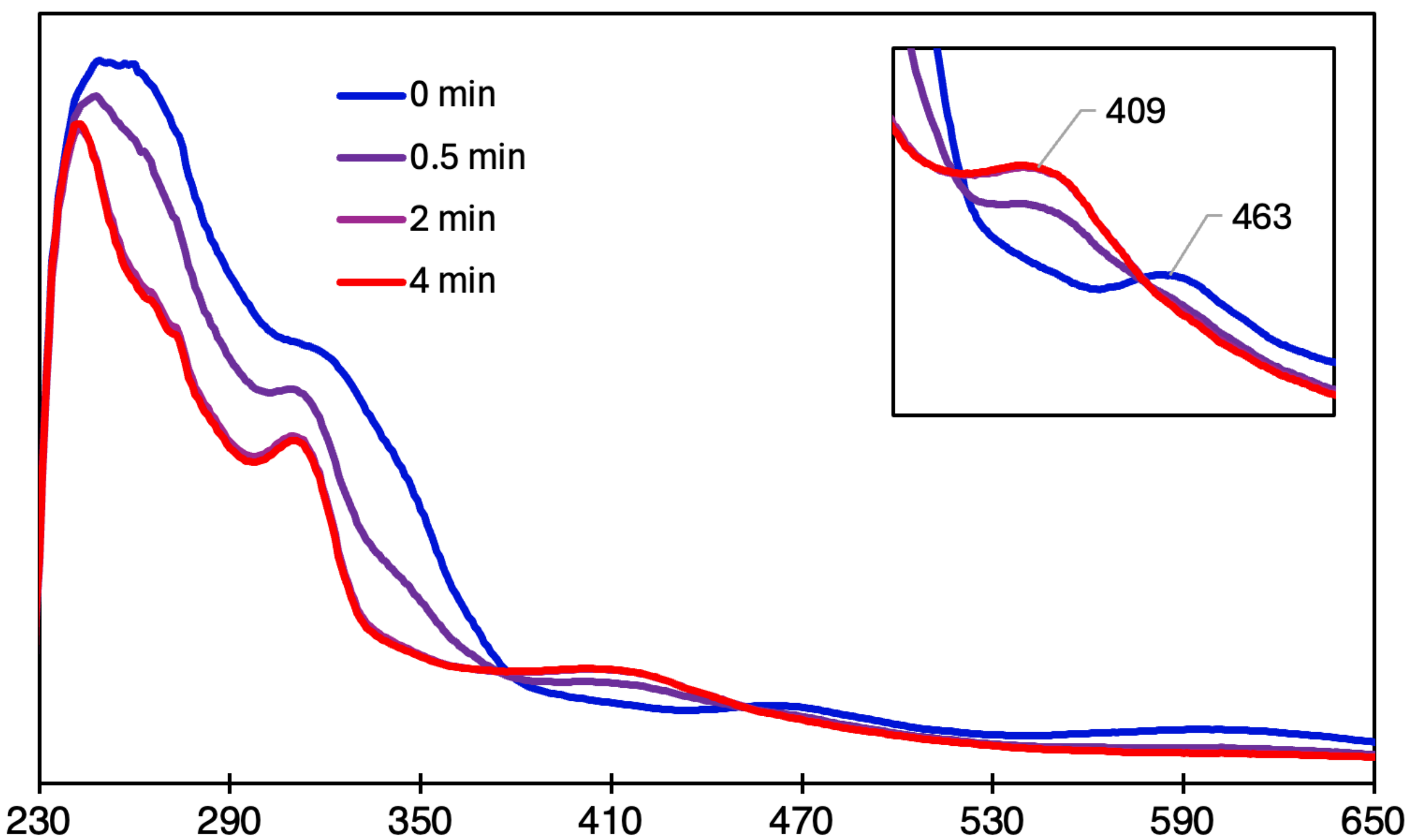

Figure S12. UV-vis spectra showing air-oxidation of ruthenium-dithiolene complexes to yield oxo complex **Ru-2**. (a) Oxidation of **Ru-1a**. (b) Oxidation of **Ru-5**. (Expanded from Scheme 3b in the main text).

S3.9 Representative GC Chromatograms for Quantification of Metathesis Reactions.

(a)

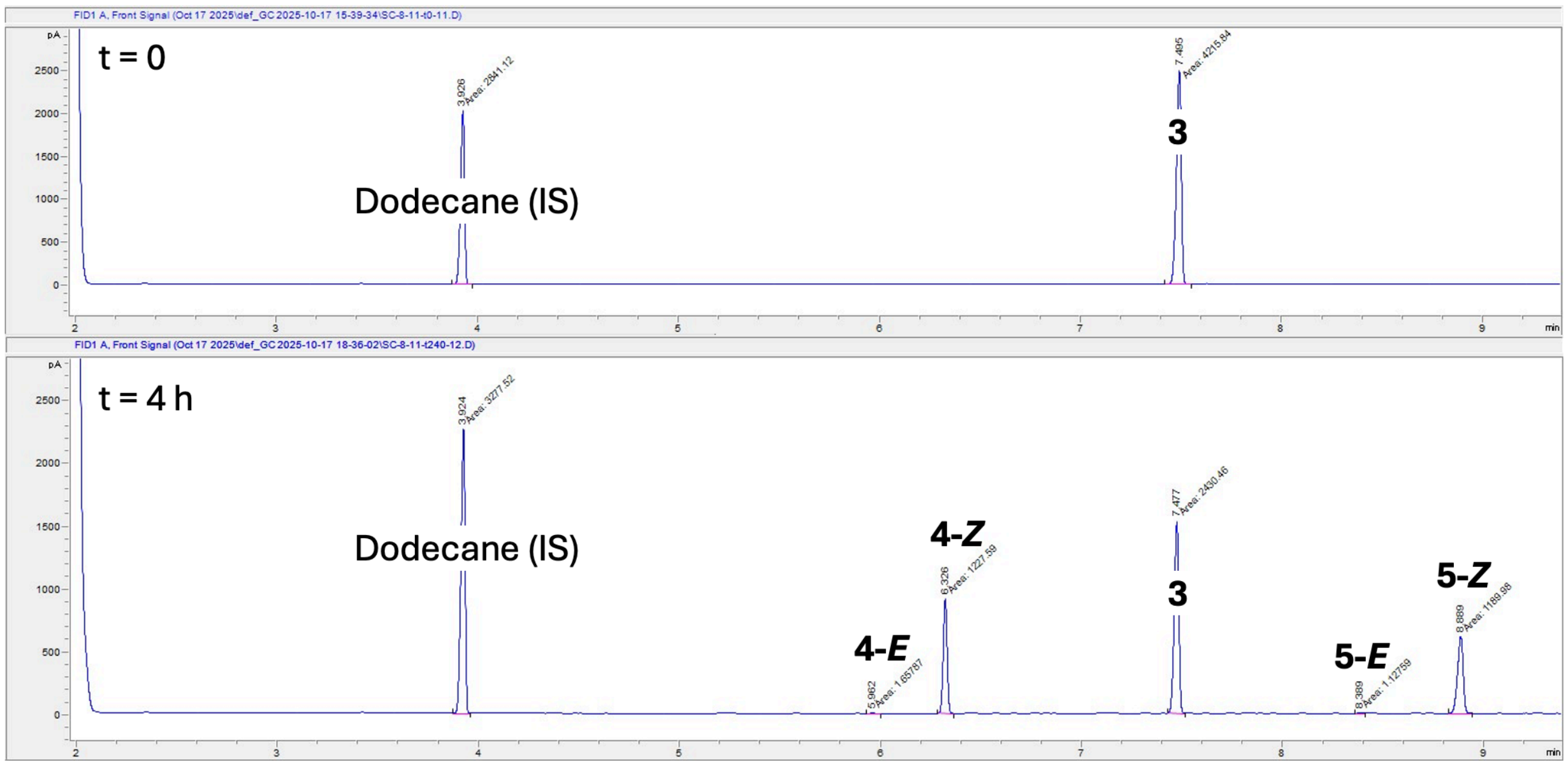

(b)

**Trial 1**						
**4-*Z***	**4-*E***	**4 %*Z***	**5-*Z***	**5-*E***	**5 %*Z***	**TOTAL**
**% yield**	**% yield**		**% yield**	**% yield**		**% yield**
0	0	N/A	0	0	N/A	0
23.1422199	0.0316337	99.863494	22.3116847	0.02416495	99.8918109	45.5097032
**Trial 2**						
**4-*Z***	**4-*E***	**4 %*Z***	**5-*Z***	**5-*E***	**5 %*Z***	**TOTAL**
**% yield**	**% yield**		**% yield**	**% yield**		**% yield**
0	0	N/A	0	0	N/A	0
23.2357562	0.0318968	99.8629135	22.2546378	0.0283228	99.8728948	45.5506136

Figure S13. (a) GC-FID chromatograms for self-metathesis of methyl oleate **3**. (b) Sample quantification between duplicate trials.

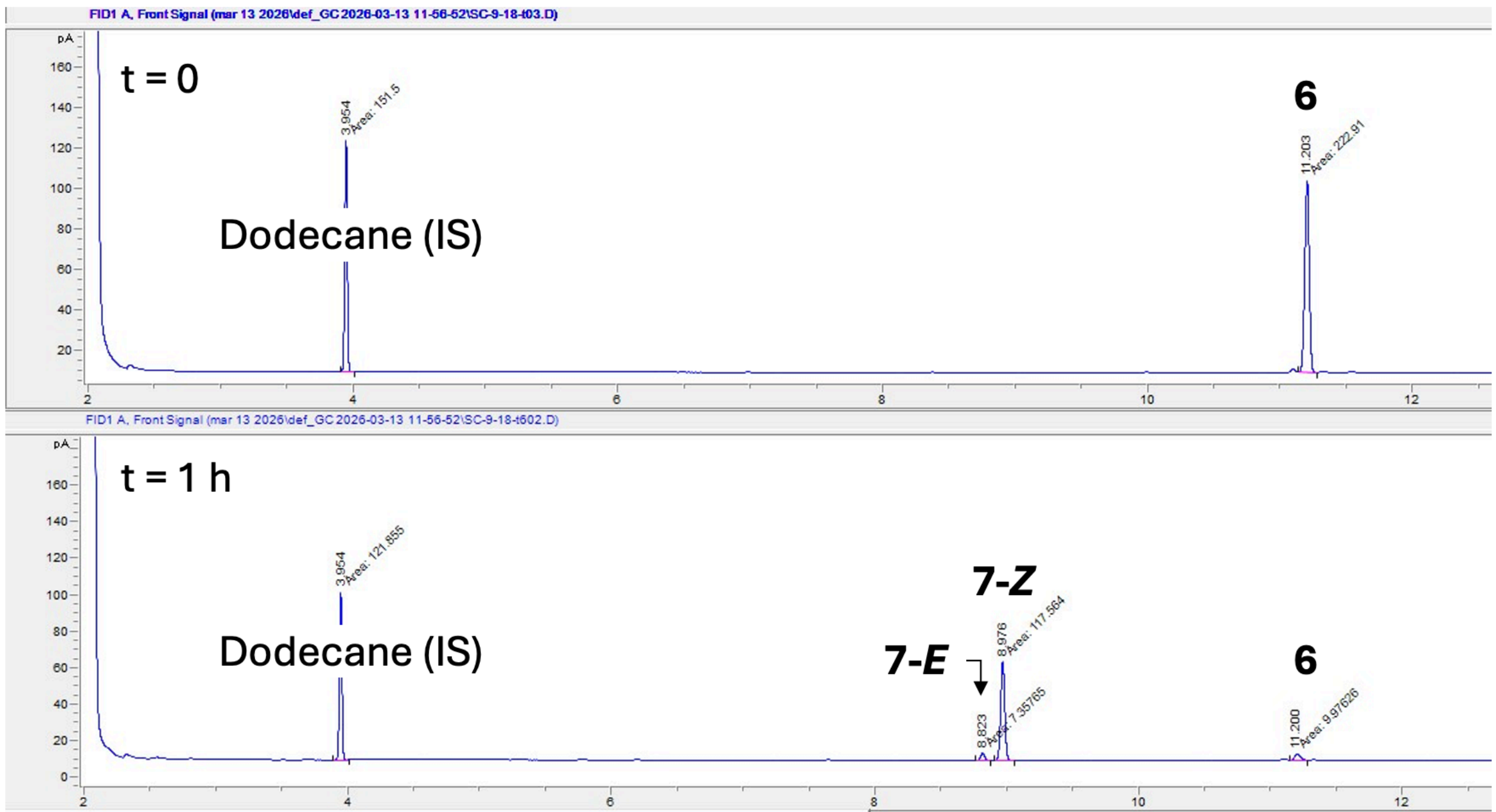

Figure S14. GC-FID chromatograms for mRCM of diene **6**.

S3.10 Molecular Weight Distribution for ROMP Polymers

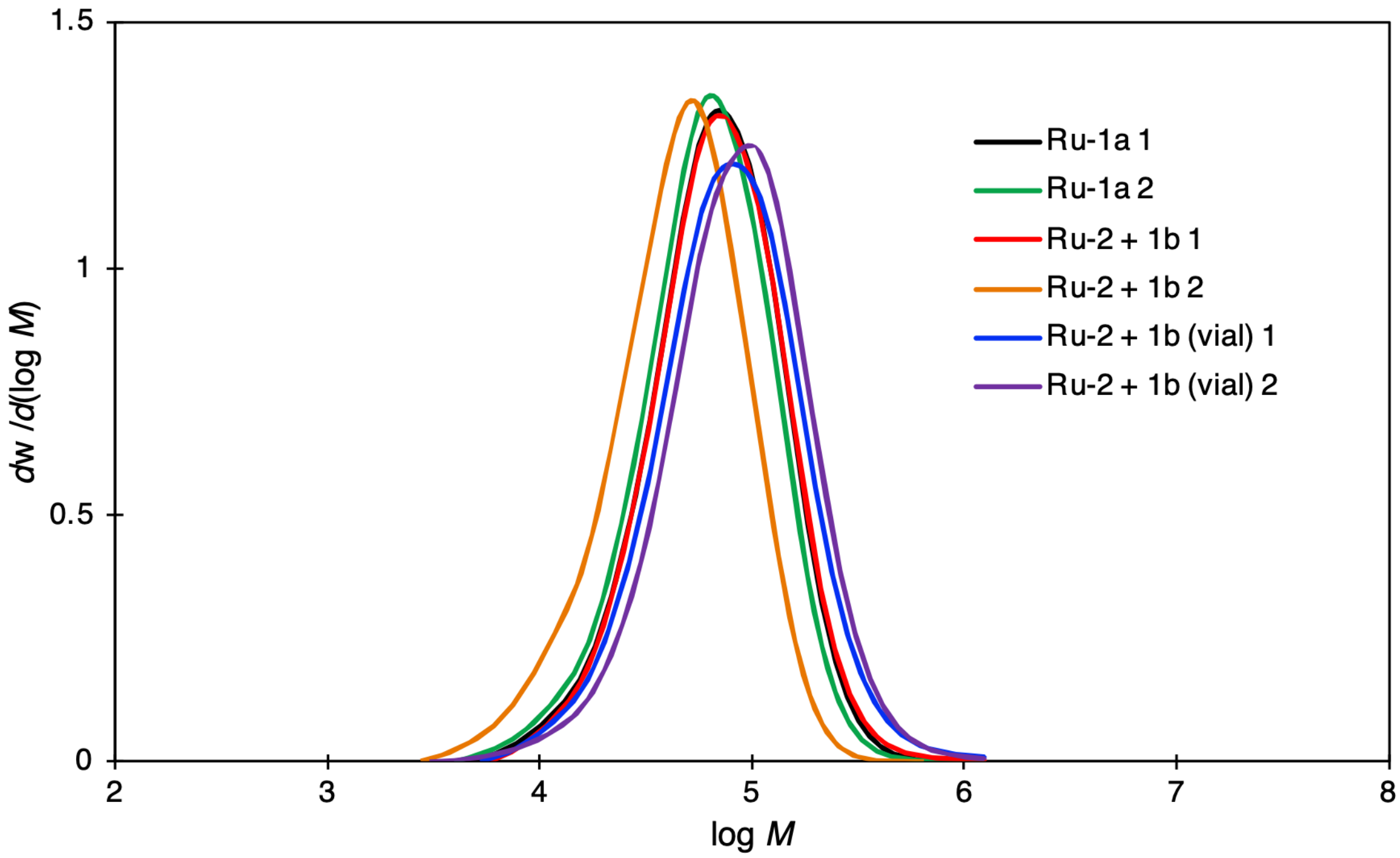

Figure S15. Molecular-weight distribution plotted as differential weight fraction, $dw/d(\log M)$, versus log M) for poly(cyclooctene) prepared using the initiators indicated. For reactions labelled (vial), initiator **Ru-1a** or ylide were injected after brief air-exposure, as described in the main text. Numbers 1 or 2 after each line refer to replicate samples.

S4. Steric Maps

S4.1 Steric Map of Ru(O)(S-S)(H_2IPr) **Ru-2**. Enlarged version of map in Scheme 2, main text.

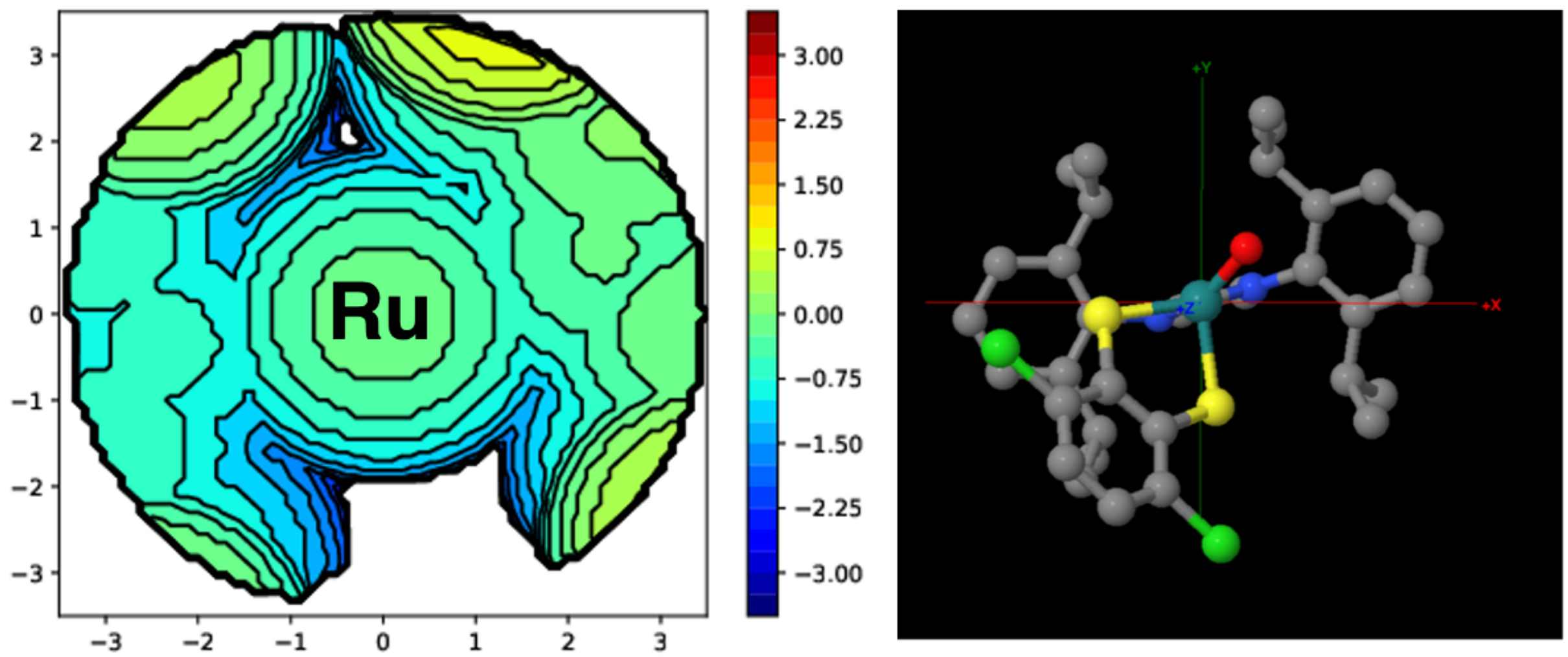

Figure S16. Steric map of Ru(O)(S-S)(H_2IPr) **Ru-2** (V_{bur} = 38%), generated using SambVca 2.1 using the reported[12] cif file from CCDC 2210344. The orientation of the molecular structure shown in the steric map is provided on the right.

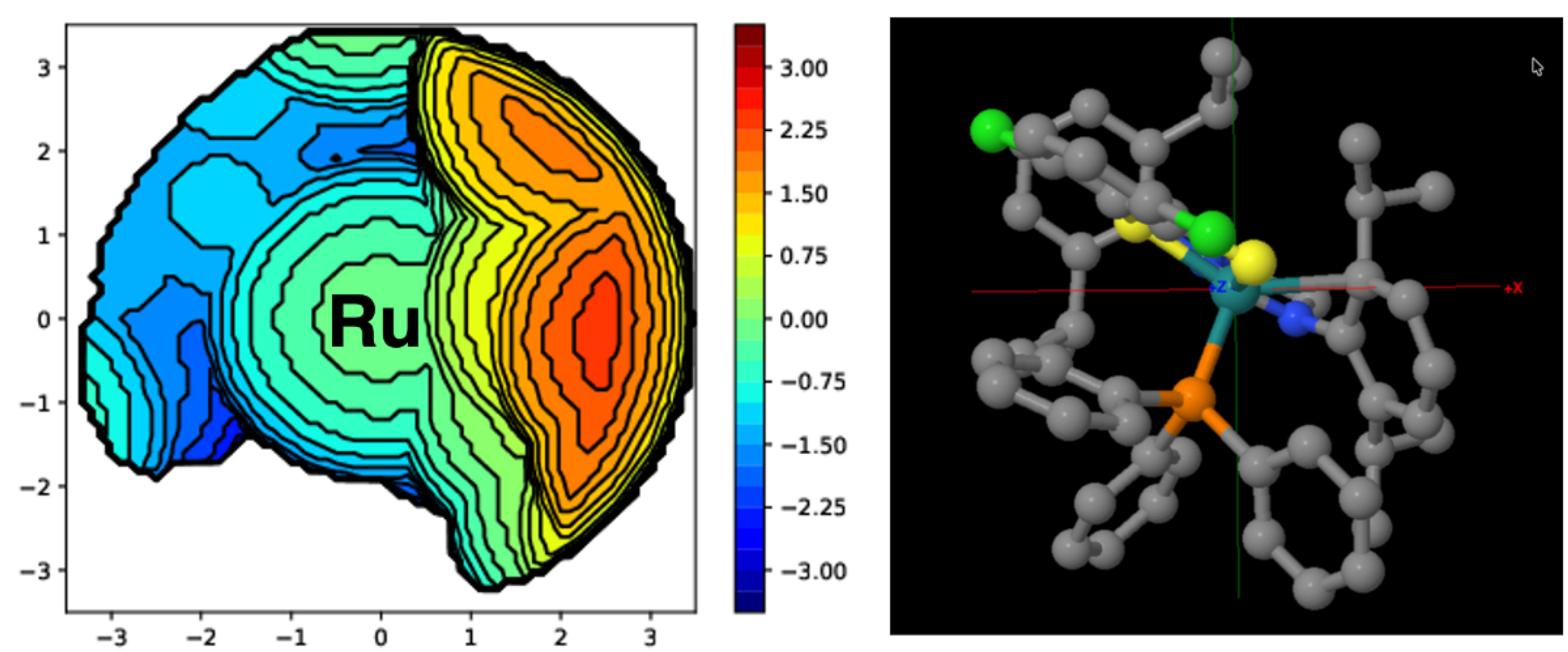

Figure S17. Steric map of Ru(S-S)(H_2IPr)(PPh_3) **Ru-5** (V_{bur} = 45%), generated using SambVca 2.1 using the cif file described in Section S5. The orientation of the molecular structure shown in the steric map is provided on the right.

S5. X-ray Crystallographic Analysis

S5.1 X-ray Crystallographic Analysis for Ru(S-S)(*p*-cymene)(PPh_3), Ru-4.

Table S5. Crystallographic Experimental Details

A. Crystal Data	
formula	$C_{38}H_{39}Cl_2OPRuS_2$
formula weight	778.75
crystal colour and habit[a]	yellow rod
crystal dimensions (mm)	0.12 × 0.02 × 0.02
crystal system	triclinic
space group	$P\bar{1}$ (No. 2)
unit cell parameters[b]	
a (Å)	11.3765(3)
b (Å)	11.6697(3)
c (Å)	15.1788(4)
α (°)	74.2668(18)
β (°)	89.4732(17)
γ (°)	64.8527(16)
V (Å^3)	1742.75(8)
Z	2
ρ_{calcd} (g cm^{-3})	1.484
μ (mm^{-1})	6.834
B. Data Collection and Refinement Conditions	
diffractometer	Bruker D8 Venture/PHOTON III[c]
radiation (λ [Å])	Cu K$_\alpha$ (1.54178) (microfocus source)
temperature (°C)	–100
scan type	ω and ϕ scans (1.0°) (θ-dep. exp. from 5-20 s)
data collection 2θ limit (°)	144.60
total data collected	62183 ($-14 \le h \le 14$, $-14 \le k \le 14$, $-18 \le l \le 18$)
independent reflections	6838 (R_{int} = 0.0859)
number of observed reflections (*NO*)	5886 [$F_o^2 \ge 2\sigma(F_o^2)$]
structure solution method	intrinsic phasing (*SHELXT-2018/2*)[17]
refinement method	full-matrix least-squares on F^2 (*SHELXL–2019/1*)[17]
absorption correction method	Gaussian integration (face-indexed)
range of transmission factors	0.9449–0.6938
data/restraints/parameters	6838 / 0 / 409
goodness-of-fit (S)[d] [all data]	1.028
final R indices[e]	
R_1 [$F_o^2 \ge 2\sigma(F_o^2)$]	0.0290
wR_2 [all data]	0.0722
largest difference peak and hole	0.564 and –0.379 e Å^{-3}

[a]Obtained by recrystallization from THF-Et_2O. [b]Obtained by least-squares refinement of 9859 reflections with 6.10° < 2θ < 144.40°. [c]Programs for diffractometer operation, data collection, data reduction and absorption correction were those supplied by Bruker. [d]$S = [\Sigma w(F_o^2 - F_c^2)^2/(n - p)]^{1/2}$ (n = number of data; p = number of parameters varied; $w = [\sigma^2(F_o^2) + (0.0335P)^2 + 0.7968P]^{-1}$ where $P = [\mathrm{Max}(F_o^2, 0) + 2F_c^2]/3$). [e]$R_1 = \Sigma||F_o| - |F_c||/\Sigma|F_o|$; $wR_2 = [\Sigma w(F_o^2 - F_c^2)^2/\Sigma w(F_o^4)]^{1/2}$.

Table S6. Atomic Coordinates and Equivalent Isotropic Displacement Parameters

(a) *Ru(S$_2$C$_6$H$_2$Cl$_2$)(η^6-^{i}PrC$_6$H$_4$Me)(PPh$_3$) atoms.*

Atom	*x*	*y*	*z*	U_{eq}, Å^2
Ru1	0.34808(2)	0.18911(2)	0.09696(2)	0.02238(6)*
Cl1	0.84504(7)	0.13686(8)	0.15028(6)	0.04877(18)*
Cl2	0.65799(9)	-0.28697(8)	0.34644(6)	0.0605(2)*
S1	0.55403(5)	0.19323(6)	0.09926(4)	0.02624(13)*
S2	0.45576(6)	-0.02406(6)	0.20296(4)	0.03049(14)*
P1	0.31671(6)	0.26900(6)	0.22418(4)	0.02413(13)*
C1	0.6592(2)	0.0467(3)	0.18246(16)	0.0281(5)*
C2	0.7892(3)	0.0202(3)	0.20529(19)	0.0355(6)*
C3	0.8759(3)	-0.0946(3)	0.2695(2)	0.0427(7)*
C4	0.8346(3)	-0.1887(3)	0.3115(2)	0.0448(7)*
C5	0.7073(3)	-0.1661(3)	0.29054(19)	0.0385(6)*
C6	0.6170(2)	-0.0494(3)	0.22693(17)	0.0300(5)*
C11	0.2205(2)	0.1165(3)	0.03626(18)	0.0310(5)*
C12	0.1394(2)	0.2367(3)	0.05457(18)	0.0305(5)*
C13	0.1602(2)	0.3507(2)	0.01806(17)	0.0286(5)*
C14	0.2570(2)	0.3509(2)	-0.04042(17)	0.0272(5)*
C15	0.3373(2)	0.2295(3)	-0.05852(17)	0.0295(5)*
C16	0.3203(2)	0.1157(3)	-0.02206(17)	0.0299(5)*
C17	0.2017(3)	-0.0065(3)	0.0728(2)	0.0436(7)*
C18	0.2624(3)	0.4803(3)	-0.08896(19)	0.0363(6)*
C19	0.3978(3)	0.4690(4)	-0.1028(3)	0.0527(8)*
C20	0.1746(3)	0.5436(3)	-0.1812(2)	0.0471(7)*
C21	0.4583(2)	0.2256(3)	0.30521(16)	0.0292(5)*
C22	0.5209(3)	0.3058(3)	0.29984(19)	0.0385(6)*
C23	0.6319(3)	0.2645(4)	0.3601(2)	0.0498(8)*
C24	0.6814(3)	0.1433(4)	0.4261(2)	0.0502(8)*
C25	0.6200(3)	0.0623(3)	0.4329(2)	0.0448(7)*
C26	0.5084(3)	0.1026(3)	0.37296(18)	0.0352(6)*
C31	0.2091(2)	0.2273(3)	0.30466(17)	0.0298(5)*
C32	0.1806(3)	0.2801(3)	0.37841(19)	0.0384(6)*
C33	0.0951(3)	0.2548(4)	0.4373(2)	0.0475(8)*
C34	0.0395(3)	0.1748(4)	0.4249(2)	0.0483(8)*
C35	0.0698(3)	0.1187(3)	0.3539(2)	0.0428(7)*
C36	0.1550(3)	0.1454(3)	0.29368(19)	0.0348(6)*
C41	0.2366(2)	0.4507(2)	0.18422(17)	0.0274(5)*
C42	0.1100(3)	0.5263(3)	0.20059(19)	0.0324(5)*
C43	0.0489(3)	0.6625(3)	0.1597(2)	0.0396(6)*
C44	0.1135(3)	0.7253(3)	0.1042(2)	0.0412(7)*
C45	0.2391(3)	0.6522(3)	0.0872(2)	0.0384(6)*
C46	0.2990(3)	0.5156(3)	0.12572(18)	0.0306(5)*
(b) Solvent THF atoms				
O1S	0.1760(3)	0.7576(3)	0.4071(2)	0.0806(9)*
C1S	0.2689(6)	0.7705(5)	0.3488(4)	0.0922(16)*
C2S	0.3623(6)	0.6381(6)	0.3475(5)	0.113(2)*
C3S	0.3307(6)	0.5464(6)	0.4172(6)	0.126(3)*
C4S	0.2118(5)	0.6212(5)	0.4463(4)	0.0951(16)*

Anisotropically-refined atoms marked (*). Form of the anisotropic displacement parameter: $\exp[-2\pi^2(h^2a^{*2}U_{11} + k^2b^{*2}U_{22} + l^2c^{*2}U_{33} + 2klb^*c^*U_{23} + 2hla^*c^*U_{13} + 2hka^*b^*U_{12})]$.

Table S7. Selected Interatomic Distances (Å).

(a) Involving $Ru(S_2C_6H_2Cl_2)(\eta^6\text{-}^iPrC_6H_4Me)(PPh_3)$ atoms.

Atom1	Atom2	Distance	Atom1	Atom2	Distance
Ru1	S1	2.3645(6)	C12	C13	1.414(4)
Ru1	S2	2.3565(6)	C13	C14	1.409(4)
Ru1	P1	2.3251(6)	C14	C15	1.422(4)
Ru1	C11	2.273(2)	C14	C18	1.518(4)
Ru1	C12	2.252(2)	C15	C16	1.388(4)
Ru1	C13	2.226(2)	C18	C19	1.507(4)
Ru1	C14	2.287(2)	C18	C20	1.530(4)
Ru1	C15	2.271(2)	C21	C22	1.384(4)
Ru1	C16	2.275(2)	C21	C26	1.399(4)
Cl1	C2	1.750(3)	C22	C23	1.389(4)
Cl2	C5	1.743(3)	C23	C24	1.373(5)
S1	C1	1.752(3)	C24	C25	1.378(5)
S2	C6	1.755(3)	C25	C26	1.394(4)
P1	C21	1.835(3)	C31	C32	1.391(4)
P1	C31	1.840(3)	C31	C36	1.382(4)
P1	C41	1.834(3)	C32	C33	1.385(4)
C1	C2	1.402(4)	C33	C34	1.380(5)
C1	C6	1.409(4)	C34	C35	1.376(5)
C2	C3	1.380(4)	C35	C36	1.399(4)
C3	C4	1.374(5)	C41	C42	1.395(4)
C4	C5	1.380(4)	C41	C46	1.395(4)
C5	C6	1.399(4)	C42	C43	1.388(4)
C11	C12	1.415(4)	C43	C44	1.378(4)
C11	C16	1.431(4)	C44	C45	1.382(4)
C11	C17	1.497(4)	C45	C46	1.388(4)

(b) Within the solvent THF molecules

Atom1	Atom2	Distance	Atom1	Atom2	Distance
O1S	C1S	1.403(6)	C2S	C3S	1.449(8)
O1S	C4S	1.412(6)	C3S	C4S	1.408(7)
C1S	C2S	1.462(7)			

Table S8. Selected Interatomic Angles (°)

(a) Involving $Ru(S_2C_6H_2Cl_2)(\eta^6\text{-}^iPrC_6H_4Me)(PPh_3)$ atoms.

Atom1	Atom2	Atom3	Angle	Atom1	Atom2	Atom3	Angle
S1	Ru1	S2	86.01(2)	Cl1	C2	C3	118.2(2)
S1	Ru1	P1	87.16(2)	C1	C2	C3	122.8(3)
S1	Ru1	C11	144.70(7)	C2	C3	C4	118.9(3)
S1	Ru1	C12	162.23(7)	C3	C4	C5	119.8(3)
S1	Ru1	C13	126.76(7)	Cl2	C5	C4	118.5(2)
S1	Ru1	C14	96.43(6)	Cl2	C5	C6	119.3(2)
S1	Ru1	C15	89.55(6)	C4	C5	C6	122.3(3)
S1	Ru1	C16	109.26(7)	S2	C6	C1	120.97(19)
S2	Ru1	P1	86.51(2)	S2	C6	C5	120.8(2)
S2	Ru1	C11	88.28(7)	C1	C6	C5	118.3(2)
S2	Ru1	C12	110.88(7)	Ru1	C11	C12	70.98(14)
S2	Ru1	C13	147.19(7)	Ru1	C11	C16	71.75(14)
S2	Ru1	C14	159.62(7)	Ru1	C11	C17	130.39(19)
S2	Ru1	C15	123.78(7)	C12	C11	C16	118.1(2)
S2	Ru1	C16	94.86(7)	C12	C11	C17	122.2(2)
P1	Ru1	C11	127.22(7)	C16	C11	C17	119.7(2)
P1	Ru1	C12	98.95(7)	Ru1	C12	C11	72.58(14)
P1	Ru1	C13	93.03(7)	Ru1	C12	C13	70.62(13)
P1	Ru1	C14	113.79(7)	C11	C12	C13	120.3(2)
P1	Ru1	C15	149.23(7)	Ru1	C13	C12	72.59(14)
P1	Ru1	C16	163.57(7)	Ru1	C13	C14	74.15(14)
C11	Ru1	C12	36.44(10)	C12	C13	C14	121.8(2)
C11	Ru1	C13	66.06(9)	Ru1	C14	C13	69.50(14)
C11	Ru1	C14	78.02(9)	Ru1	C14	C15	71.25(14)
C11	Ru1	C15	65.22(9)	Ru1	C14	C18	135.69(17)
C11	Ru1	C16	36.69(9)	C13	C14	C15	117.2(2)
C12	Ru1	C13	36.79(10)	C13	C14	C18	119.7(2)
C12	Ru1	C14	65.82(9)	C15	C14	C18	122.8(2)
C12	Ru1	C15	76.63(9)	Ru1	C15	C14	72.40(14)
C12	Ru1	C16	65.25(9)	Ru1	C15	C16	72.39(14)
C13	Ru1	C14	36.35(9)	C14	C15	C16	122.0(2)
C13	Ru1	C15	64.96(9)	Ru1	C16	C11	71.56(14)
C13	Ru1	C16	76.97(9)	Ru1	C16	C15	72.08(14)
C14	Ru1	C15	36.35(9)	C11	C16	C15	120.6(2)
C14	Ru1	C16	65.18(9)	C14	C18	C19	115.0(2)
C15	Ru1	C16	35.54(10)	C14	C18	C20	108.8(2)
Ru1	S1	C1	105.63(9)	C19	C18	C20	110.6(3)
Ru1	S2	C6	105.89(9)	P1	C21	C22	123.2(2)
Ru1	P1	C21	119.68(8)	P1	C21	C26	118.0(2)
Ru1	P1	C31	117.26(9)	C22	C21	C26	118.7(2)
Ru1	P1	C41	109.03(8)	C21	C22	C23	120.6(3)
C21	P1	C31	100.56(11)	C22	C23	C24	120.5(3)
C21	P1	C41	104.97(12)	C23	C24	C25	119.8(3)
C31	P1	C41	103.57(12)	C24	C25	C26	120.3(3)
S1	C1	C2	120.8(2)	C21	C26	C25	120.1(3)
S1	C1	C6	121.22(18)	P1	C31	C32	119.3(2)
C2	C1	C6	117.9(2)	P1	C31	C36	121.8(2)
Cl1	C2	C1	119.0(2)	C32	C31	C36	118.9(2)

Table S8. Selected Interatomic Angles (continued).

Atom1	Atom2	Atom3	Angle	Atom1	Atom2	Atom3	Angle
C31	C32	C33	120.1(3)	C42	C41	C46	118.2(2)
C32	C33	C34	120.6(3)	C41	C42	C43	120.4(3)
C33	C34	C35	119.9(3)	C42	C43	C44	120.5(3)
C34	C35	C36	119.6(3)	C43	C44	C45	120.0(3)
C31	C36	C35	120.8(3)	C44	C45	C46	119.7(3)
P1	C41	C42	123.1(2)	C41	C46	C45	121.2(3)
P1	C41	C46	118.27(19)				

(b) Within the solvent THF molecules

Atom1	Atom2	Atom3	Angle	Atom1	Atom2	Atom3	Angle
C1S	O1S	C4S	108.1(4)	C2S	C3S	C4S	107.4(5)
O1S	C1S	C2S	108.2(4)	O1S	C4S	C3S	109.6(4)
C1S	C2S	C3S	105.8(4)				

Table S9. Torsional Angles (°)

Atom1	Atom2	Atom3	Atom4	Angle
S2	Ru1	S1	C1	-4.59(8)
P1	Ru1	S1	C1	82.10(8)
C11	Ru1	S1	C1	-85.93(14)
C12	Ru1	S1	C1	-167.1(2)
C13	Ru1	S1	C1	173.77(12)
C14	Ru1	S1	C1	-164.27(11)
C15	Ru1	S1	C1	-128.50(11)
C16	Ru1	S1	C1	-98.35(11)
S1	Ru1	S2	C6	3.90(9)
P1	Ru1	S2	C6	-83.49(9)
C11	Ru1	S2	C6	149.05(11)
C12	Ru1	S2	C6	178.26(11)
C13	Ru1	S2	C6	-173.67(15)
C14	Ru1	S2	C6	101.7(2)
C15	Ru1	S2	C6	90.69(12)
C16	Ru1	S2	C6	112.93(11)
S1	Ru1	P1	C21	-30.26(10)
S1	Ru1	P1	C31	-152.33(9)
S1	Ru1	P1	C41	90.48(9)
S2	Ru1	P1	C21	55.91(10)
S2	Ru1	P1	C31	-66.16(9)
S2	Ru1	P1	C41	176.65(9)
C11	Ru1	P1	C21	141.09(13)
C11	Ru1	P1	C31	19.02(13)
C11	Ru1	P1	C41	-98.17(12)
C12	Ru1	P1	C21	166.52(12)
C12	Ru1	P1	C31	44.45(12)
C12	Ru1	P1	C41	-72.74(11)
C13	Ru1	P1	C21	-156.94(12)
C13	Ru1	P1	C31	80.99(11)
C13	Ru1	P1	C41	-36.21(11)
C14	Ru1	P1	C21	-126.04(12)
C14	Ru1	P1	C31	111.89(11)
C14	Ru1	P1	C41	-5.31(11)
C15	Ru1	P1	C21	-114.61(16)
C15	Ru1	P1	C31	123.32(15)
C15	Ru1	P1	C41	6.12(15)
C16	Ru1	P1	C21	151.3(3)
C16	Ru1	P1	C31	29.2(3)
C16	Ru1	P1	C41	-88.0(3)
S1	Ru1	C11	C12	-149.50(13)
S1	Ru1	C11	C16	-19.9(2)
S1	Ru1	C11	C17	94.0(3)
S2	Ru1	C11	C12	129.87(15)
S2	Ru1	C11	C16	-100.50(15)
S2	Ru1	C11	C17	13.3(3)
P1	Ru1	C11	C12	45.57(17)
P1	Ru1	C11	C16	175.20(12)
P1	Ru1	C11	C17	-71.0(3)
C12	Ru1	C11	C16	129.6(2)
C12	Ru1	C11	C17	-116.5(3)
C13	Ru1	C11	C12	-29.09(15)
C13	Ru1	C11	C16	100.54(17)
C13	Ru1	C11	C17	-145.6(3)
C14	Ru1	C11	C12	-65.32(16)
C14	Ru1	C11	C16	64.31(15)
C14	Ru1	C11	C17	178.1(3)
C15	Ru1	C11	C12	-101.33(17)
C15	Ru1	C11	C16	28.30(15)
C15	Ru1	C11	C17	142.1(3)
C16	Ru1	C11	C12	-129.6(2)
C16	Ru1	C11	C17	113.8(3)
S1	Ru1	C12	C11	106.0(2)
S1	Ru1	C12	C13	-26.0(3)
S2	Ru1	C12	C11	-55.20(16)
S2	Ru1	C12	C13	172.71(13)
P1	Ru1	C12	C11	-144.86(14)
P1	Ru1	C12	C13	83.05(14)
C11	Ru1	C12	C13	-132.1(2)
C13	Ru1	C12	C11	132.1(2)
C14	Ru1	C12	C11	103.00(17)
C14	Ru1	C12	C13	-29.09(15)
C15	Ru1	C12	C11	66.21(16)
C15	Ru1	C12	C13	-65.89(16)
C16	Ru1	C12	C11	30.44(15)
C16	Ru1	C12	C13	-101.65(17)
S1	Ru1	C13	C12	170.37(12)
S1	Ru1	C13	C14	38.82(17)
S2	Ru1	C13	C12	-12.6(2)
S2	Ru1	C13	C14	-144.19(13)
P1	Ru1	C13	C12	-100.90(14)
P1	Ru1	C13	C14	127.55(14)
C11	Ru1	C13	C12	28.83(15)
C11	Ru1	C13	C14	-102.71(16)
C12	Ru1	C13	C14	-131.5(2)
C14	Ru1	C13	C12	131.5(2)
C15	Ru1	C13	C12	101.45(17)
C15	Ru1	C13	C14	-30.10(15)
C16	Ru1	C13	C12	65.91(15)
C16	Ru1	C13	C14	-65.64(15)
S1	Ru1	C14	C13	-149.64(14)
S1	Ru1	C14	C15	80.42(14)
S1	Ru1	C14	C18	-37.6(3)
S2	Ru1	C14	C13	114.5(2)
S2	Ru1	C14	C15	-15.5(3)
S2	Ru1	C14	C18	-133.5(2)
P1	Ru1	C14	C13	-59.91(15)
P1	Ru1	C14	C15	170.15(12)

Table S9. Torsional Angles (continued).

Atom1	Atom2	Atom3	Atom4	Angle
P1	Ru1	C14	C18	52.1(3)
C11	Ru1	C14	C13	65.70(15)
C11	Ru1	C14	C15	-64.24(15)
C11	Ru1	C14	C18	177.7(3)
C12	Ru1	C14	C13	29.43(15)
C12	Ru1	C14	C15	-100.51(16)
C12	Ru1	C14	C18	141.4(3)
C13	Ru1	C14	C15	-129.9(2)
C13	Ru1	C14	C18	112.0(3)
C15	Ru1	C14	C13	129.9(2)
C15	Ru1	C14	C18	-118.1(3)
C16	Ru1	C14	C13	102.09(16)
C16	Ru1	C14	C15	-27.85(14)
C16	Ru1	C14	C18	-145.9(3)
S1	Ru1	C15	C14	-101.51(13)
S1	Ru1	C15	C16	125.34(14)
S2	Ru1	C15	C14	173.58(11)
S2	Ru1	C15	C16	40.44(16)
P1	Ru1	C15	C14	-17.8(2)
P1	Ru1	C15	C16	-150.96(13)
C11	Ru1	C15	C14	103.99(16)
C11	Ru1	C15	C16	-29.16(15)
C12	Ru1	C15	C14	67.21(15)
C12	Ru1	C15	C16	-65.94(15)
C13	Ru1	C15	C14	30.10(14)
C13	Ru1	C15	C16	-103.05(16)
C14	Ru1	C15	C16	-133.1(2)
C16	Ru1	C15	C14	133.1(2)
S1	Ru1	C16	C11	167.99(13)
S1	Ru1	C16	C15	-59.78(15)
S2	Ru1	C16	C11	80.53(14)
S2	Ru1	C16	C15	-147.25(14)
P1	Ru1	C16	C11	-13.6(3)
P1	Ru1	C16	C15	118.6(2)
C11	Ru1	C16	C15	132.2(2)
C12	Ru1	C16	C11	-30.25(15)
C12	Ru1	C16	C15	101.98(17)
C13	Ru1	C16	C11	-67.27(16)
C13	Ru1	C16	C15	64.96(15)
C14	Ru1	C16	C11	-103.77(16)
C14	Ru1	C16	C15	28.45(14)
C15	Ru1	C16	C11	-132.2(2)
Ru1	S1	C1	C2	-176.62(19)
Ru1	S1	C1	C6	4.9(2)
Ru1	S2	C6	C1	-2.4(2)
Ru1	S2	C6	C5	177.88(19)
Ru1	P1	C21	C22	95.2(2)
Ru1	P1	C21	C26	-81.8(2)
C31	P1	C21	C22	-134.8(2)
C31	P1	C21	C26	48.1(2)
C41	P1	C21	C22	-27.6(3)
C41	P1	C21	C26	155.4(2)
Ru1	P1	C31	C32	-175.81(19)
Ru1	P1	C31	C36	3.9(3)
C21	P1	C31	C32	52.7(2)
C21	P1	C31	C36	-127.6(2)
C41	P1	C31	C32	-55.7(2)
C41	P1	C31	C36	124.0(2)
Ru1	P1	C41	C42	113.7(2)
Ru1	P1	C41	C46	-58.6(2)
C21	P1	C41	C42	-116.9(2)
C21	P1	C41	C46	70.8(2)
C31	P1	C41	C42	-11.9(2)
C31	P1	C41	C46	175.9(2)
S1	C1	C2	Cl1	0.7(3)
S1	C1	C2	C3	-179.2(2)
C6	C1	C2	Cl1	179.29(19)
C6	C1	C2	C3	-0.6(4)
S1	C1	C6	S2	-1.8(3)
S1	C1	C6	C5	177.97(19)
C2	C1	C6	S2	179.68(19)
C2	C1	C6	C5	-0.6(4)
Cl1	C2	C3	C4	-178.3(2)
C1	C2	C3	C4	1.6(4)
C2	C3	C4	C5	-1.4(4)
C3	C4	C5	Cl2	-179.2(2)
C3	C4	C5	C6	0.2(4)
Cl2	C5	C6	S2	-0.1(3)
Cl2	C5	C6	C1	-179.86(19)
C4	C5	C6	S2	-179.5(2)
C4	C5	C6	C1	0.8(4)
Ru1	C11	C12	C13	54.1(2)
C16	C11	C12	Ru1	-56.0(2)
C16	C11	C12	C13	-1.9(4)
C17	C11	C12	Ru1	126.4(2)
C17	C11	C12	C13	-179.5(2)
Ru1	C11	C16	C15	-55.0(2)
C12	C11	C16	Ru1	55.6(2)
C12	C11	C16	C15	0.7(4)
C17	C11	C16	Ru1	-126.7(2)
C17	C11	C16	C15	178.4(2)
Ru1	C12	C13	C14	57.9(2)
C11	C12	C13	Ru1	-55.1(2)
C11	C12	C13	C14	2.8(4)
Ru1	C13	C14	C15	54.70(19)
Ru1	C13	C14	C18	-131.8(2)
C12	C13	C14	Ru1	-57.2(2)
C12	C13	C14	C15	-2.5(3)

Table S9. Torsional Angles (continued).

Atom1	Atom2	Atom3	Atom4	Angle	Atom1	Atom2	Atom3	Atom4	Angle
C12	C13	C14	C18	171.1(2)	P1	C21	C22	C23	-176.5(2)
Ru1	C14	C15	C16	55.1(2)	C26	C21	C22	C23	0.5(4)
C13	C14	C15	Ru1	-53.84(19)	P1	C21	C26	C25	176.6(2)
C13	C14	C15	C16	1.2(3)	C22	C21	C26	C25	-0.6(4)
C18	C14	C15	Ru1	132.9(2)	C21	C22	C23	C24	0.0(5)
C18	C14	C15	C16	-172.1(2)	C22	C23	C24	C25	-0.4(5)
Ru1	C14	C18	C19	56.2(4)	C23	C24	C25	C26	0.3(5)
Ru1	C14	C18	C20	-179.1(2)	C24	C25	C26	C21	0.2(4)
C13	C14	C18	C19	146.6(3)	P1	C31	C32	C33	177.1(2)
C13	C14	C18	C20	-88.7(3)	C36	C31	C32	C33	-2.6(4)
C15	C14	C18	C19	-40.2(4)	P1	C31	C36	C35	-177.8(2)
C15	C14	C18	C20	84.5(3)	C32	C31	C36	C35	1.9(4)
Ru1	C15	C16	C11	54.7(2)	C31	C32	C33	C34	1.4(5)
C14	C15	C16	Ru1	-55.1(2)	C32	C33	C34	C35	0.5(5)
C14	C15	C16	C11	-0.4(4)					

Table S10. Least-Squares Planes.

Dihedral angle between planes 1 and 2: 57.34(6)°

Plane	Coefficients[a]				Defining Atoms with Deviations (Å)[b]			
1	6.787(9)	2.692(11)	11.603(10)	2.228(4)				
					C11	0.0034(17)	C12	-0.0109(17)
					C13	0.0131(17)	C14	-0.0077(16)
					C15	0.0003(17)	C16	0.0018(17)
					Ru1	1.7690(10)	C17	-0.032(4)
					C18	-0.186(4)		
2	-0.671(6)	6.811(4)	13.705(4)	2.347(3)				
					Ru1	0.0362(5)	S1	-0.0426(8)
					S2	-0.0353(9)	C1	0.0293(15)
					C6	0.0123(15)		

[a]Coefficients are for the form *ax*+*by*+*cz* = *d* where *x*, *y* and *z* are crystallographic coordinates.
[b]Underlined atoms were not included in the definition of the plane.

Table S11. Anisotropic Displacement Parameters (U_{ij}, Å^2).

Atom	U_{11}	U_{22}	U_{33}	U_{23}	U_{13}	U_{12}
Ru1	0.01931(9)	0.02315(10)	0.02535(10)	-0.00939(7)	0.00526(6)	-0.00865(7)
Cl1	0.0292(3)	0.0585(5)	0.0601(5)	-0.0143(4)	0.0009(3)	-0.0224(3)
Cl2	0.0586(5)	0.0393(4)	0.0585(5)	0.0084(4)	0.0021(4)	-0.0122(4)
S1	0.0200(3)	0.0294(3)	0.0283(3)	-0.0087(2)	0.0038(2)	-0.0098(2)
S2	0.0289(3)	0.0257(3)	0.0331(3)	-0.0059(2)	0.0047(2)	-0.0101(3)
P1	0.0233(3)	0.0254(3)	0.0245(3)	-0.0095(2)	0.0062(2)	-0.0103(2)
C1	0.0236(11)	0.0340(13)	0.0254(12)	-0.0148(10)	0.0032(9)	-0.0080(10)
C2	0.0272(12)	0.0430(16)	0.0352(14)	-0.0175(12)	0.0055(10)	-0.0109(12)
C3	0.0262(13)	0.0490(18)	0.0427(16)	-0.0192(14)	-0.0023(11)	-0.0035(13)
C4	0.0368(15)	0.0413(16)	0.0339(15)	-0.0101(13)	-0.0036(12)	0.0029(13)
C5	0.0406(15)	0.0345(15)	0.0295(13)	-0.0088(11)	0.0065(11)	-0.0072(12)
C6	0.0280(12)	0.0303(13)	0.0256(12)	-0.0110(10)	0.0037(10)	-0.0055(11)
C11	0.0276(12)	0.0316(13)	0.0354(14)	-0.0097(11)	-0.0010(10)	-0.0146(11)
C12	0.0226(11)	0.0385(14)	0.0321(13)	-0.0107(11)	0.0021(10)	-0.0148(11)
C13	0.0194(11)	0.0263(12)	0.0335(13)	-0.0100(10)	-0.0027(9)	-0.0033(10)
C14	0.0248(11)	0.0280(13)	0.0266(12)	-0.0046(10)	-0.0029(9)	-0.0114(10)
C15	0.0254(12)	0.0370(14)	0.0258(12)	-0.0107(11)	0.0036(9)	-0.0124(11)
C16	0.0288(12)	0.0328(13)	0.0302(13)	-0.0185(11)	0.0001(10)	-0.0097(11)
C17	0.0448(16)	0.0398(16)	0.0535(18)	-0.0125(14)	0.0033(14)	-0.0259(14)
C18	0.0365(14)	0.0344(14)	0.0372(15)	-0.0052(12)	-0.0017(11)	-0.0180(12)
C19	0.0397(16)	0.0504(19)	0.060(2)	0.0035(16)	-0.0027(15)	-0.0242(15)
C20	0.0508(18)	0.0380(16)	0.0452(17)	0.0011(13)	-0.0106(14)	-0.0206(14)
C21	0.0269(12)	0.0335(14)	0.0244(12)	-0.0104(10)	0.0053(9)	-0.0096(11)
C22	0.0387(15)	0.0437(16)	0.0351(14)	-0.0090(12)	0.0020(11)	-0.0213(13)
C23	0.0470(17)	0.066(2)	0.0428(17)	-0.0101(15)	-0.0031(14)	-0.0338(17)
C24	0.0362(15)	0.069(2)	0.0387(16)	-0.0090(15)	-0.0045(12)	-0.0206(16)
C25	0.0372(15)	0.0519(18)	0.0292(14)	-0.0035(13)	-0.0013(11)	-0.0098(14)
C26	0.0349(14)	0.0370(15)	0.0304(13)	-0.0083(11)	0.0063(11)	-0.0136(12)
C31	0.0274(12)	0.0319(13)	0.0266(12)	-0.0062(10)	0.0068(10)	-0.0112(11)
C32	0.0384(14)	0.0466(17)	0.0321(14)	-0.0153(12)	0.0084(11)	-0.0183(13)
C33	0.0440(16)	0.061(2)	0.0314(15)	-0.0172(14)	0.0163(12)	-0.0158(16)
C34	0.0387(15)	0.060(2)	0.0383(16)	-0.0032(14)	0.0156(13)	-0.0213(15)
C35	0.0377(15)	0.0457(17)	0.0443(16)	-0.0041(13)	0.0099(12)	-0.0232(14)
C36	0.0341(13)	0.0345(14)	0.0342(14)	-0.0072(11)	0.0088(11)	-0.0154(12)
C41	0.0284(12)	0.0269(12)	0.0284(12)	-0.0129(10)	0.0024(10)	-0.0108(10)
C42	0.0304(13)	0.0339(14)	0.0359(14)	-0.0150(11)	0.0041(10)	-0.0139(11)
C43	0.0319(13)	0.0344(15)	0.0508(17)	-0.0198(13)	0.0012(12)	-0.0088(12)
C44	0.0415(15)	0.0260(14)	0.0526(17)	-0.0122(12)	-0.0052(13)	-0.0111(12)
C45	0.0424(15)	0.0315(14)	0.0424(15)	-0.0069(12)	0.0000(12)	-0.0197(13)
C46	0.0295(12)	0.0301(13)	0.0337(13)	-0.0120(11)	0.0047(10)	-0.0128(11)
O1S	0.0630(17)	0.0663(19)	0.087(2)	-0.0203(16)	0.0185(15)	-0.0068(14)
C1S	0.117(4)	0.072(3)	0.081(3)	-0.022(3)	0.038(3)	-0.036(3)
C2S	0.110(4)	0.090(4)	0.155(6)	-0.060(4)	0.078(4)	-0.047(4)
C3S	0.105(4)	0.070(3)	0.203(7)	-0.048(4)	0.082(5)	-0.034(3)
C4S	0.082(3)	0.075(3)	0.118(4)	-0.030(3)	0.043(3)	-0.025(3)

The form of the anisotropic displacement parameter is: $\exp[-2\pi^2(h^2a^{*2}U_{11} + k^2b^{*2}U_{22} + l^2c^{*2}U_{33} + 2klb^*c^*U_{23} + 2hla^*c^*U_{13} + 2hka^*b^*U_{12})]$

Table S12. Derived Atomic Coordinates and Displacement Parameters for Hydrogen Atoms.

Atom	*x*	*y*	*z*	U_{eq}, Å^2
H3	0.962771	-0.108332	0.284428	0.051
H4	0.893456	-0.269217	0.354946	0.054
H12	0.078810	0.236381	0.102675	0.037
H13	0.113144	0.429293	0.040933	0.034
H15	0.417915	0.221732	-0.088410	0.035
H16	0.388249	0.029142	-0.027002	0.036
H17A	0.285624	-0.081115	0.102210	0.065
H17B	0.167989	-0.025224	0.021846	0.065
H17C	0.139052	0.006407	0.118132	0.065
H18	0.224287	0.541101	-0.050085	0.044
H19A	0.435101	0.416443	-0.145321	0.079
H19B	0.453458	0.425598	-0.043439	0.079
H19C	0.393038	0.557717	-0.128814	0.079
H20A	0.177130	0.627563	-0.213010	0.071
H20B	0.084577	0.560286	-0.170164	0.071
H20C	0.205915	0.483584	-0.219473	0.071
H22	0.487659	0.389936	0.254538	0.046
H23	0.674003	0.320525	0.355626	0.060
H24	0.757689	0.115388	0.466836	0.060
H25	0.653901	-0.021422	0.478707	0.054
H26	0.466330	0.046518	0.378104	0.042
H32	0.219838	0.333687	0.388362	0.046
H33	0.074474	0.292838	0.486737	0.057
H34	-0.019635	0.158559	0.465358	0.058
H35	0.033143	0.062206	0.345871	0.051
H36	0.175876	0.106645	0.244641	0.042
H42	0.065303	0.484396	0.239962	0.039
H43	-0.038055	0.712751	0.170175	0.047
H44	0.071711	0.818703	0.077514	0.049
H45	0.284180	0.695348	0.049467	0.046
H46	0.383943	0.465402	0.111977	0.037
H1SA	0.314927	0.814188	0.371688	0.111
H1SB	0.225198	0.825499	0.285716	0.111
H2SA	0.353503	0.626384	0.286120	0.135
H2SB	0.453094	0.623673	0.362127	0.135
H3SA	0.321727	0.480904	0.391296	0.152
H3SB	0.400822	0.498229	0.469989	0.152
H4SA	0.221512	0.598860	0.514418	0.114
H4SB	0.142526	0.599855	0.427000	0.114

S5.2 X-ray Crystallographic Analysis of Ru(S-S)(PPh_3)(H_2IPr) Ru-5.

Table S13. Crystallographic Experimental Details

A. Crystal Data	
formula	$C_{55}H_{65}Cl_2N_2OPRuS_2$
formula weight	1037.15
crystal colour and habit[a]	purple plate
crystal dimensions (mm)	0.27 × 0.08 × 0.02
crystal system	orthorhombic
space group	$P2_12_12_1$ (No. 19)
unit cell parameters[b]	
a (Å)	12.9859(3)
b (Å)	17.0051(5)
c (Å)	23.7009(7)
V (Å^3)	5233.8(2)
Z	4
ρ_{calcd} (g cm^{-3})	1.316
μ (mm^{-1})	4.695
B. Data Collection and Refinement Conditions	
diffractometer	Bruker D8 Venture/PHOTON III[c]
radiation (λ [Å])	Cu K$_\alpha$ (1.54178) (microfocus source)
temperature (°C)	–100
scan type	ω and ϕ scans (1.0°) (θ-dep. exp. from 5-20 s)
data collection 2θ limit (°)	144.34
total data collected	69351 ($-16 \le h \le 16$, $-18 \le k \le 20$, $-28 \le l \le 29$)
independent reflections	10281 (R_{int} = 0.0517)
number of observed reflections (*NO*)	9863 [$F_o^2 \ge 2\sigma(F_o^2)$]
structure solution method	intrinsic phasing (*SHELXT-2018/2*)[17]
refinement method	full-matrix least-squares on F^2 (*SHELXL–2019/1*)[17]
absorption correction method	Gaussian integration (face-indexed)
range of transmission factors	1.0000–0.4178
data/restraints/parameters	10281 / 90[d]/ 635
Flack absolute structure parameter[e]	0.012(2)
goodness-of-fit (S)[f] [all data]	1.030
final R indices[g]	
R_1 [$F_o^2 \ge 2\sigma(F_o^2)$]	0.0244
wR_2 [all data]	0.0631
largest difference peak and hole	0.699 and –0.398 e Å^{-3}

[a]Obtained by recrystallization from Et_2O. [b]Obtained from least-squares refinement of 9805 reflections with 8.56° < 2σ < 144.28°. [c]Programs for diffractometer operation, data collection, data reduction and absorption correction were those supplied by Bruker. [d]For refinement of the disordered Et_2O molecule, see ref [18]. [e]Flack, H. D. *Acta Crystallogr.* **1983**, *A39*, 876–881; Flack, H. D.; Bernardinelli, G. *Acta Crystallogr.* **1999**, *A55*, 908–915; Flack, H. D.; Bernardinelli, G. *J. Appl. Cryst.* **2000**, *33*, 1143–1148. The Flack parameter will refine to a value near 0 if the structure is in the correct configuration, and a value near 1 for the inverted configuration. [f]$S = [\Sigma w(F_o^2 - F_c^2)^2/(n - p)]^{1/2}$ (n = number of data; p = number of parameters varied; $w = [\sigma^2 F_o^2) + (0.0347P)^2 + 0.7093P]^{-1}$ where $P = [\mathrm{Max}(F_o^2, 0) + 2F_c^2]/3$). [g]$R_1 = \Sigma||F_o| - |F_c||/\Sigma|F_o|$; $wR_2 = [\Sigma w(F_o^2 - F_c^2)^2/\Sigma w(F_o^4)]^{1/2}$.

Table S14. Atomic Coordinates and Equivalent Isotropic Displacement Parameters.

(a) $Ru(S_2C_6H_2Cl_2)(PPh_3)(H_2IPr)$ atoms.

Atom	*x*	*y*	*z*	U_{eq}, Å^2
Ru1	0.64585(2)	0.34354(2)	0.72403(2)	0.02277(6)*
Cl1	0.63657(9)	0.21647(6)	0.51714(3)	0.0511(2)*
Cl2	0.81078(6)	0.04832(5)	0.73003(4)	0.04129(18)*
S1	0.69695(6)	0.21455(4)	0.74447(3)	0.02826(15)*
S2	0.62530(5)	0.30462(4)	0.63486(3)	0.02764(15)*
P1	0.81150(5)	0.38076(4)	0.72603(3)	0.02493(14)*
N1	0.55083(18)	0.48230(14)	0.76267(10)	0.0253(5)*
N2	0.50616(19)	0.48301(15)	0.67431(11)	0.0261(5)*
C1	0.7125(2)	0.17082(18)	0.67849(13)	0.0270(6)*
C2	0.6766(2)	0.20930(18)	0.63000(13)	0.0269(6)*
C3	0.6815(2)	0.1702(2)	0.57784(14)	0.0341(7)*
C4	0.7201(3)	0.0951(2)	0.57309(15)	0.0368(7)*
C5	0.7595(2)	0.0584(2)	0.62063(15)	0.0353(7)*
C6	0.7575(2)	0.0960(2)	0.67178(15)	0.0321(7)*
C11	0.5654(2)	0.44618(16)	0.71229(12)	0.0229(6)*
C12	0.4661(3)	0.54059(19)	0.76180(13)	0.0319(7)*
C13	0.4495(3)	0.55037(18)	0.69840(14)	0.0323(7)*
C21	0.5842(2)	0.43561(18)	0.80985(13)	0.0282(6)*
C22	0.6468(3)	0.46970(19)	0.85249(12)	0.0306(6)*
C23	0.6855(3)	0.4193(2)	0.89319(15)	0.0390(8)*
C24	0.6631(3)	0.3392(2)	0.89282(14)	0.0428(8)*
C25	0.5940(3)	0.3091(2)	0.85475(16)	0.0395(8)*
C26	0.5482(2)	0.35715(19)	0.81294(14)	0.0322(7)*
C27	0.6594(3)	0.5577(2)	0.85701(14)	0.0349(7)*
C28	0.5747(3)	0.5902(3)	0.8957(2)	0.0532(10)*
C29	0.7657(3)	0.5833(2)	0.87862(19)	0.0482(9)*
C30	0.4402(2)	0.3361(2)	0.79109(14)	0.0344(7)*
C31	0.3609(3)	0.3609(2)	0.8359(2)	0.0571(11)*
C32	0.4238(3)	0.2495(2)	0.7775(2)	0.0527(10)*
C41	0.4910(2)	0.46411(19)	0.61528(13)	0.0287(6)*
C42	0.5571(3)	0.4956(2)	0.57467(14)	0.0357(7)*
C43	0.5379(3)	0.4775(3)	0.51820(16)	0.0450(8)*
C44	0.4560(3)	0.4300(2)	0.50301(16)	0.0469(9)*
C45	0.3902(3)	0.4011(2)	0.54368(17)	0.0434(8)*
C46	0.4049(3)	0.41887(19)	0.60065(15)	0.0338(7)*
C47	0.6463(3)	0.5497(3)	0.58957(14)	0.0472(9)*
C48	0.6274(4)	0.6335(3)	0.5699(2)	0.0705(13)*
C49	0.7469(3)	0.5182(4)	0.5657(2)	0.0656(13)*
C50	0.3269(3)	0.3878(2)	0.64325(18)	0.0421(8)*
C51	0.2212(3)	0.4256(3)	0.6350(2)	0.0588(11)*
C52	0.3183(4)	0.2983(3)	0.6408(3)	0.0653(13)*
C61	0.8779(2)	0.3771(2)	0.79530(13)	0.0321(7)*
C62	0.8678(3)	0.3111(2)	0.82949(14)	0.0395(8)*
C63	0.9189(3)	0.3058(3)	0.88116(16)	0.0480(9)*
C64	0.9800(3)	0.3673(3)	0.89925(16)	0.0537(11)*
C65	0.9899(3)	0.4340(3)	0.86586(18)	0.0526(10)*
C66	0.9401(3)	0.4383(2)	0.81425(16)	0.0431(8)*

Table S14. Atomic Coordinates and Displacement Parameters (continued).

Atom	x	y	z	U_{eq}, Å^2
C71	0.8520(2)	0.47917(18)	0.70248(12)	0.0295(6)*
C72	0.7925(2)	0.54371(19)	0.71693(14)	0.0336(7)*
C73	0.8264(3)	0.6194(2)	0.70582(16)	0.0407(8)*
C74	0.9213(3)	0.6309(2)	0.67974(16)	0.0422(8)*
C75	0.9805(3)	0.5673(2)	0.66485(16)	0.0430(8)*
C76	0.9467(3)	0.4917(2)	0.67607(15)	0.0379(7)*
C81	0.8928(2)	0.31656(19)	0.68196(14)	0.0298(6)*
C82	0.8809(3)	0.3187(2)	0.62366(15)	0.0389(8)*
C83	0.9389(3)	0.2708(3)	0.58887(16)	0.0446(9)*
C84	1.0091(3)	0.2192(3)	0.61179(17)	0.0464(9)*
C85	1.0206(3)	0.2154(3)	0.66942(18)	0.0518(10)*
C86	0.9632(3)	0.2640(2)	0.70461(16)	0.0422(8)*

(b) Et_2O solvent atoms

Atom	x	y	z	U_{eq}, Å^2
O1S^a	0.8770(6)	0.1424(3)	0.9674(3)	0.071(2)*
C1S^a	0.7914(14)	0.0912(8)	0.9772(8)	0.093(4)*
C2S^a	0.6995(15)	0.1243(14)	0.9479(13)	0.105(7)*
C3S^a	0.9744(10)	0.1167(7)	0.9892(4)	0.082(3)*
C4S^a	1.0531(8)	0.1748(7)	0.9784(4)	0.089(3)*
O2S^b	0.8367(12)	0.0898(17)	0.9704(10)	0.129(10)*
C5S^b	0.7570(16)	0.1390(15)	0.9912(9)	0.106(6)*
C6S^b	0.670(2)	0.134(3)	0.9529(16)	0.090(9)*
C7S^b	0.9247(17)	0.088(2)	1.0057(11)	0.158(12)*
C8S^b	0.989(2)	0.025(2)	0.9840(11)	0.191(16)*

Anisotropically-refined atoms are marked with an asterisk (*). The form of the anisotropic displacement parameter is: $\exp[-2\pi^2(h^2a^{*2}U_{11} + k^2b^{*2}U_{22} + l^2c^{*2}U_{33} + 2klb^*c^*U_{23} + 2hla^*c^*U_{13} + 2hka^*b^*U_{12})]$. Refined occupancies of 0.614(10)^a and 0.386(10)^b.

Table S15. Selected Interatomic Distances (Å).

(a) Within the $Ru(S_2C_6H_2Cl_2)(PPh_3)(H_2IPr)$ atoms molecule

Atom1	Atom2	Distance	Atom1	Atom2	Distance
Ru1	S1	2.3423(8)	C27	C29	1.535(5)
Ru1	S2	2.2307(7)	C30	C31	1.539(5)
Ru1	P1	2.2428(7)	C30	C32	1.522(5)
Ru1	C11	2.053(3)	C41	C42	1.397(5)
Ru1	C26	2.470(3)	C41	C46	1.401(5)
Cl1	C3	1.741(4)	C42	C43	1.396(5)
Cl2	C6	1.744(4)	C42	C47	1.520(5)
S1	C1	1.743(3)	C43	C44	1.383(6)
S2	C2	1.756(3)	C44	C45	1.378(6)
P1	C61	1.856(3)	C45	C46	1.397(5)
P1	C71	1.841(3)	C46	C50	1.524(5)
P1	C81	1.843(3)	C47	C48	1.519(7)
N1	C11	1.356(4)	C47	C49	1.522(7)
N1	C12	1.481(4)	C50	C51	1.529(6)
N1	C21	1.438(4)	C50	C52	1.528(6)
N2	C11	1.340(4)	C61	C62	1.391(5)
N2	C13	1.477(4)	C61	C66	1.391(5)
N2	C41	1.449(4)	C62	C63	1.396(5)
C1	C2	1.402(4)	C63	C64	1.382(6)
C1	C6	1.409(4)	C64	C65	1.388(7)
C2	C3	1.405(4)	C65	C66	1.386(6)
C3	C4	1.376(5)	C71	C72	1.385(5)
C4	C5	1.386(5)	C71	C76	1.397(5)
C5	C6	1.371(5)	C72	C73	1.386(5)
C12	C13	1.527(4)	C73	C74	1.392(5)
C21	C22	1.421(5)	C74	C75	1.372(6)
C21	C26	1.415(5)	C75	C76	1.384(6)
C22	C23	1.385(5)	C81	C82	1.391(5)
C22	C27	1.509(5)	C81	C86	1.386(5)
C23	C24	1.394(6)	C82	C83	1.381(5)
C24	C25	1.371(5)	C83	C84	1.377(6)
C25	C26	1.415(5)	C84	C85	1.375(6)
C26	C30	1.538(5)	C85	C86	1.391(5)
C27	C28	1.534(5)			

(b) Within the solvent Et_2O molecules

Atom1	Atom2	Distance	Atom1	Atom2	Distance
O1S	C1S	1.431(14)†	C1S	C2S	1.491(19)†
O1S	C3S	1.435(12)†	C3S	C4S	1.446(16)†
O2S	C5S	1.42(2)†	C5S	C6S	1.45(2)†
O2S	C7S	1.416(18)†	C7S	C8S	1.46(2)†

†Restrained during refinement.

Table S16. Selected Interatomic Angles (°)

(a) Within the Ru($S_2C_6H_2Cl_2$)(PPh_3)(H_2IPr) atoms molecule

Atom1	Atom2	Atom3	Angle	Atom1	Atom2	Atom3	Angle
S1	Ru1	S2	87.25(3)	C21	C22	C27	121.2(3)
S1	Ru1	P1	89.33(3)	C23	C22	C27	121.6(3)
S1	Ru1	C11	165.54(8)	C22	C23	C24	121.6(3)
S1	Ru1	C26	93.25(8)	C23	C24	C25	120.4(3)
S2	Ru1	P1	102.62(3)	C24	C25	C26	121.4(3)
S2	Ru1	C11	93.61(8)	Ru1	C26	C21	82.82(18)
S2	Ru1	C26	140.77(8)	Ru1	C26	C25	109.1(2)
P1	Ru1	C11	104.53(8)	Ru1	C26	C30	99.2(2)
P1	Ru1	C26	116.60(8)	C21	C26	C25	116.2(3)
C11	Ru1	C26	76.99(11)	C21	C26	C30	120.2(3)
Ru1	S1	C1	104.29(11)	C25	C26	C30	119.0(3)
Ru1	S2	C2	106.90(11)	C22	C27	C28	108.7(3)
Ru1	P1	C61	117.07(11)	C22	C27	C29	113.8(3)
Ru1	P1	C71	121.62(11)	C28	C27	C29	110.1(3)
Ru1	P1	C81	111.73(11)	C26	C30	C31	108.3(3)
C61	P1	C71	99.55(15)	C26	C30	C32	115.1(3)
C61	P1	C81	102.43(14)	C31	C30	C32	108.6(3)
C71	P1	C81	101.70(15)	N2	C41	C42	119.8(3)
C11	N1	C12	113.3(2)	N2	C41	C46	118.0(3)
C11	N1	C21	113.1(2)	C42	C41	C46	122.1(3)
C12	N1	C21	127.1(2)	C41	C42	C43	117.8(3)
C11	N2	C13	112.9(2)	C41	C42	C47	122.7(3)
C11	N2	C41	128.5(2)	C43	C42	C47	119.5(3)
C13	N2	C41	118.5(2)	C42	C43	C44	121.1(4)
S1	C1	C2	119.8(2)	C43	C44	C45	120.2(3)
S1	C1	C6	122.3(2)	C44	C45	C46	121.0(3)
C2	C1	C6	117.8(3)	C41	C46	C45	117.8(3)
S2	C2	C1	120.2(2)	C41	C46	C50	123.8(3)
S2	C2	C3	120.8(2)	C45	C46	C50	118.4(3)
C1	C2	C3	119.0(3)	C42	C47	C48	111.9(4)
Cl1	C3	C2	119.8(3)	C42	C47	C49	110.8(4)
Cl1	C3	C4	118.3(3)				
C2	C3	C4	121.9(3)				
C3	C4	C5	119.1(3)				
C4	C5	C6	120.1(3)				
Cl2	C6	C1	119.7(3)				
Cl2	C6	C5	118.4(3)				
C1	C6	C5	122.0(3)				
Ru1	C11	N1	109.68(19)				
Ru1	C11	N2	141.3(2)				
N1	C11	N2	107.4(2)				
N1	C12	C13	101.1(2)				
N2	C13	C12	103.0(2)				
N1	C21	C22	120.0(3)				
N1	C21	C26	117.5(3)				
C22	C21	C26	122.5(3)				
C21	C22	C23	116.8(3)				

Table S16. Selected Interatomic Angles (continued).

Atom1	Atom2	Atom3	Angle	Atom1	Atom2	Atom3	Angle
C48	C47	C49	110.7(4)	C72	C71	C76	118.7(3)
C46	C50	C51	111.5(3)	C71	C72	C73	120.8(3)
C46	C50	C52	111.6(4)	C72	C73	C74	119.7(3)
C51	C50	C52	110.4(4)	C73	C74	C75	119.9(3)
P1	C61	C62	119.9(3)	C74	C75	C76	120.3(3)
P1	C61	C66	122.0(3)	C71	C76	C75	120.5(3)
C62	C61	C66	118.0(3)	P1	C81	C82	119.0(2)
C61	C62	C63	121.2(4)	P1	C81	C86	122.7(3)
C62	C63	C64	119.8(4)	C82	C81	C86	118.3(3)
C63	C64	C65	119.6(3)	C81	C82	C83	121.2(3)
C64	C65	C66	120.2(4)	C82	C83	C84	120.0(4)
C61	C66	C65	121.1(4)	C83	C84	C85	119.6(3)
P1	C71	C72	119.1(2)	C84	C85	C86	120.6(4)
P1	C71	C76	121.8(3)	C81	C86	C85	120.3(3)

(b) Within the solvent Et_2O molecules

Atom1	Atom2	Atom3	Angle	Atom1	Atom2	Atom3	Angle
C1S	O1S	C3S	116.1(9)	C5S	O2S	C7S	113(2)
O1S	C1S	C2S	108.5(13)	O2S	C5S	C6S	108(2)
O1S	C3S	C4S	110.5(8)	O2S	C7S	C8S	106(2)

Table S17. Torsional Angles (°)

Atom1	Atom2	Atom3	Atom4	Angle	Atom1	Atom2	Atom3	Atom4	Angle
Ru1	S1	C1	C2	-11.6(3)	C2	C1	C6	Cl2	176.0(2)
Ru1	S1	C1	C6	170.5(2)	C2	C1	C6	C5	-5.1(5)
Ru1	S2	C2	C1	4.8(3)	S2	C2	C3	Cl1	0.1(4)
Ru1	S2	C2	C3	-175.7(2)	S2	C2	C3	C4	-179.2(3)
Ru1	P1	C61	C62	46.3(3)	C1	C2	C3	Cl1	179.6(2)
Ru1	P1	C61	C66	-134.9(3)	C1	C2	C3	C4	0.3(5)
C71	P1	C61	C62	179.4(3)	Cl1	C3	C4	C5	177.8(3)
C71	P1	C61	C66	-1.9(3)	C2	C3	C4	C5	-3.0(5)
C81	P1	C61	C62	-76.2(3)	C3	C4	C5	C6	1.5(5)
C81	P1	C61	C66	102.5(3)	C4	C5	C6	Cl2	-178.5(3)
Ru1	P1	C71	C72	41.1(3)	C4	C5	C6	C1	2.6(5)
Ru1	P1	C71	C76	-146.3(2)	N1	C12	C13	N2	14.1(3)
C61	P1	C71	C72	-89.1(3)	N1	C21	C22	C23	-172.9(3)
C61	P1	C71	C76	83.5(3)	N1	C21	C22	C27	14.3(4)
C81	P1	C71	C72	166.0(3)	C26	C21	C22	C23	10.1(5)
C81	P1	C71	C76	-21.5(3)	C26	C21	C22	C27	-162.6(3)
Ru1	P1	C81	C82	68.4(3)	N1	C21	C26	Ru1	63.0(2)
Ru1	P1	C81	C86	-109.3(3)	N1	C21	C26	C25	170.8(3)
C61	P1	C81	C82	-165.5(3)	N1	C21	C26	C30	-33.4(4)
C61	P1	C81	C86	16.8(3)	C22	C21	C26	Ru1	-120.0(3)
C71	P1	C81	C82	-62.8(3)	C22	C21	C26	C25	-12.2(5)
C71	P1	C81	C86	119.4(3)	C22	C21	C26	C30	143.6(3)
C12	N1	C11	Ru1	-160.6(2)	C21	C22	C23	C24	-0.8(5)
C12	N1	C11	N2	8.4(3)	C27	C22	C23	C24	171.9(3)
C21	N1	C11	Ru1	-6.7(3)	C21	C22	C27	C28	89.6(4)
C21	N1	C11	N2	162.3(2)	C21	C22	C27	C29	-147.3(3)
C11	N1	C12	C13	-14.5(3)	C23	C22	C27	C28	-82.8(4)
C21	N1	C12	C13	-164.0(3)	C23	C22	C27	C29	40.3(5)
C11	N1	C21	C22	132.2(3)	C22	C23	C24	C25	-5.9(6)
C11	N1	C21	C26	-50.7(4)	C23	C24	C25	C26	3.7(6)
C12	N1	C21	C22	-78.3(4)	C24	C25	C26	Ru1	96.4(4)
C12	N1	C21	C26	98.8(4)	C24	C25	C26	C21	5.1(5)
C13	N2	C11	Ru1	165.4(3)	C24	C25	C26	C30	-151.0(3)
C13	N2	C11	N1	2.1(3)	Ru1	C26	C30	C31	-165.7(2)
C41	N2	C11	Ru1	-13.5(5)	Ru1	C26	C30	C32	72.6(3)
C41	N2	C11	N1	-176.7(3)	C21	C26	C30	C31	-78.7(4)
C11	N2	C13	C12	-10.9(4)	C21	C26	C30	C32	159.6(3)
C41	N2	C13	C12	168.1(3)	C25	C26	C30	C31	76.4(4)
C11	N2	C41	C42	-88.3(4)	C25	C26	C30	C32	-45.3(5)
C11	N2	C41	C46	96.3(4)	N2	C41	C42	C43	-178.3(3)
C13	N2	C41	C42	92.9(4)	N2	C41	C42	C47	0.2(5)
C13	N2	C41	C46	-82.5(4)	C46	C41	C42	C43	-3.1(5)
S1	C1	C2	S2	5.1(4)	C46	C41	C42	C47	175.4(3)
S1	C1	C2	C3	-174.4(2)	N2	C41	C46	C45	179.7(3)
C6	C1	C2	S2	-176.9(2)	N2	C41	C46	C50	-0.6(5)
C6	C1	C2	C3	3.6(4)	C42	C41	C46	C45	4.4(5)
S1	C1	C6	Cl2	-6.0(4)	C42	C41	C46	C50	-175.9(3)
S1	C1	C6	C5	172.8(3)	C41	C42	C43	C44	0.1(6)

Table S17. Torsional Angles (continued).

Atom1	Atom2	Atom3	Atom4	Angle
C47	C42	C43	C44	-178.4(4)
C41	C42	C47	C48	-110.3(4)
C41	C42	C47	C49	125.6(4)
C43	C42	C47	C48	68.1(5)
C43	C42	C47	C49	-56.0(5)
C42	C43	C44	C45	1.5(6)
C43	C44	C45	C46	-0.1(6)
C44	C45	C46	C41	-2.8(5)
C44	C45	C46	C50	177.5(3)
C41	C46	C50	C51	114.0(4)
C41	C46	C50	C52	-122.1(4)
C45	C46	C50	C51	-66.3(5)
C45	C46	C50	C52	57.6(5)

Atom1	Atom2	Atom3	Atom4	Angle
P1	C61	C62	C63	178.4(3)
C66	C61	C62	C63	-0.4(5)
P1	C61	C66	C65	-179.3(3)
C62	C61	C66	C65	-0.5(5)
C61	C62	C63	C64	0.6(6)
C62	C63	C64	C65	0.1(6)
C63	C64	C65	C66	-1.0(7)
C64	C65	C66	C61	1.3(6)
P1	C71	C72	C73	172.4(3)
C76	C71	C72	C73	-0.4(5)
P1	C71	C76	C75	-172.3(3)
C72	C71	C76	C75	0.3(5)
C71	C72	C73	C74	0.1(5)
C72	C73	C74	C75	0.4(6)
C73	C74	C75	C76	-0.6(6)
C74	C75	C76	C71	0.2(6)
P1	C81	C82	C83	-179.0(3)
C86	C81	C82	C83	-1.2(6)
P1	C81	C86	C85	178.2(3)
C82	C81	C86	C85	0.5(6)
C81	C82	C83	C84	0.8(6)
C82	C83	C84	C85	0.3(7)
C83	C84	C85	C86	-1.0(7)
C84	C85	C86	C81	0.6(7)
C3S	O1S	C1S	C2S	-175.7(15)
C1S	O1S	C3S	C4S	-177.7(11)
C7S	O2S	C5S	C6S	-178(3)
C5S	O2S	C7S	C8S	170(3)

Table S18. Anisotropic Displacement Parameters (U_{ij}, Å^2).

Atom	U_{11}	U_{22}	U_{33}	U_{23}	U_{13}	U_{12}
Ru1	0.01938(10)	0.02134(10)	0.02760(10)	-0.00115(8)	0.00027(8)	0.00085(8)
Cl1	0.0629(6)	0.0589(5)	0.0314(4)	-0.0073(3)	-0.0055(4)	0.0186(5)
Cl2	0.0383(4)	0.0347(4)	0.0509(5)	0.0039(4)	-0.0016(4)	0.0125(3)
S1	0.0298(3)	0.0245(4)	0.0305(3)	-0.0004(3)	0.0001(3)	0.0019(3)
S2	0.0261(3)	0.0275(3)	0.0294(3)	-0.0023(3)	-0.0017(3)	0.0053(3)
P1	0.0190(3)	0.0291(4)	0.0267(3)	-0.0010(3)	0.0013(3)	0.0009(3)
N1	0.0229(11)	0.0214(12)	0.0317(13)	0.0000(9)	0.0018(9)	0.0026(9)
N2	0.0236(12)	0.0229(13)	0.0316(12)	-0.0022(10)	0.0006(10)	0.0015(10)
C1	0.0198(12)	0.0266(16)	0.0345(15)	-0.0038(12)	0.0038(11)	0.0002(11)
C2	0.0221(13)	0.0253(15)	0.0333(15)	-0.0028(12)	0.0019(11)	0.0025(11)
C3	0.0273(14)	0.042(2)	0.0335(15)	-0.0035(13)	0.0013(12)	0.0028(13)
C4	0.0327(16)	0.0374(19)	0.0403(17)	-0.0116(14)	0.0073(14)	0.0009(14)
C5	0.0294(16)	0.0278(17)	0.0489(19)	-0.0068(14)	0.0087(14)	0.0030(13)
C6	0.0250(14)	0.0281(16)	0.0432(17)	-0.0005(13)	0.0053(13)	0.0018(12)
C11	0.0194(12)	0.0156(12)	0.0336(15)	0.0000(10)	0.0015(11)	-0.0007(10)
C12	0.0332(15)	0.0259(15)	0.0365(16)	-0.0026(12)	0.0037(12)	0.0080(12)
C13	0.0370(16)	0.0218(15)	0.0380(16)	-0.0014(12)	-0.0017(13)	0.0063(13)
C21	0.0278(14)	0.0255(15)	0.0312(15)	0.0022(12)	0.0037(12)	0.0020(12)
C22	0.0279(14)	0.0338(16)	0.0302(14)	-0.0022(11)	0.0061(13)	-0.0001(14)
C23	0.0409(18)	0.045(2)	0.0314(16)	0.0011(14)	-0.0015(14)	-0.0008(15)
C24	0.052(2)	0.0406(19)	0.0363(16)	0.0113(15)	-0.0028(15)	0.0047(18)
C25	0.0447(19)	0.0296(16)	0.0442(19)	0.0071(14)	0.0043(15)	-0.0004(14)
C26	0.0306(15)	0.0292(17)	0.0368(16)	0.0020(12)	0.0052(12)	0.0005(13)
C27	0.0321(17)	0.0343(17)	0.0382(16)	-0.0053(13)	0.0040(14)	-0.0022(14)
C28	0.050(2)	0.049(2)	0.060(2)	-0.0168(19)	0.018(2)	-0.0007(19)
C29	0.042(2)	0.041(2)	0.061(2)	-0.0066(18)	-0.0076(18)	-0.0081(17)
C30	0.0276(14)	0.0279(16)	0.0477(18)	0.0060(13)	0.0053(13)	-0.0032(13)
C31	0.040(2)	0.047(2)	0.085(3)	-0.0033(19)	0.024(2)	0.0007(18)
C32	0.0368(17)	0.0374(19)	0.084(3)	-0.011(2)	0.008(2)	-0.0113(15)
C41	0.0255(14)	0.0279(16)	0.0327(15)	-0.0026(12)	-0.0046(12)	0.0064(12)
C42	0.0284(16)	0.0450(19)	0.0338(16)	0.0006(14)	-0.0042(13)	0.0025(14)
C43	0.0376(18)	0.063(2)	0.0346(17)	-0.0026(16)	-0.0025(15)	0.0030(17)
C44	0.049(2)	0.053(2)	0.0387(19)	-0.0131(16)	-0.0118(16)	0.0080(18)
C45	0.0403(18)	0.0396(19)	0.050(2)	-0.0102(16)	-0.0129(16)	0.0007(15)
C46	0.0318(16)	0.0259(16)	0.0438(18)	-0.0023(13)	-0.0061(13)	0.0026(13)
C47	0.0388(18)	0.074(3)	0.0290(15)	0.0058(15)	-0.0029(16)	-0.017(2)
C48	0.072(3)	0.065(3)	0.074(3)	-0.009(2)	-0.002(3)	-0.022(2)
C49	0.036(2)	0.099(4)	0.062(3)	0.020(3)	-0.0061(19)	-0.008(2)
C50	0.0333(18)	0.040(2)	0.053(2)	0.0031(16)	-0.0078(15)	-0.0086(14)
C51	0.0352(19)	0.069(3)	0.072(3)	-0.005(2)	0.000(2)	-0.0017(19)
C52	0.051(2)	0.048(3)	0.096(4)	0.011(2)	-0.007(2)	-0.014(2)
C61	0.0228(14)	0.0431(18)	0.0304(15)	-0.0059(12)	0.0000(11)	0.0047(12)
C62	0.0383(18)	0.0463(19)	0.0340(16)	-0.0007(14)	-0.0050(15)	-0.0019(16)
C63	0.050(2)	0.061(2)	0.0329(18)	0.0057(16)	-0.0052(16)	0.0086(19)
C64	0.0386(19)	0.088(3)	0.0344(19)	-0.0058(19)	-0.0105(15)	0.002(2)
C65	0.041(2)	0.071(3)	0.046(2)	-0.010(2)	-0.0112(17)	-0.0117(19)
C66	0.0338(17)	0.056(2)	0.0399(19)	-0.0032(16)	-0.0013(15)	-0.0083(16)
C71	0.0239(13)	0.0347(16)	0.0299(13)	0.0011(11)	-0.0026(12)	-0.0054(13)

Table S18. Anisotropic Displacement Parameters (continued).

Atom	U_{11}	U_{22}	U_{33}	U_{23}	U_{13}	U_{12}
C72	0.0264(14)	0.0356(17)	0.0388(17)	-0.0034(14)	0.0013(13)	-0.0033(12)
C73	0.0367(18)	0.0340(18)	0.051(2)	-0.0045(14)	-0.0005(14)	-0.0043(14)
C74	0.0425(19)	0.041(2)	0.0429(19)	0.0038(14)	-0.0023(15)	-0.0172(15)
C75	0.0281(16)	0.055(2)	0.0455(19)	0.0055(17)	0.0022(14)	-0.0127(16)
C76	0.0259(15)	0.044(2)	0.0436(18)	-0.0012(14)	0.0034(14)	-0.0004(14)
C81	0.0230(13)	0.0330(16)	0.0335(16)	-0.0019(12)	0.0034(12)	0.0003(12)
C82	0.0262(16)	0.055(2)	0.0360(17)	-0.0031(14)	0.0014(13)	0.0064(14)
C83	0.0361(18)	0.064(2)	0.0343(18)	-0.0085(16)	0.0057(15)	0.0022(17)
C84	0.0383(19)	0.053(2)	0.048(2)	-0.0147(17)	0.0082(16)	0.0099(17)
C85	0.049(2)	0.055(2)	0.051(2)	-0.0011(19)	0.0058(18)	0.025(2)
C86	0.046(2)	0.044(2)	0.0360(17)	-0.0012(14)	0.0003(15)	0.0175(16)
O1S	0.089(4)	0.042(3)	0.081(4)	0.018(3)	-0.008(3)	0.008(3)
C1S	0.135(11)	0.051(6)	0.092(9)	0.010(6)	-0.004(9)	-0.020(7)
C2S	0.095(12)	0.048(8)	0.173(17)	-0.010(8)	-0.007(13)	-0.016(9)
C3S	0.118(7)	0.086(7)	0.041(4)	0.005(4)	-0.013(5)	0.037(5)
C4S	0.094(7)	0.113(8)	0.059(5)	-0.012(5)	-0.025(5)	0.032(5)
O2S	0.074(9)	0.21(2)	0.104(13)	-0.032(13)	-0.036(9)	-0.008(10)
C5S	0.123(13)	0.106(18)	0.090(12)	-0.013(11)	-0.009(9)	-0.020(12)
C6S	0.076(13)	0.078(18)	0.115(16)	0.030(15)	0.004(11)	-0.002(11)
C7S	0.081(12)	0.30(4)	0.091(16)	0.031(19)	-0.029(11)	-0.052(16)
C8S	0.084(14)	0.38(5)	0.114(18)	0.08(2)	-0.013(13)	0.02(2)

The form of the anisotropic displacement parameter is:
$\exp[-2\pi^2(h^2a^{*2}U_{11} + k^2b^{*2}U_{22} + l^2c^{*2}U_{33} + 2klb^*c^*U_{23} + 2hla^*c^*U_{13} + 2hka^*b^*U_{12})]$

Table S19. Derived Atomic Coordinates and Displacement Parameters for Hydrogen Atoms.

Atom	*x*	*y*	*z*	U_{eq}, Å^2
H4	0.719822	0.068831	0.537715	0.044
H5	0.787902	0.007040	0.617778	0.042
H12A	0.486907	0.590706	0.779740	0.038
H12B	0.403622	0.520137	0.780704	0.038
H13A	0.375368	0.547812	0.688716	0.039
H13B	0.478038	0.600918	0.684837	0.039
H23	0.728465	0.439903	0.922080	0.047
H24	0.695822	0.305163	0.919086	0.051
H25	0.576498	0.254913	0.856481	0.047
H27	0.649400	0.580738	0.818586	0.042
H28A	0.507146	0.574773	0.880954	0.080
H28B	0.579414	0.647657	0.896978	0.080
H28C	0.583521	0.568810	0.933772	0.080
H29A	0.775229	0.564587	0.917365	0.072
H29B	0.770399	0.640852	0.877893	0.072
H29C	0.819373	0.560954	0.854377	0.072
H30	0.426963	0.367255	0.756060	0.041
H31A	0.368927	0.327953	0.869557	0.086
H31B	0.291217	0.354486	0.820731	0.086
H31C	0.372043	0.416143	0.846051	0.086
H32A	0.478886	0.231228	0.752443	0.079
H32B	0.357117	0.242770	0.758713	0.079
H32C	0.424809	0.218851	0.812464	0.079
H43	0.581739	0.498127	0.489740	0.054
H44	0.444978	0.417322	0.464440	0.056
H45	0.334161	0.368558	0.532804	0.052
H47	0.652699	0.550672	0.631612	0.057
H48A	0.624897	0.634775	0.528547	0.106
H48B	0.683448	0.667404	0.583155	0.106
H48C	0.561846	0.652388	0.585170	0.106
H49A	0.758923	0.464910	0.579982	0.098
H49B	0.803801	0.552475	0.577261	0.098
H49C	0.742897	0.516915	0.524396	0.098
H50	0.351926	0.402314	0.681782	0.051
H51A	0.228081	0.482987	0.635939	0.088
H51B	0.174945	0.408642	0.665372	0.088
H51C	0.192691	0.409424	0.598557	0.088
H52A	0.294652	0.282368	0.603184	0.098
H52B	0.268867	0.280247	0.669194	0.098
H52C	0.385829	0.274800	0.648285	0.098

Table S19. Derived Parameters for Hydrogen Atoms (continued).

Atom	*x*	*y*	*z*	U_{eq}, Å^2
H62	0.825352	0.268831	0.817453	0.047
H63	0.911711	0.260027	0.903801	0.058
H64	1.015104	0.364078	0.934356	0.064
H65	1.030971	0.476769	0.878437	0.063
H66	0.948609	0.483747	0.791423	0.052
H72	0.727739	0.535977	0.734681	0.040
H73	0.785059	0.663270	0.715965	0.049
H74	0.945027	0.682719	0.672254	0.051
H75	1.044912	0.575288	0.646756	0.052
H76	0.988328	0.448089	0.665738	0.045
H82	0.832102	0.353591	0.607474	0.047
H83	0.930346	0.273533	0.549107	0.054
H84	1.049394	0.186475	0.587959	0.056
H85	1.068240	0.179289	0.685339	0.062
H86	0.972259	0.261222	0.744333	0.051
H1SA^a	0.777887	0.086977	1.018222	0.111
H1SB^a	0.806774	0.037981	0.962518	0.111
H2SA^a	0.714263	0.129802	0.907589	0.158
H2SB^a	0.682952	0.175907	0.963897	0.158
H2SC^a	0.640716	0.088792	0.953082	0.158
H3SA^a	0.993928	0.066277	0.971217	0.098
H3SB^a	0.968529	0.107690	1.030351	0.098
H4SA^a	1.053763	0.187872	0.938105	0.133
H4SB^a	1.120461	0.153736	0.989329	0.133
H4SC^a	1.038575	0.222359	1.000376	0.133
H5SA^b	0.781497	0.194037	0.993683	0.128
H5SB^b	0.736140	0.121702	1.029441	0.128
H6SA^b	0.694143	0.139389	0.913901	0.134
H6SB^b	0.620942	0.176313	0.961322	0.134
H6SC^b	0.635726	0.083036	0.957490	0.134
H7SA^b	0.904742	0.077608	1.045250	0.189
H7SB^b	0.961556	0.139176	1.004143	0.189
H8SA^b	1.002608	0.033998	0.943731	0.287
H8SB^b	0.954340	-0.025257	0.989034	0.287
H8SC^b	1.054924	0.024663	1.004499	0.287

Refined occupancies of 0.614(10)^a and 0.386(10)^b.

S6. References

(1) Jang, W. J.; Kang, B.-N.; Lee, J. H.; Choi, Y. M.; Kim, C.-H.; Yun, J., NHC-copper-thiophene-2-carboxylate complex for the hydroboration of terminal alkynes. *Org. Biomol. Chem.* **2019**, *17*, 5249–5252.

(2) Hallman, P. S.; Stephenson, T. A.; Wilkinson, G., Tetrakis(triphenylphosphine)dichloro-ruthenium(II) and Tris(triphenylphosphine)dichlororuthenium(II). *Inorg. Synth.* **1970**, *12*, 237–240.

(3) Blanco, C. O.; Cormier, S. K.; Koller, A. J.; Boros, E.; Fogg, D. E., Olefin Metathesis in Water: Speciation of a Leading Water-Soluble Catalyst Pinpoints Challenges and Opportunities for Chemical Biology. *J. Am. Chem. Soc.* **2025**, *147*, 9441–9448.

(4) Lei, M.; Chen, X.; Wang, Y.; Zhang, L.; Zhu, H.; Wang, Z., Homogeneous and Heterogeneous Pd-Catalyzed Selective C–P Activation and Transfer Hydrogenation for "Group-Substitution" Synthesis of Trivalent Phosphines. *Org. Lett.* **2022**, *24*, 2868-2872.

(5) Grzesiński, Ł.; Nadirova, M.; Guschlbauer, J.; Brotons-Rufes, A.; Poater, A.; Kajetanowicz, A.; Grela, K., Preserving precise choreography of bonds in Z-stereoretentive olefin metathesis by using quinoxaline-2,3-dithiolate ligand. *Nat. Commun.* **2024**, *15*, 8981.

(6) Koh, M. J.; Khan, R. K. M.; Torker, S.; Yu, M.; Mikus, M. S.; Hoveyda, A. H., High-value alcohols and higher-oxidation-state compounds by catalytic Z-selective cross-metathesis. *Nature* **2015**, *517*, 181–185.

(7) Pardin, C.; Pelletier, J. N.; Lubell, W. D.; Keillor, J. W., Cinnamoyl Inhibitors of Tissue Transglutaminase. *J. Org. Chem.* **2008**, *73*, 5766–5775.

(8) Ahmed, T. S.; Grubbs, R. H., A Highly Efficient Synthesis of Z-Macrocycles Using Stereoretentive, Ruthenium-Based Metathesis Catalysts. *Angew. Chem., Int. Ed.* **2017**, *56*, 11213–11216.

(9) Blacquiere, J. M.; Jurca, T.; Weiss, J.; Fogg, D. E., Time as a Dimension in High-Throughput Homogeneous Catalysis. *Adv. Synth. Catal.* **2008**, *350*, 2849–2855.

(10) Pitto-Barry, A.; Lupan, A.; Zegke, M.; Swift, T.; Attia, A. A. A.; Lord, R. M.; Barry, N. P. E., Pseudo electron-deficient organometallics. *Dalton Trans.* **2017**, *46*, 15676–15683.

(11) Zeng, W.; Wang, E.; Qiu, R.; Sohail, M.; Wu, S.; Chen, F.-X., Oxygen-atom insertion of NHC–copper complex. *J. Organomet. Chem.* **2013**, *743*, 44–48.

(12) Boisvert, E. J.; Max, H.; Fogg, D. E., Rapid Aerial Oxidation of Ruthenium-Dithiocatecholate Catalysts: A Challenge to Stereoretentive Olefin Metathesis. *ACS Catal.* **2023**, *13*, 2885−2891.

(13) Nicholson, J. L.; Gravet, A. C.; Michaudel, Q., Telechelic all-cis polycyclooctene via catalytic stereoretentive ROMP for the synthesis of polylactide-based ABA triblock copolymers. *Faraday Discuss.* **2026**, *262*, 226–237.

(14) Cetinkaya, S.; Karabulut, S.; Duez, B.; Imamoglu, Y., Synthesis and characterization of polyoctenamer with WCl6-e--Al-CH2Cl2 catalyst system via ring-opening metathesis polymerization. *Appl. Organomet. Chem.* **2005**, *19*, 347–351.

(15) Keitz, B. K.; Fedorov, A.; Grubbs, R. H., Cis-Selective Ring-Opening Metathesis Polymerization with Ruthenium Catalysts. *J. Am. Chem. Soc.* **2012**, *134*, 2040–2043.

(16) Schuster, O.; Schmidbaur, H., Tetrakis(Triphenylphosphine Oxide)Lithium Di(Iodo)Aurate(I). *Z. Naturforsch. B* **2006**, *61*, 956–960.

(17) Sheldrick, G. M., SHELXT - Integrated Space-Group and Crystal-Structure Determination. *Acta Crystallogr., Sect. A* **2015**, *71*, 3–8.

(18) The disordered Et_2O molecule was restrained during refinement using the following *SHELXL* instructions: **SADI** (all C–O distances; all C–C distances); **RIGU** (all anisotropic displacement parameters for the C and O atoms); **ISOR** (all ADPs for the C and O atoms of the minor orientation O2S, C5S, C6S, C7S, C8S); and **SIMU** (ADPs for C2S and C6S). [g]Flack, H. D. *Acta Crystallogr.* **1983**, *A39*, 876–881; Flack, H. D.; Bernardinelli, G. *Acta Crystallogr.* **1999**, *A55*, 908–915; Flack, H. D.; Bernardinelli, G. *J. Appl. Cryst.* **2000**, *33*, 1143–1148. The Flack parameter will refine to a value near 0 if the structure is in the correct configuration, and a value near 1 for the inverted configuration.
